# KIF20A inhibits TRIM21-dependent ubiquitination of DHX9 to boost SOX2 stability, enhancing OSCC stemness and ferroptosis resistance

**DOI:** 10.1038/s41419-026-08467-w

**Published:** 2026-02-11

**Authors:** Ziyun Zhang, Yi Li, Jingjiang Hu, Xingjie Tang, Zhanpeng Li, Xiaoyan Sun, Dade Feng, Yixin Yao, Chao Mao, Yongguang Tao, Li Xie, Huaiqing Luo, Yufei Li, Xing Yu, Xiaoning Peng, Li Cong, Yiqun Jiang

**Affiliations:** 1 School of Basic Medical Sciences, Hunan Normal University Health Science Center, Hunan Normal University, Changsha, Hunan China; 2 Key Laboratory of Model Animals and Stem Cell Biology in Hunan Province, Hunan Normal University School of Medicine, Engineering Research Center of Reproduction and Translational Medicine of Hunan Province, Manufacture-Based Learning and Research Demonstration Center for Human Reproductive Health New Technology of Hunan Normal University, Changsha, Hunan China; 3 Department of Pathology, Nanjing Drum Tower Hospital, the Affiliated Hospital of Nanjing University Medical School, School of Life Sciences, Nanjing University, Nanjing, China; 4 Department of Pathology, Key Laboratory of Carcinogenesis and Cancer Invasion, Ministry of Education, Xiangya Hospital, School of Basic Medicine, Central South University, Changsha, Hunan China; 5 Department of Head and Neck Surgery, Hunan Cancer Hospital, Xiangya School of Medicine, Central South University, Changsha, Hunan China; 6 Hunan Key Laboratory of Immunology and Transmission Control of Schistosomiasis, Changsha, Hunan China; 7 Changsha Gene Editing Technique Innovation Center, Hunan Normal University, Changsha, Hunan China

**Keywords:** Oral cancer, Oral cancer

## Abstract

Oral squamous cell carcinoma (OSCC) is an aggressive malignancy characterized by poor prognosis, largely attributable to cancer stem cell (CSC) persistence and ferroptosis resistance. However, the molecular mechanisms that coordinately regulate stemness maintenance and ferroptosis suppression in OSCC remain insufficiently characterized. In this study, KIF20A was identified as significantly overexpressed in OSCC and strongly correlated with adverse clinical outcomes. An integrative approach identified DHX9 as a candidate interactor of KIF20A. Mechanistic investigations revealed that KIF20A regulates DHX9 nucleocytoplasmic distribution and inhibits TRIM21-mediated K48-linked polyubiquitination at DHX9-K755, thereby preventing its proteasomal degradation and enhancing protein stability. Elevated DHX9 enhanced SOX2 mRNA stability, leading to upregulation of SOX2, a central regulator of both CSC maintenance and ferroptosis resistance. Functionally, KIF20A promoted CSC phenotypes, inhibited ferroptosis in vitro and in vivo, and activated the PI3K/AKT signaling pathway. Notably, treatment with ENMD-2076 (identified through Connectivity Map analysis) significantly reduced KIF20A expression, attenuated CSC characteristics, augmented cisplatin sensitivity, and exerted marked antitumor activity. These findings elucidate a novel KIF20A-DHX9-SOX2 regulatory axis that simultaneously governs CSC maintenance and ferroptosis evasion in OSCC. Targeting KIF20A, either as a monotherapy or in combination with chemotherapy, may offer a promising strategy to improve therapeutic outcomes in OSCC.

## Introduction

According to the latest data in 2022, there were 389,846 new cases of lip and oral cavity cancer, with oral squamous cell carcinoma (OSCC) accounting for more than 90% of all oral malignancies, posing a significant burden on global health [1–4]. The progression and aggressiveness of OSCC are tightly linked to cancer stem cells (CSCs) [5]. Ferroptosis is a regulated form of iron-dependent cell death characterized by the accumulation of lipid peroxides due to oxidative stress [6]. Emerging evidence reveals a mechanistic convergence between cancer stemness regulatory networks and ferroptosis pathways [7, 8]. Mapping their interconnectivity may identify novel targets to disrupt CSCs-fueled tumor evolution and treatment failure.

KIF20A (Kinesin Family Member 20A) is a motor protein that plays a critical role in mitosis and intracellular transport [9–11]. Research has been increasingly recognized for its important roles in tumor pathology. It is reported to be highly expressed in multiple cancers, including pancreatic [12, 13], breast [14], lung [15], ovarian [16], and hepatocellular carcinoma [17], and correlated with poor prognosis, tumor progression, metastasis, and immune evasion [18, 19]. Moreover, KIF20A is overexpressed in glioma stem cells and connected to cancer stemness [20]. KIF20A stabilizes cancer stemness drivers like MYC through competitively inhibiting FBXW7-mediated degradation [21], while its depletion sensitizes lung adenocarcinoma cells to gemcitabine and ferroptosis inducer co-treatment via lipid peroxidation potentiation [1]. These findings support the hypothesis that KIF20A serves as a shared molecular switch governing both stemness-associated survival and ferroptosis pathways.

RNA helicase DHX9 has been identified as a pivotal player in cancer progression, serving as a key regulator of mRNA stability, genome integrity, and cellular stress responses [22]. Its activity is modulated by ubiquitin ligases, which subsequently influence the growth and migration of cancer cells. In thyroid cancer, the E3 ubiquitin ligase MARCH6 enhances DHX9 activity, promoting the proliferation and migration of thyroid cancer cells [23]. LINC01016 prevents the E3 ubiquitin ligase RFFL from binding to DHX9, inhibiting its ubiquitin-mediated degradation and leading to increased DHX9 expression. This stabilization of DHX9 activates the PI3K/AKT signaling pathway, promoting breast cancer progression [24]. These findings highlight ubiquitination as a pivotal post-translational modification governing DHX9 protein stability and functional regulation.

DHX9 plays a critical role in maintaining cellular stemness by distinct mechanisms in normal and cancer contexts. In intestinal stem cells (ISCs), DHX9 deficiency triggers R-loop accumulation, genomic instability, and cGAS-STING-mediated inflammation, impairing ISC function and epithelial homeostasis [25]. Conversely, in glioma stem cells, DHX9 interacts with IGF2BP2 to enhance the stability of SOX2 mRNA [26], a pivotal transcription factor that governs CSC properties. SOX2 serves as a key stemness marker across multiple tumor types by facilitating self-renewal, therapy resistance, and tumor progression [27]. Furthermore, SOX2 expression is regulated by the Lin28B/Let-7 axis and plays a critical role in reprogramming OSCC cells into a stem-like state [28].

Beyond its well-established role in maintaining CSC stemness, SOX2 has also played a crucial role in ferroptosis regulation, which involves key regulators such as GPX4 [29, 30], SLC7A11 (xCT) [31, 32], and lipid metabolism pathways [33, 34]. By directly binding to the SLC7A11 promoter, SOX2 activates its transcription, thereby promoting both stemness and ferroptosis resistance in lung cancer stem-like cells. Notably, oxidation at Cys265 inhibits SOX2 activity, leading to reduced self-renewal capacity and increased ferroptosis sensitivity [35].

Despite growing research on cancer stemness and ferroptosis, the crosstalk between stemness regulatory circuits and ferroptosis pathways, along with their underlying molecular mechanisms, remains an open frontier for systematic investigation. In this study, we propose that KIF20A orchestrates OSCC stemness and ferroptosis through DHX9-dependent SOX2 regulation. Additionally, bioinformatics-based screening of small-molecule compounds negatively correlated with KIF20A expression will lay the groundwork for novel therapeutic strategies to enhance cisplatin-based chemotherapy efficacy.

## Results

### KIF20A expression is elevated in OSCC and associated with poor prognosis

**Fig. 1:**
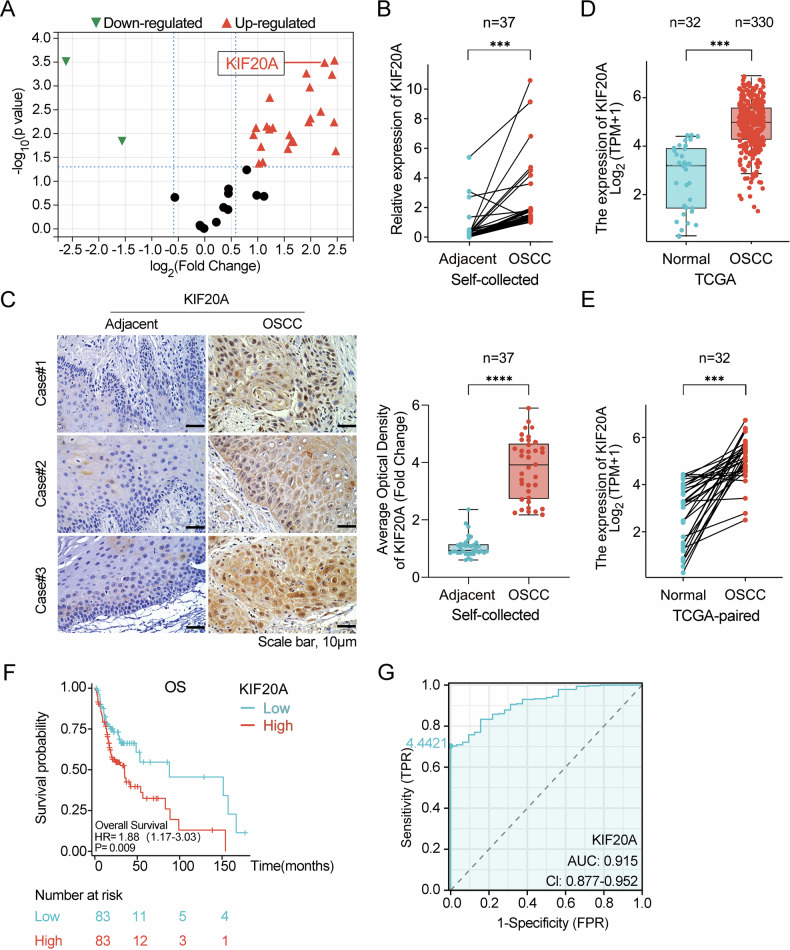
KIF20A expression is elevated in OSCC and associated with poor prognosis. **A** A volcano plot illustrating the differential expression of driver protein family members, including KIF20A, between paired OSCC and adjacent non-cancerous tissues (*n* = 5 per group). **B** The relative mRNA expression levels of KIF20A in paired OSCC and adjacent non-cancerous tissues were measured by RT-qPCR (*n* = 37 per group). **C** Immunohistochemical (IHC) analysis of KIF20A expression in paired OSCC and adjacent tissues (*n* = 37 per group). **D**, **E** TCGA dataset analysis comparing the mRNA expression levels of KIF20A in OSCC (*n* = 330) and normal tissues (*n* = 32). The expression levels are shown as box plots (**D**) and paired comparisons (**E**). **F** Kaplan–Meier survival analysis for TCGA-OSCC patients with high and low KIF20A expression (group cutoff high 75%, low 25%). **G** Receiver operating characteristic (ROC) curve analysis for KIF20A expression. Statistical significance is denoted by ****P < 0.0001, ***P < 0.001.

A total of five paired OSCC samples, comprising cancerous and corresponding adjacent non-cancerous tissues, were collected for RNA sequencing to examine the expression profile of the kinesin family members. The analysis revealed that, compared to adjacent non-cancerous tissues, the expression levels of many driver protein family members, including KIF20A (log_2_FC = 2.26, *P* = 0.0003), were significantly elevated in OSCC tissues (Fig. 1A). Upon expanding the self-collected OSCC sample size to 37 pairs, RT-qPCR and IHC were employed to confirm the high expression of KIF20A in OSCC (Fig. 1B, C). Further analysis of the TCGA dataset, encompassing both OSCC and normal tissue samples, validated that KIF20A expression is significantly higher in OSCC than in normal tissues (Fig. 1D, E). More over TCGA-OSCC patients with high KIF20A expression exhibited significantly poorer overall survival (OS) compared to those with low expression, with an AUC value of 0.915 (Fig. 1F, G). These findings strongly indicate that elevated KIF20A expression is closely associated with poor prognosis in OSCC patients.

### KIF20A interacts with DHX9 and upregulates its expression at the post-translational level

**Fig. 2:**
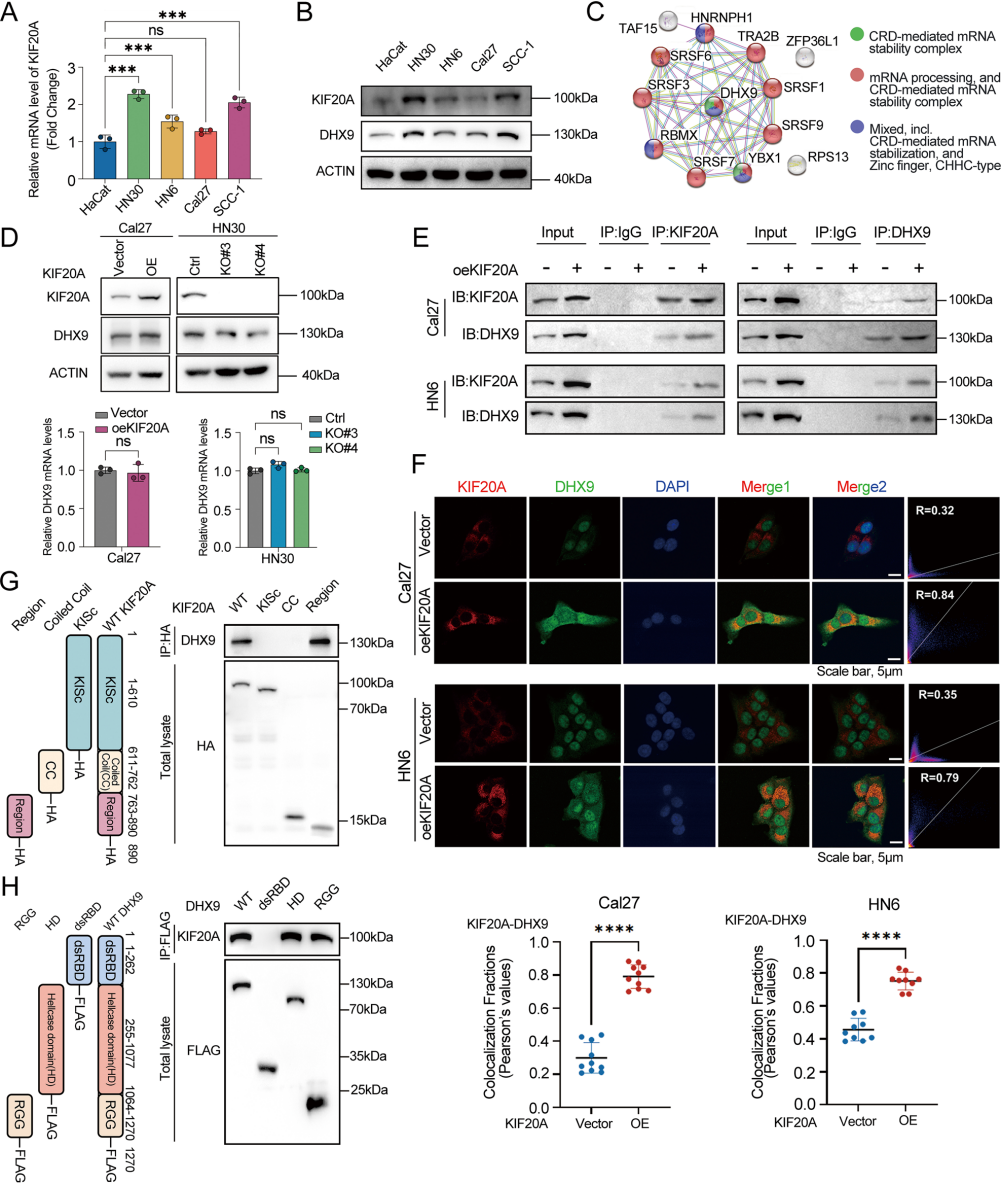
KIF20A interacts with DHX9 and upregulates its expression. **A** RT-qPCR analysis of KIF20A mRNA levels in OSCC cell lines (HN30, SCC-1, Cal27, and HN6) compared to normal HaCaT cells. **B** Western blot analysis showing the expression levels of KIF20A and DHX9 in OSCC cell lines (HN30, SCC-1, Cal27, and HN6) and normal HaCaT cells. **C** STRING protein interaction network diagram for the 13 intersecting proteins in Supplementary Fig. 1B. **D** Western blot analysis of the expression levels of KIF20A and DHX9 in stable KIF20A high-expressing Cal27 cells and stable KIF20A knockout HN30 cells, as well as RT-qPCR analysis of the mRNA level of DHX9. **E** Co-immunoprecipitation (Co-IP) and Western blot assays confirm the interaction between KIF20A and DHX9. **F** Immunofluorescence staining of KIF20A (red), DHX9 (green), and DAPI (blue) in Cal27 and HN6 cells with stable KIF20A overexpression. Scale bar, 5 μm. Bottom panel: dot plots showed quantification of colocalization (Pearson correlation coefficient). **G**, **H** Schematic representations of full-length and deletion mutants of KIF20A and DHX9 (left panels). Co-IP assays were performed to study the interaction between KIF20A and DHX9 (right panels). HEK293T cells were transfected with (**G**) HA-tagged full-length or deletion mutants of KIF20A, or (**H**) FLAG-tagged full-length or deletion mutants of DHX9. Cell lysates were collected for Co-IP analysis, and the bound KIF20A or DHX9 proteins were detected by immunoblotting. Full-length and deletion mutants of KIF20A and DHX9 were successfully identified. Statistical significance is denoted by ****P < 0.0001, ***P < 0.001, ns not significant.

To elucidate the biological functions and underlying molecular mechanisms of KIF20A in OSCC, we initially investigated its proteomic interactome. Expression profiling revealed that KIF20A was markedly upregulated in OSCC cell lines compared to immortalized human keratinocytes (HaCaT). In particular, HN30 and SCC-1 cells exhibited the highest levels of KIF20A, while relatively lower expression was observed in Cal27 and HN6 (Fig. 2A, B). To identify potential KIF20A-interacting proteins, we performed co-immunoprecipitation in HN30 followed by mass spectrometry (CoIP/MS). The resulting protein list was subjected to functional enrichment analysis via Metascape (https://metascape.org/), which revealed that the top three ranked clusters (R-HAS-8953854, GO:0006397, GO:00043484) were all significantly enriched in biological processes related to mRNA regulation. Intersecting these clusters yielded 13 common proteins, which were subsequently analyzed using the STRING database to construct a protein–protein interaction (PPI) network. Within this network, DHX9 emerged as a central and prominent candidate (Fig. 2C and Supplementary Fig. 1).

In parental OSCC cell lines, DHX9 protein levels closely paralleled those of KIF20A (Fig. 2B). Functional assays further demonstrated that overexpression of KIF20A in Cal27 and HN6 cells led to a notable increase in DHX9 protein expression, whereas CRISPR/Cas9-mediated knockout of KIF20A in HN30 and SCC-1 cells resulted in a significant reduction of DHX9 protein levels. Importantly, these changes were observed at the protein level, with no significant alterations at the mRNA level, suggesting post-transcriptional regulation (Fig. 2D and Supplementary Fig. 2A). CoIP-Western blot and confocal immunofluorescence microscopy further confirmed enhanced interaction and subcellular colocalization between KIF20A and DHX9 upon KIF20A overexpression in Cal27 and HN6 cells (Fig. 2E, F). Conversely, in KIF20A-knockout HN30 and SCC-1 cells, the subcellular colocalization with DHX9 were largely reduced (Supplementary Fig. 2B). Moreover, confocal analysis revealed that KIF20A overexpression promoted cytoplasmic redistribution of DHX9, increasing the cytoplasmic-to-nuclear ratio, whereas KIF20A depletion had the opposite effect (Supplementary Fig. 2C). To further substantiate the immunofluorescence findings, cytoplasmic–nuclear fractionation assays were performed in KIF20A-overexpressing and KIF20A-knockout cells, followed by Western blot analysis of DHX9 distribution. The results showed that KIF20A overexpression markedly increased the cytoplasmic-to-nuclear ratio of DHX9, whereas KIF20A depletion produced the opposite effect. These data corroborate the confocal microscopy observations and provide quantitative evidence that KIF20A regulates the nucleocytoplasmic distribution of DHX9 (Supplementary Fig. 9).

To identify the specific interaction domains between KIF20A and DHX9, we transfected HEK293T cells with a series of C-terminally HA-tagged KIF20A truncations and C-terminally Flag-tagged DHX9 constructs. KIF20A, a protein comprising 890 amino acids, consists of an N-terminal kinesin motor domain (KISc), a central coiled-coil (CC) domain, and a C-terminal regulatory tail region [36]. Co-immunoprecipitation (Co-IP) analysis of these truncation mutants revealed that KIF20A primarily interacts with DHX9 via its C-terminal tail region (Fig. 2G). To further map the binding interface on DHX9, we generated three truncations, including the double-stranded RNA-binding domain (dsRBD) at the N-terminus, the helicase domain, and a C-terminal fragment containing nuclear localization/export signals along with repeated arginine-glycine-glycine (RGG) motifs [24]. Co-IP analysis of DHX9 truncations demonstrated that the helicase domain and RGG-containing C-terminal region mediate the interaction with KIF20A (Fig. 2H). These findings indicate that the C-terminal tail of KIF20A interacts with the helicase domain and RGG motifs of DHX9, implicating a potential post-translational regulatory mechanism by which KIF20A modulates DHX9 protein stability or function.

### KIF20A inhibits ubiquitin-mediated DHX9 degradation

**Fig. 3:**
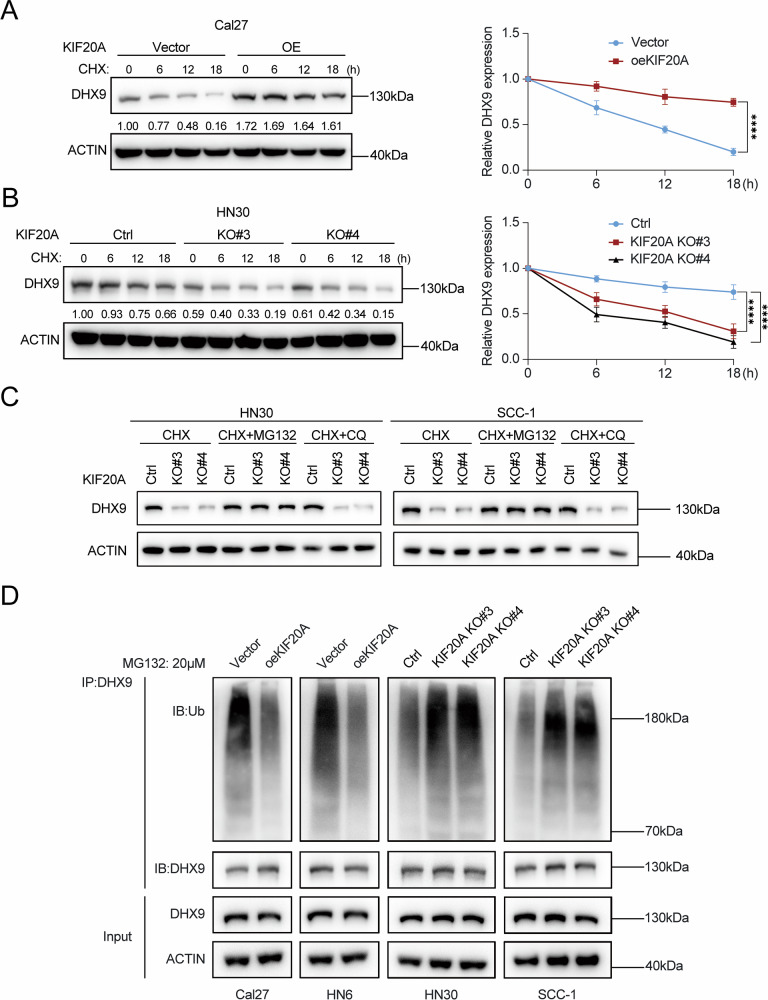
KIF20A promotes the expression of DHX9 by inhibiting its ubiquitin-mediated degradation. **A** Western blot analysis showing the changes in DHX9 protein levels in stable KIF20A-overexpressing Cal27 cells and control cells following CHX treatment at different time points, along with the corresponding quantification. **B** Western blot analysis shows the changes in DHX9 protein levels in stable KIF20A-knockout HN30 cells and control cells following CHX treatment at different time points, along with the corresponding quantification. **C** Western blot analysis of DHX9 protein levels in stable KIF20A-knockout HN30 and SCC-1 cells treated with CHX in combination with MG132 or CQ. **D** Western blot analysis of DHX9 ubiquitination levels in cells with KIF20A overexpression or knockout. Statistical significance is denoted by ****P < 0.0001.

To further elucidate the mechanism by which KIF20A upregulates DHX9 at the post-translational level, we performed protein stability assays using the protein synthesis inhibitor cycloheximide (CHX). KIF20A-overexpressing and knockout OSCC cell lines, along with their respective controls, were treated with CHX to monitor DHX9 degradation dynamics. In Cal27 and HN6 cells, KIF20A overexpression significantly delayed DHX9 degradation, indicating enhanced protein stability (Fig. 3A and Supplementary Fig. 3A). Conversely, KIF20A knockout in HN30 and SCC-1 cells accelerated DHX9 degradation, suggesting that KIF20A plays a protective role in maintaining DHX9 protein levels (Fig. 3B and Supplementary Fig. 3B).

As protein degradation is primarily mediated by the ubiquitin-proteasome system [37] and the lysosomal pathway [38], we next investigated the specific proteolytic route involved in KIF20A-mediated DHX9 stabilization. To this end, CHX-treated KIF20A-knockout and control cells were co-treated with the proteasome inhibitor MG132 or the lysosomal inhibitor chloroquine (CQ). The results revealed that MG132 markedly inhibited DHX9 degradation, whereas CQ had no significant effect (Fig. 3C), implicating the ubiquitin-proteasome pathway as the primary mechanism.

Further supporting this conclusion, KIF20A overexpression in Cal27 and HN6 cells suppressed DHX9 ubiquitination, whereas KIF20A knockout in HN30 and SCC-1 significantly enhanced DHX9 ubiquitination (Fig. 3D). These findings indicate that KIF20A stabilizes DHX9 by inhibiting its ubiquitin-proteasome-mediated degradation, revealing a novel post-translational regulatory mechanism by which KIF20A controls DHX9 protein homeostasis.

### KIF20A suppresses TRIM21-mediated K48-linked polyubiquitination of DHX9 at K755

**Fig. 4:**
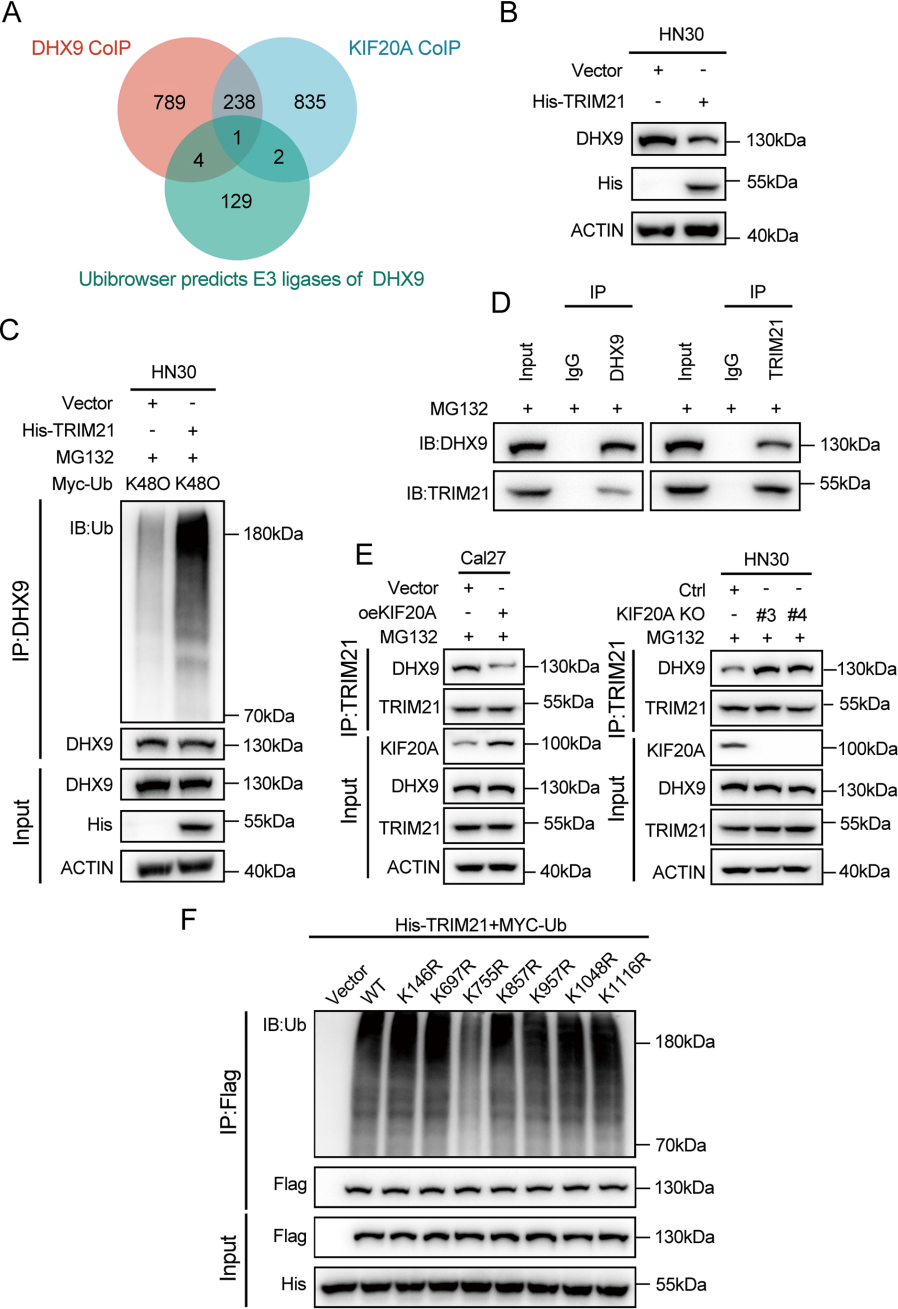
KIF20A stabilizes DHX9 by impairing TRIM21-mediated K48-linked polyubiquitination. **A** Venn diagram illustrating the overlap among DHX9-associated E3 ubiquitin ligases predicted by UbiBrowser and KIF20A- and DHX9-interacting proteins. **B** Western blot analysis of DHX9 ubiquitination in HN30 cells overexpressing TRIM21. **C** CoIP/WB analysis of DHX9 ubiquitination in HN30 cells overexpressing TRIM21. Cells were transfected with Myc-tagged K48-only ubiquitin mutant along with TRIM21 or vector, followed by MG132 treatment (10 μM, 8 h) prior to harvest. **D** CoIP/WB revealed the interaction between DHX9 and TRIM21 in HN30 cells. Cells were treated with MG132 (10 μM) for 8 h before harvest. **E** CoIP/WB analysis of TRIM21-mediated DHX9 ubiquitination in Cal27 cells stably overexpressing KIF20A and in HN30 cells with KIF20A knockout. **F** CoIP/WB analysis of DHX9 ubiquitination in HEK293T cells transfected with Flag-tagged wild-type or lysine-to-arginine (K → R) DHX9 mutants, along with His-TRIM21 and Myc-tagged ubiquitin.

As demonstrated by the preceding results, KIF20A promotes DHX9 expression by suppressing its ubiquitination. To further elucidate the underlying molecular mechanism, we sought to identify the E3 ubiquitin ligase responsible for targeting DHX9. Candidate E3 ligases were screened by integrating data from Co-IP-based proteomic analysis and UbiBrowser 1.0 predictions. A Venn diagram analysis revealed that TRIM21 was the only protein common to the set of KIF20A-interacting partners, DHX9-binding proteins, and predicted DHX9-specific E3 ligases (Fig. 4A and Supplementary Fig. 4).

Functional validation in HN30 cells showed that TRIM21 overexpression significantly reduced DHX9 protein levels, suggesting that TRIM21 promotes DHX9 degradation (Fig. 4B). Given that K48-linked polyubiquitin chains are widely recognized as the classical degron signal for proteasomal degradation, we next evaluated whether TRIM21 facilitates K48-linked ubiquitination of DHX9. TRIM21 markedly enhanced K48-linked polyubiquitination of DHX9 (Fig. 4C), indicating that DHX9 degradation is K48-linked polyubiquitin–dependent. Furthermore, Co-IP assays confirmed a physical interaction between TRIM21 and DHX9 (Fig. 4D).

To determine whether KIF20A modulates TRIM21-mediated ubiquitination of DHX9, we examined whether KIF20A interferes with the interaction between TRIM21 and DHX9. As shown in Fig. 4E, KIF20A overexpression in Cal27 cells markedly reduced the association between TRIM21 and DHX9, whereas KIF20A knockout in HN30 cells enhanced their interaction. These findings indicate that KIF20A negatively regulates TRIM21-mediated ubiquitination, thereby contributing to DHX9 protein stabilization.

Based on UbiBrowser predictions, seven lysine residues (K146, K697, K755, K857, K957, K1048, and K1116) were identified as putative ubiquitination sites. To validate their functional relevance, K-to-R point mutations were generated for each site. HEK293T cells were co-transfected with plasmids encoding wild-type or mutant DHX9, along with MYC-tagged ubiquitin and His-tagged TRIM21. Ubiquitination assays revealed that mutation of K755 significantly reduced TRIM21-mediated ubiquitination of DHX9 (Fig. 4F), suggesting that K755 is a critical acceptor site for TRIM21-directed K48-linked polyubiquitination.

### KIF20A enhances the cancer stemness in OSCC

**Fig. 5:**
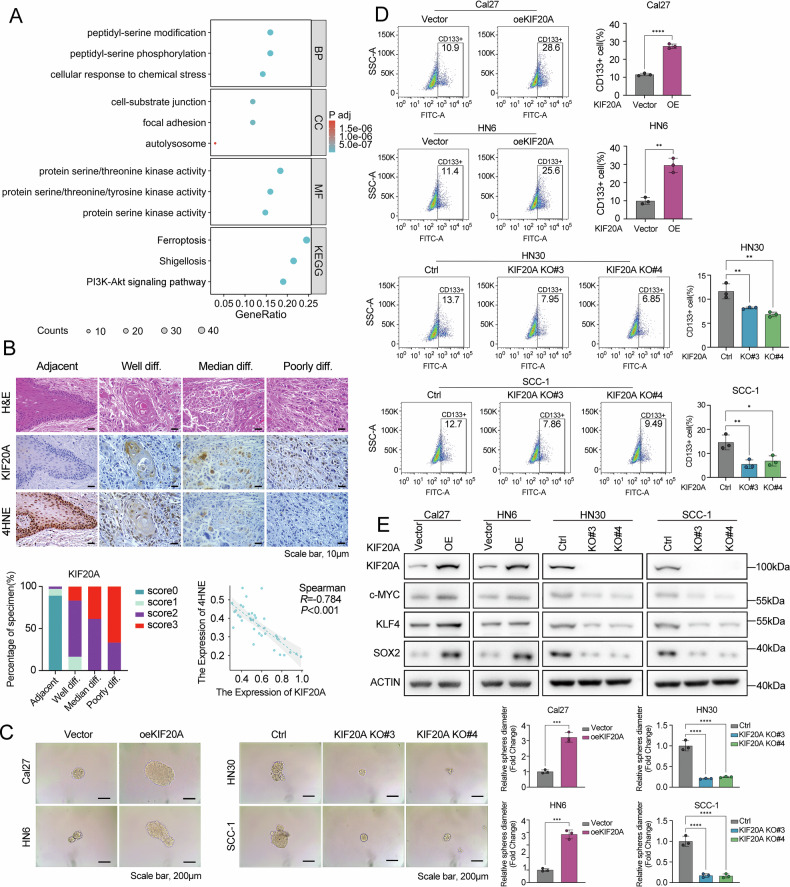
KIF20A evaluates the cancer stemness of OSCC. **A** GO and KEGG enrichment analyses of TCGA-OSCC RNA-seq data. Bubble plots show significantly enriched biological processes and pathways, including PI3K-AKT signaling and ferroptosis. **B** Representative H&E staining, KIF20A, and 4HNE immunohistochemistry (IHC) in OSCC tissues with varying degrees of differentiation (well, moderately, and poorly differentiated) and adjacent normal tissues. Bar graph shows the percentage distribution of KIF20A IHC scores (0–3) across different differentiation grades. The scatter plot depicts the correlation between KIF20A and 4-HNE expression levels. (*n* = 37 per group). **C** Tumorsphere formation assay evaluating the tumorsphere-forming ability of stable KIF20A-overexpressing and knockout cell lines. **D** Flow cytometry analysis showing the proportion of CD133^+^ stem-like cells in stable KIF20A-overexpressing and knockout cell lines. **E** Western blot analysis of stemness markers, including c-MYC, KLF4, and SOX2, in stable KIF20A-overexpressing and knockout cell lines. Statistical significance is denoted by ****P < 0.0001, ***P < 0.001, **P < 0.01, *P < 0.05.

To explore the broader functional landscape of KIF20A in OSCC, we performed GO and KEGG enrichment analyses based on KIF20A-associated gene expression profiles to identify biological pathways and processes associated with KIF20A expression. The results revealed that KIF20A-related genes were significantly enriched in pathways involved in mRNA processing, kinase activity, cell adhesion, and notably, ferroptosis and the PI3K-AKT signaling pathway (Fig. 5A). Additionally, IHC analysis of OSCC samples revealed that KIF20A expression was negatively correlated with tumor differentiation grade and lipid peroxidation marker 4-hydroxynonenal (4HNE), suggesting its potential involvement in tumor stemness and ferroptosis (Fig. 5B).

To validate these findings functionally, we established stable KIF20A-overexpressing and knockout cell lines. CCK-8 proliferation assays showed that KIF20A overexpression significantly enhanced cell proliferation, while KIF20A knockout markedly suppressed proliferation (Supplementary Fig. 5A). Consistently, Transwell migration and invasion assays demonstrated that KIF20A promotes cell motility and invasiveness, whereas its loss inhibits these phenotypes (Supplementary Fig. 5B).

To evaluate the role of KIF20A in cancer stemness, we performed tumorsphere formation assays, a widely recognized method for assessing stem-like properties in cancer cells. KIF20A overexpression in Cal27 and HN6 cells markedly increased tumorsphere formation, while KIF20A knockout in HN30 and SCC-1 significantly reduced this capacity (Fig. 5C). Furthermore, flow cytometry analysis revealed that KIF20A overexpression elevated the proportion of CD133-positive cells, a well-established marker of CSCs [39], while KIF20A knockout reduced this population (Fig. 5D). Western blot analysis further supported these findings, showing that KIF20A overexpression upregulated key stemness-related transcription factors, including c-MYC, KLF4, and SOX2, whereas KIF20A depletion significantly suppressed their expression levels (Fig. 5E).

Given our previous finding that KIF20A interacts with DHX9, we next examined whether this interaction functionally contributes to the observed stem-like phenotypes. Reciprocal rescue assays were conducted in vitro, in which DHX9 was silenced in KIF20A-overexpressing cells, whereas full-length DHX9 was reintroduced into KIF20A-knockout cells. Phenotypic outcomes were evaluated using CCK-8 assays, extreme limiting dilution analysis (ELDA), and tumorsphere formation assays. DHX9 knockdown in KIF20A-overexpressing cells markedly reduced cell viability, tumorsphere formation capacity, and stem cell frequency, whereas re-expression of full-length DHX9 in KIF20A-deficient cells restored cell proliferation, sphere size, and stem cell frequency (Supplementary Fig. 10). These findings highlight that the KIF20A–DHX9 axis functionally supports OSCC stemness by maintaining proliferative and self-renewal capacities.

### KIF20A promotes resistance to ferroptosis in OSCC

**Fig. 6:**
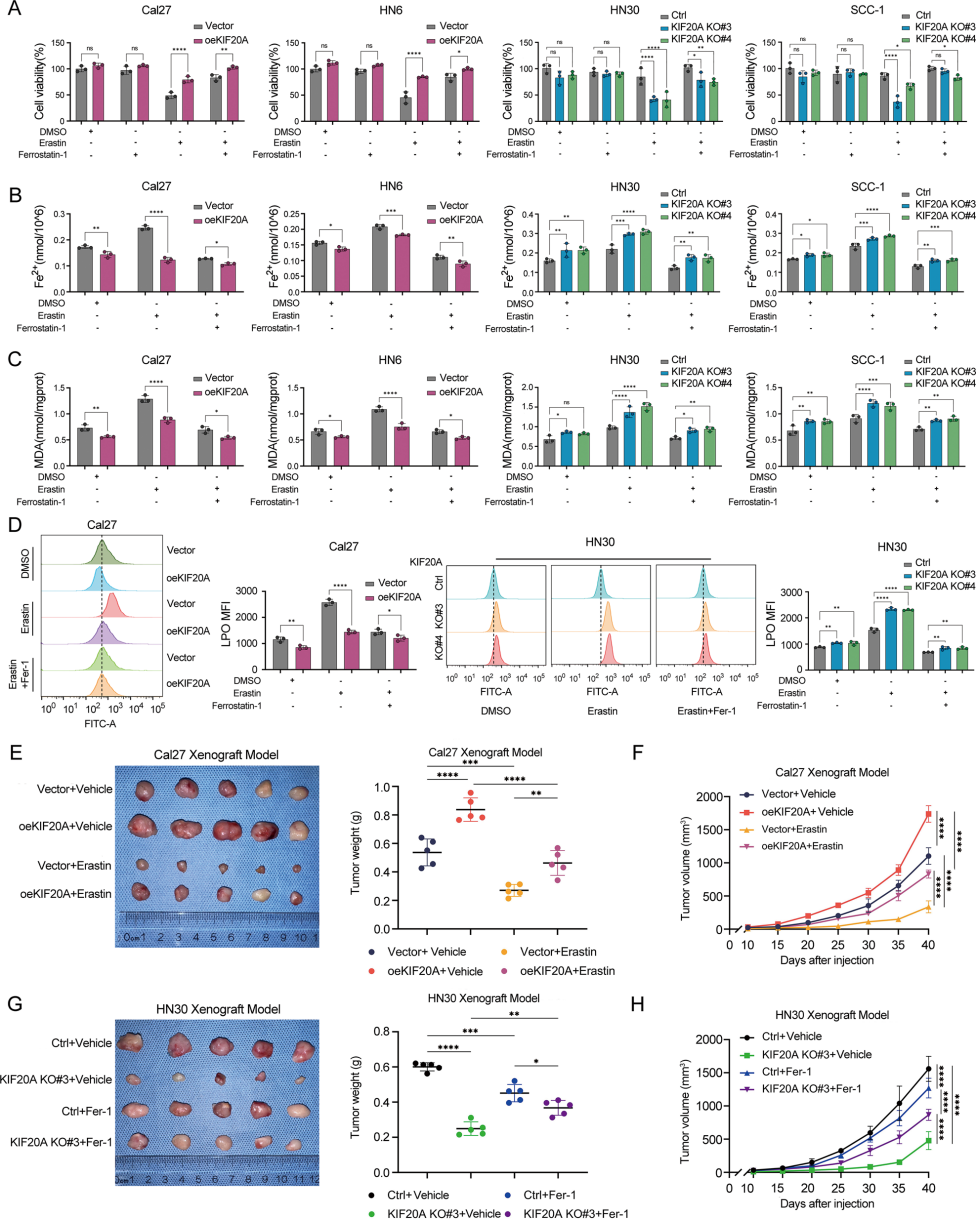
KIF20A enhances the stemness of OSCC and inhibits ferroptosis. **A** CCK-8 assay measuring cell viability in stable KIF20A-overexpressing and knockout cell lines treated with 20 µM Erastin, 10 µM Fer-1, or their combination. **B** Ferrous iron levels were detected using a ferrous iron assay kit in stable KIF20A-overexpressing and knockout cell lines treated with Erastin or the combination of Erastin and Fer-1. **C** Malondialdehyde (MDA) levels were measured using an MDA assay kit in stable KIF20A-overexpressing and knockout cell lines treated with Erastin or the combination of Erastin and Fer-1. **D** Lipid peroxidation levels were assessed by flow cytometry after treatment with Liperfluo reagent in stable KIF20A-overexpressing and knockout cell lines. Xenograft experiments showing tumor images and tumor weight statistics (**E**), as well as tumor growth curves (**F**), for Cal27 tumors with KIF20A overexpression treated with Erastin (*n* = 5 per group). Xenograft experiments showing tumor images and tumor weight statistics (**G**), as well as tumor growth curves (**H**), for HN30 tumors with KIF20A knockout treated with Fer-1 (*n* = 5 per group). Statistical significance is denoted by ****P < 0.0001, ***P < 0.001, **P < 0.01, *P < 0.05, ns not significant.

Given the observed association between KIF20A expression and ferroptosis-related signatures (Fig. 5A, B), we next investigated the functional role of KIF20A in regulating ferroptosis. Using Erastin, a well-characterized ferroptosis inducer, and Ferrostatin-1 (Fer-1), a specific ferroptosis inhibitor, CCK-8 assays demonstrated that KIF20A overexpression significantly attenuated Erastin-induced ferroptotic cell death, whereas KIF20A knockout markedly increased ferroptosis sensitivity. Notably, treatment with Fer-1 rescued Erastin-mediated growth inhibition, underscoring the regulatory role of KIF20A in ferroptotic resistance (Fig. 6A).

To further validate this role, we measured intracellular levels of ferrous iron (Fe²⁺), malondialdehyde (MDA), and lipid peroxidation (LPO)—hallmark indicators of ferroptosis—in both KIF20A-overexpressing and knockout cell lines, following treatment with Erastin and/or Fer-1. KIF20A overexpression significantly reduced Fe²⁺, MDA, and LPO levels, indicating a suppressive effect on ferroptosis. In contrast, KIF20A deletion led to pronounced increases in Fe²⁺ (Fig. 6B), MDA (Fig. 6C), and LPO (Fig. 6D), suggesting a heightened susceptibility to ferroptotic stress. These results identify KIF20A as a key suppressor of ferroptosis, complementing its role in promoting OSCC stemness. As complementary validation, additional ferroptosis modulators were employed. In Cal27 cells, KIF20A overexpression significantly increased cell viability upon treatment with the GPX4 inhibitor RSL3 (10 μM, 12 h), indicating enhanced resistance to ferroptosis. Moreover, the RSL3-induced elevation of intracellular Fe²⁺ levels was markedly attenuated in KIF20A-overexpressing cells, suggesting that KIF20A mitigates iron accumulation during ferroptotic stress. Conversely, in KIF20A-knockout HN30 cells, exposure to RSL3 led to a pronounced reduction in viability and a concomitant increase in intracellular Fe²⁺ levels, consistent with increased ferroptosis sensitivity. Importantly, treatment with the iron chelator deferoxamine (DFO, 100 μM, 12 h) effectively restored cell viability and reduced Fe²⁺ accumulation to near-control levels (Supplementary Fig. 11). These supplementary results reinforce the conclusion that KIF20A enhances ferroptosis resistance in OSCC.

To assess the in vivo relevance of these findings, we established xenograft tumor models using Cal27 and HN30 cells, followed by treatment with Erastin, Fer-1, or vehicle controls. Female BALB/c nude mice were subcutaneously injected with either KIF20A-overexpressing Cal27 cells or control cells, and beginning on day 10 post-inoculation, were treated with Erastin (20 mg/kg) or vehicle every 4 days. In a parallel experiment, HN30-derived tumors with or without KIF20A knockout received Fer-1 (2 mg/kg) or vehicle on the same schedule. Tumors were harvested on day 40 for analysis. The results showed that KIF20A overexpression enhanced tumor growth and weight, while blunting the tumor-suppressive effects of Erastin-induced ferroptosis (Fig. 6E, F). Conversely, KIF20A deletion reduced tumor size and weight, which was rescued by Fer-1 treatment (Fig. 6G, H). Importantly, mouse body weights remained stable across all groups, suggesting minimal systemic toxicity (supplementary Fig. 6). These findings further support the role of KIF20A in ferroptosis resistance and tumor progression in vivo.

Moreover, KEGG and GSEA analysis revealed a strong positive association between KIF20A expression and the PI3K-AKT signaling pathway (Fig. 5A and Supplementary Fig. 7A), a well-established regulator of both cancer stemness [40] and ferroptosis resistance [41]. To probe this mechanistically, western blot was performed to evaluate the phosphorylation status of key pathway components. The results indicated that KIF20A overexpression in Cal27 and HN6 cells enhanced phosphorylation of AKT, PI3K, S6K1, and 4EBP1, whereas KIF20A knockout in HN30 and SCC-1 cells led to their significant downregulation (Supplementary Fig. 7B). These findings reveal that KIF20A promotes OSCC tumorigenesis by concurrently enhancing cancer stemness and inhibiting ferroptosis, likely through activation of the PI3K-AKT signaling axis.

### KIF20A regulates SOX2 expression in a DHX9-dependent manner

**Fig. 7:**
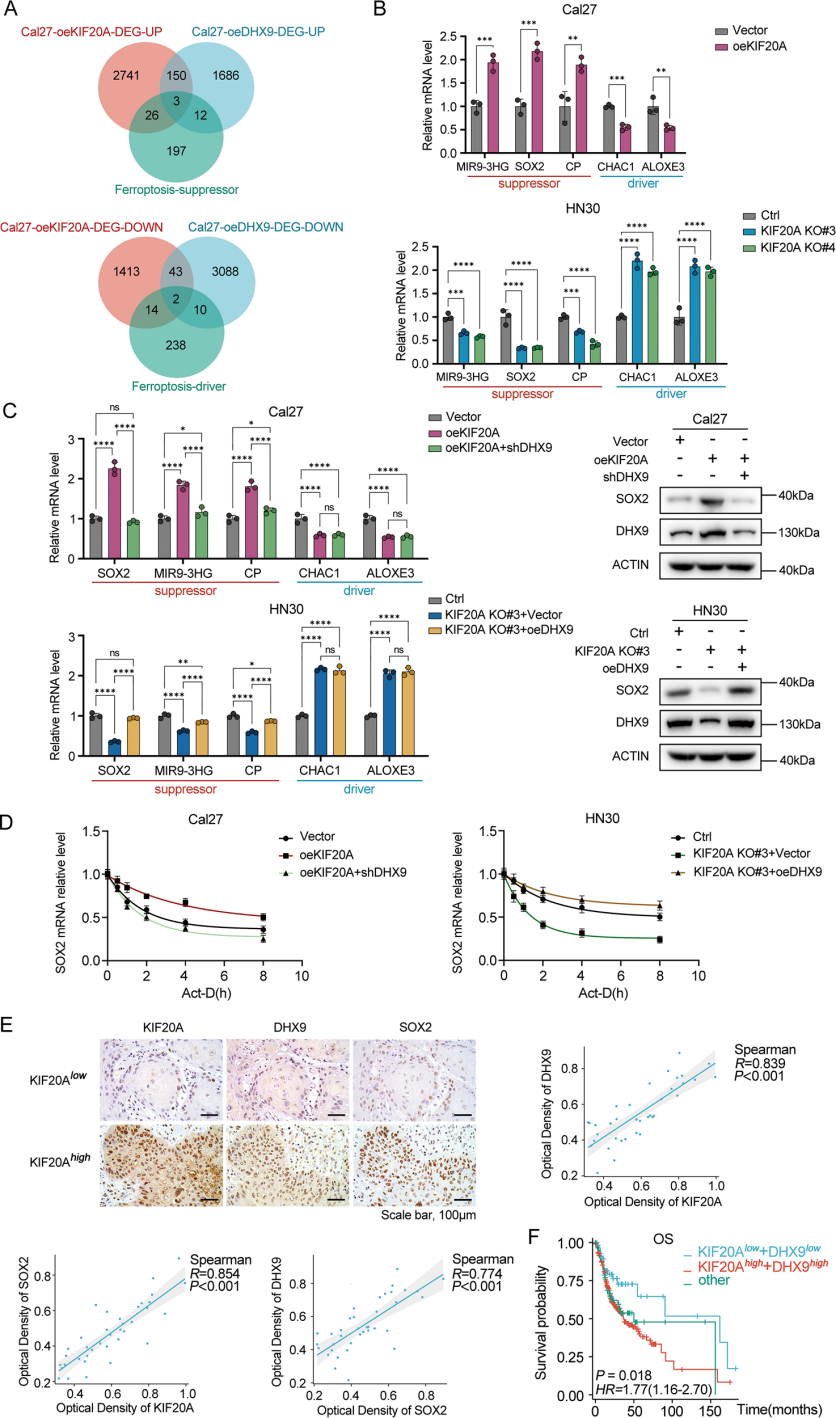
KIF20A regulates SOX2 expression in a DHX9-dependent manner. **A** Venn diagram showing the intersection of differentially expressed genes (DEGs) from RNA-seq analysis of KIF20A- or DHX9-overexpressing Cal27 cells with ferroptosis-regulating genes, including suppressors (CP, SOX2, MIR9-3HG) and drivers (CHAC1, ALOXE3). **B** RT-qPCR analysis of mRNA levels of CP, SOX2, MIR9-3HG, CHAC1, and ALOXE3 in stable KIF20A-overexpressing and knockout cell lines. **C** RT-qPCR and Western blot analysis of SOX2 expression levels following co-intervention of KIF20A and DHX9. **D** RT-qPCR analysis of SOX2 mRNA stability in stable KIF20A-overexpressing and knockout cell lines. **E** Representative immunohistochemical (IHC) staining of KIF20A, DHX9, and SOX2 in OSCC tissue samples with low or high KIF20A expression (*n* = 37 per-group). **F** Kaplan–Meier overall survival analysis of OSCC patients stratified by combined expression levels of KIF20A and DHX9. Patients were grouped into KIF20A^low^+DHX9^low^, KIF20A^high^+DHX9^high^, and other. Statistical significance is denoted by ****P < 0.0001, ***P < 0.001, **P < 0.01, *P < 0.05, ns not significant.

To elucidate the downstream targets regulated by KIF20A that are implicated in OSCC stemness and ferroptosis resistance, as well as to clarify the molecular significance of its interaction with DHX9, we performed RNA-seq on Cal27 cells overexpressing either KIF20A or DHX9. Differentially expressed genes (DEGs, overexpression vs. vector, |log_2_FC| ≥ 1, *P* < 0.05) were identified (Supplementary Fig. 8A), and the two DEG datasets were intersected (Supplementary Tables 2 and 3), yielding a subset of genes related to ferroptosis regulation (Fig. 7A).

Among the overlapping targets, genes such as CP [42], SOX2 [35], and MIR9-3HG [43] have previously been identified as ferroptosis suppressors, while CHAC1 [44] and ALOXE3 [45] were recognized as ferroptosis drivers. Follow-up RT-qPCR validation in four paired OSCC cell lines confirmed that CP, SOX2, MIR9-3HG, CHAC1, and ALOXE3 are transcriptionally regulated by KIF20A (Fig. 7B and Supplementary Fig. 8B). Notably, SOX2 garnered particular attention, as it is a master regulator of stemness and has also been shown to promote ferroptosis resistance [46].

Previous studies by Jiang et al. have demonstrated that DHX9, an RNA-binding protein, enhances SOX2 expression by maintaining mRNA stability [26]. To examine whether KIF20A-mediated regulation of SOX2 is dependent on DHX9, we performed co-intervention experiments by targeting both KIF20A and DHX9. Results from RT-qPCR and Western blot revealed that the KIF20A-induced expression of SOX2, MIR9-3HG, and CP was significantly attenuated upon DHX9 knockdown (Fig. 7C), indicating a DHX9-dependent mechanism.

Furthermore, mRNA stability assays confirmed that KIF20A promotes the stabilization of SOX2 mRNA in a DHX9-dependent manner (Fig. 7D). To validate these molecular findings at the tissue level, we performed IHC analysis of OSCC samples stratified by KIF20A expression. A significant positive correlation was observed among the protein levels of KIF20A, DHX9, and SOX2 (Fig. 7E), further supporting the existence of a KIF20A-DHX9-SOX2 regulatory axis in OSCC. Finally, Kaplan-Meier survival analysis based on TCGA-OSCC data revealed that patients with high expression of both KIF20A and SOX2 had significantly worse overall survival compared to those with low expression of both genes (Fig. 7F). These data collectively suggest that KIF20A promotes stemness and ferroptosis resistance in OSCC through DHX9-mediated stabilization of SOX2 mRNA, and that targeting the KIF20A-DHX9-SOX2 axis may represent a novel therapeutic strategy for OSCC.

### Targeting KIF20A represents a potential therapeutic strategy for treating OSCC

**Fig. 8:**
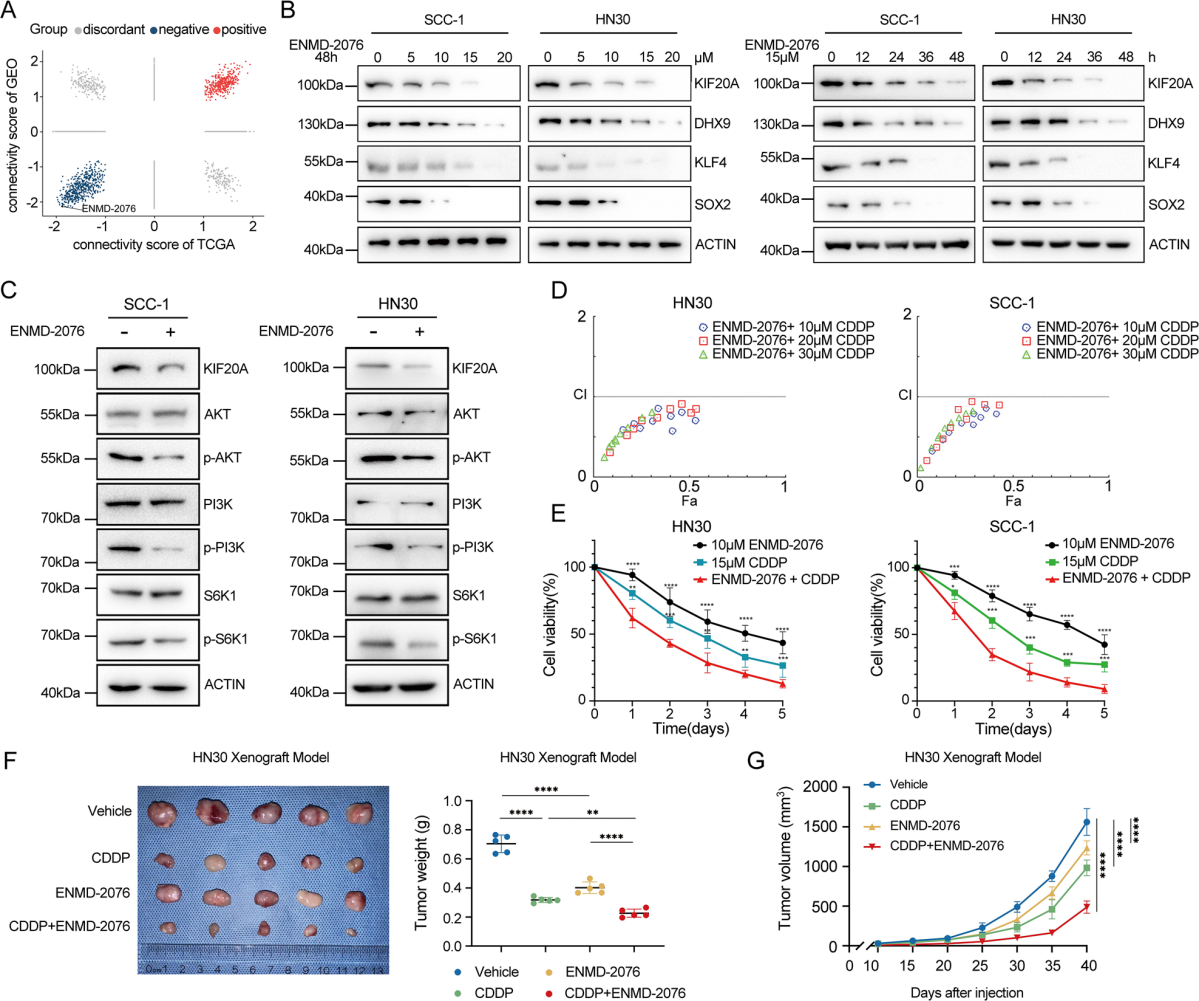
Inhibiting KIF20A represents a potential therapeutic strategy for treating OSCC. **A** Connectivity Map (CMap) analysis identified ENMD-2076 as a potential compound targeting KIF20A, with a significantly negative connectivity score in OSCC cell lines. **B** Western blot analysis of KIF20A, DHX9, KLF4, and SOX2 protein levels in HN30 and SCC-1 cells treated with ENMD-2076 in a time- and dose-dependent manner. **C** Western blot analysis of key components in the PI3K-AKT signaling pathway in HN30 and SCC-1 cells following ENMD-2076 treatment. **D** Quantification of the effects of ENMD-2076 and CDDP on the viability of HN30 and SCC-1 cells using the CCK-8 assay. CI values were calculated using CompuSyn software. **E** Cell viability assessment using the CCK-8 assay after time-dependent treatment of HN30 and SCC-1 cells with CDDP alone, ENMD-2076 alone, or a combination of both agents. Xenograft experiments showing tumor images and tumor weight statistics (**F**) as well as tumor growth curves (**G**) for HN30-derived tumors treated with CDDP, ENMD-2076, or their combination (*n* = 5 per group). Statistical significance is denoted by ****P < 0.0001, ***P < 0.001, **P < 0.01, *P < 0.05.

Building upon our earlier findings that KIF20A knockout suppresses OSCC malignancy, we further explored the therapeutic potential of targeting KIF20A. Using the Connectivity Map (CMap) platform in conjunction with TCGA and GEO expression data for OSCC, we identified ENMD-2076 as a promising candidate compound. In this study, ENMD-2076 was utilized as a gene silencing agent, serving to pharmacologically reduce KIF20A expression. Notably, ENMD-2076 exhibited a significantly negative connectivity score, indicating its potential to counteract KIF20A-associated transcriptional signatures in OSCC cell lines (Fig. 8A). ENMD-2076 is a novel, orally bioavailable multi-targeted kinase inhibitor with documented anti-tumor activity [47]. To assess its functional efficacy in OSCC, we determined the IC₅₀ values of ENMD-2076 in two KIF20A-high OSCC cell lines, HN30 and SCC-1, which were calculated to be 16.996 μM and 11.075 μM, respectively (supplementary Fig. 12A). Treatment with ENMD-2076 led to a significant time- and dose-dependent reduction in KIF20A, DHX9, KLF4, and SOX2 protein levels (Fig. 8B), suggesting that the compound effectively suppresses OSCC cancer stemness traits. Additionally, Western blot analysis revealed that ENMD-2076 inhibited the phosphorylation of key components in the PI3K-AKT signaling pathway, including AKT, PI3K, S6K1, and 4EBP1 (Fig. 8C), further correlating KIF20A suppression with inhibition of stemness-associated signaling. These results strongly support ENMD-2076 as a potential therapeutic agent for targeting KIF20A-driven malignancy in OSCC.

To investigate the potential synergistic effects of ENMD-2076 and cisplatin (CDDP) in OSCC treatment, we performed combination assays by treating cells with varying concentrations of ENMD-2076 (0, 3, 6, 9, 12, 15, 18, 21, 24 μM) and CDDP (10, 20, or 30 μM) for 48 h. Combination Index (CI) values were calculated using CompuSyn software, and all CI values were less than 1, indicating synergistic or at least additive interactions between the two agents in suppressing cell viability (Fig. 8D and Supplementary Tables 4 and 5). A five-day CCK-8 viability assay further corroborated these findings. The combined treatment produced a significantly greater anti-proliferative effect compared to either ENMD-2076 or CDDP alone under various dosage combinations (Fig. 8E), reinforcing the therapeutic potential of the dual-drug regimen in vitro.

To validate these findings in vivo, we established a xenograft model using HN30 cells. Equal numbers of cells were subcutaneously injected into 6-week-old female BALB/c nude mice. Beginning on day 10 post-injection, mice received treatments every 4 days: ENMD-2076 (200 mg/kg, oral gavage), CDDP (4 mg/kg, intraperitoneally), or a combination of both agents. On day 40, tumors were harvested and analyzed. The combination group exhibited significantly enhanced tumor growth inhibition compared to either monotherapy (Fig. 8F, G). Notably, no significant changes in mouse body weight were observed across all groups, indicating good tolerability of the treatment regimens (Supplementary Fig. 12B). These results underscore ENMD-2076 as a promising adjuvant therapeutic agent that enhances cisplatin efficacy in OSCC treatment. To further validate the in vivo mechanism, immunohistochemical analysis was performed on xenograft tumor sections. Consistent with the in vitro findings, xenografts from ENMD-2076–treated mice exhibited markedly reduced KIF20A, DHX9, and SOX2 staining compared with vehicle or CDDP groups, whereas the combination treatment produced the most pronounced decrease. These findings confirm that ENMD-2076 effectively suppresses the KIF20A–DHX9–SOX2 axis in vivo (Supplementary Fig. 13).

## Discussion

**Fig. 9:**
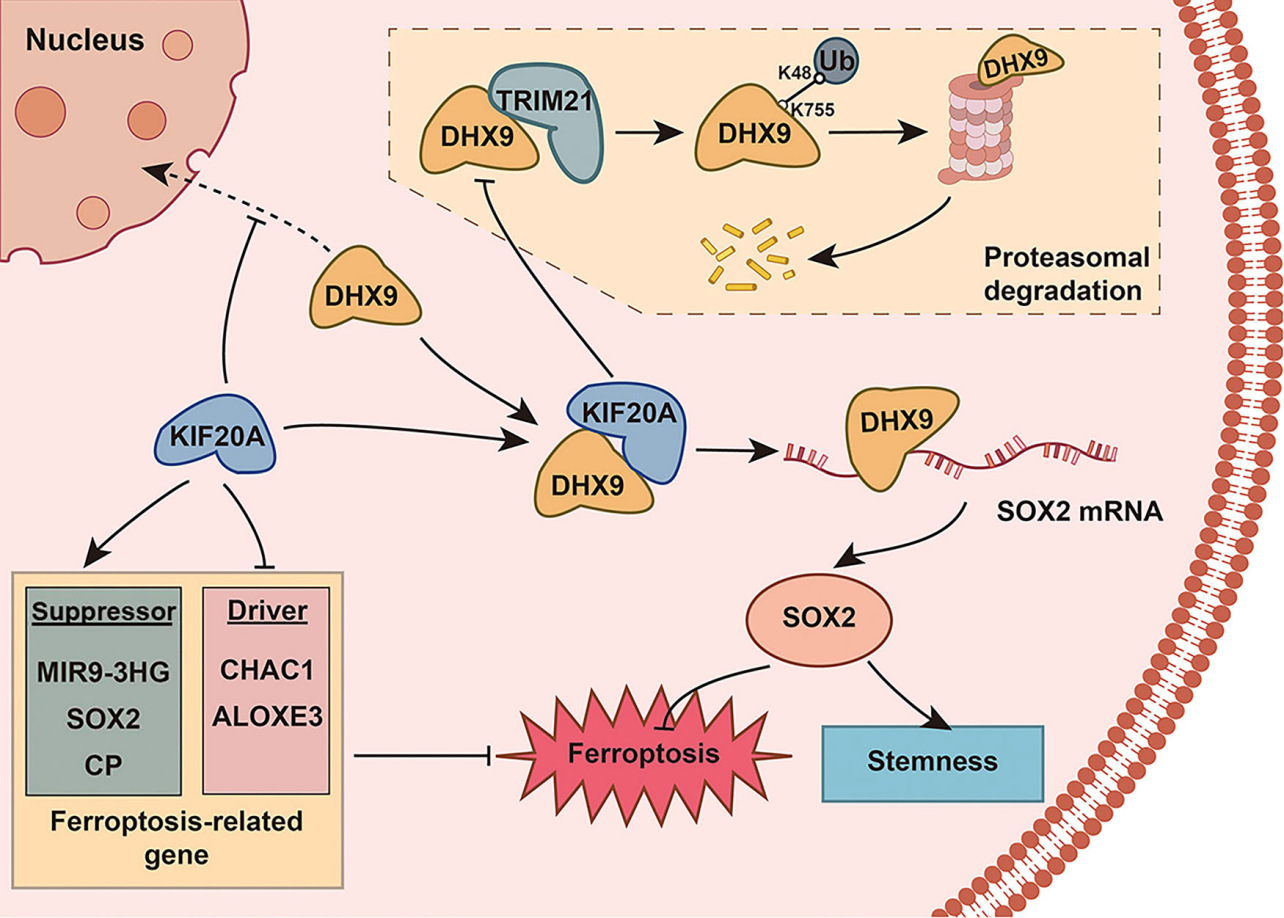
Graphical abstract. The KIF20A/DHX9/SOX2 axis regulates the stemness characteristics and ferroptosis of OSCC cells.

In this study, we report the aberrant overexpression of KIF20A in OSCC, promoting the malignant phenotype of the cancer. We identify KIF20A as a key positive regulator of OSCC aggressiveness, with its interaction through the KIF20A-DHX9-SOX2 axis activating the PI3K-AKT signaling pathway to enhance stemness and ferroptosis resistance. Furthermore, we report for the first time that KIF20A overexpression in OSCC contributes to the malignancy of the disease by stabilizing DHX9, which subsequently upregulates SOX2 expression (Fig. 9). Interestingly, ENMD-2076, a gene silencing agent targeting KIF20A, synergistically or at least additively enhances cisplatin (CDDP)-mediated suppression of OSCC cell proliferation. Our findings not only reveal the previously unexplored tumor-promoting function of KIF20A in OSCC but also underscore its potential as a therapeutic target for improving OSCC treatment outcomes.

In recent years, several studies have identified the role of KIF20A in the diagnosis and treatment of various malignancies. Specifically, KIF20A has been shown to be highly expressed and associated with poor prognosis in various malignancies, including glioma [48], prostate cancer [49], epithelial ovarian cancer [50], breast cancer [51], lung adenocarcinoma [10], colorectal cancer [52], and conjunctival melanoma [18]. Notably, although definitive studies on the role of KIF20A in OSCC are lacking, prior investigations have highlighted the oncogenic significance of other kinesin family members in this malignancy. Overexpression of KIF4A, KIF11, and KIF14 has been linked to enhanced proliferation, metastasis, and unfavorable survival [53–55], while KIF20B and KIF2A have been shown to promote tumor growth and dissemination [56, 57]. Similarly, high KIF5B expression correlates with adverse clinicopathological features, increased recurrence risk, and reduced overall survival [58]. Collectively, these findings underscore the oncogenic roles of multiple KIF proteins in OSCC and highlight their dual relevance as prognostic biomarkers and therapeutic targets. Against this background, the present study investigated the expression and functional significance of KIF20A in OSCC, revealing its elevated expression and strong association with poor patient prognosis.

CSCs are a small subpopulation of tumor cells with self-renewal and differentiation capabilities, driving tumorigenesis, metastasis, and resistance to therapy. The persistence of CSCs in OSCC is linked to poor prognosis and therapeutic failure, which emphasizes the need to target stemness for effective treatment [59–61]. Ferroptosis is a form of programmed cell death mediated by lipid peroxidation, and CSCs have been shown to exhibit greater resistance to ferroptosis due to their enhanced antioxidant system [6, 35]. Our study identifies a critical role for KIF20A in promoting stemness and ferroptosis resistance in OSCC. In line with these findings, recent evidence has demonstrated that SOX2 directly contributes to ferroptosis resistance through transcriptional regulation of antioxidant genes. Specifically, SOX2 has been shown to bind to the promoter of SLC7A11, thereby enhancing its transcription and conferring ferroptosis resistance in glioblastoma and lung cancer cells [35, 62]. Similar effects on the GPX4 axis have also been suggested, further underscoring SOX2 as a pivotal transcriptional regulator of ferroptosis susceptibility. Within the framework of our study, we identified a novel KIF20A–DHX9–SOX2 axis, wherein KIF20A stabilizes SOX2 mRNA expression via DHX9, thereby maintaining high SOX2 levels that could, in turn, activate ferroptosis-protective programs. This axis thus not only reinforces the stemness-promoting function of SOX2 but also establishes a plausible link between KIF20A signaling and ferroptosis resistance.

By further exploring this regulatory mechanism through CoIP/MS, we discovered for the first time that KIF20A can bind to DHX9. Previous studies have shown that DHX9 is upregulated in various tumors, including hepatocellular carcinoma [63, 64], renal carcinoma [65], colorectal carcinoma [66], and ovarian carcinoma [67], and plays a crucial role in tumor development. Interestingly, we found that KIF20A affects the ratio of cytoplasmic/cytosolic distribution of DHX9 and also regulates DHX9 expression at the protein level, with no significant effect on mRNA expression levels. DYNLRB2-AS1 has been reported to regulate DHX9 expression by inhibiting DHX9 ubiquitination [68], while KIF20A can competitively inhibit FBXW7-mediated c-Myc ubiquitination and degradation [21]. This mechanism may resemble the way KIF20A regulates DHX9 expression at the protein level. As expected, we found that the E3 ligase TRIM21 is a common interacting protein of DHX9 and KIF20A. TRIM21 is a highly conserved E3 ligase [69–71]. TGM2 has been reported to block TRIM21-mediated STAT1 degradation by preventing its interaction with STAT1 [72]. Our study also revealed that KIF20A can competitively bind DHX9 with TRIM21 and interfere with TRIM21-mediated DHX9 degradation via a K48-linked polyubiquitin chain. We further designed and conducted rescue experiments, the results of which were consistent with the above findings and demonstrated that DHX9 serves as an essential mediator of KIF20A-driven oncogenic activity in OSCC. Functional complementation using full-length DHX9 effectively restored the impaired proliferative and stem-like phenotypes caused by KIF20A depletion, underscoring the causal role of DHX9 within this regulatory axis. While systematic truncation of DHX9 domains could provide additional structural insights into the specific regions responsible for KIF20A binding, such detailed mapping was not pursued in this study. Future work will therefore focus on fine-scale domain mapping and structural characterization to delineate the minimal interface required for the KIF20A–DHX9 interaction. These efforts will deepen the mechanistic understanding of how this axis contributes to tumor progression and may inform the development of targeted strategies to disrupt this interaction therapeutically.

To investigate the mechanism by which DHX9 affects KIF20A-regulated OSCC in terms of stemness and ferroptosis, we utilized RNA-seq to identify SOX2 as a downstream gene co-regulated by KIF20A and DHX9. SOX2 is reported to be not only a key gene in stemness regulation but also to promote ferroptosis resistance [35, 73]. By co-intervening in KIF20A and DHX9 expression, we found that KIF20A relies on DHX9 to regulate SOX2 expression and maintain SOX2 mRNA stability. Together, these findings suggest that KIF20A promotes OSCC stemness and resistance to ferroptosis through the DHX9-SOX2 axis.

Small-molecule inhibitors are crucial in cancer therapy, targeting specific proteins to regulate key processes such as tumor cell proliferation, stemness, and ferroptosis. These inhibitors offer an effective and convenient approach, particularly for patients with specific genetic alterations or distinct characteristics [74, 75]. In this study, we identified the small-molecule inhibitor ENMD-2076 as a novel agent that downregulates KIF20A expression and effectively inhibits the malignant progression of OSCC cells by targeting the KIF20A-DHX9-SOX2 pathway. ENMD-2076, an Aurora-A kinase inhibitor with anti-angiogenic properties, has demonstrated activity in breast cancer, fibrolamellar carcinoma and hematologic malignancies [76–78]. Furthermore, our study expanded the role of ENMD-2076 in OSCC; in addition, a potentiating effect with cisplatin was observed, with the combination treatment markedly enhancing the suppression of OSCC cells exhibiting high KIF20A expression. Notably, ENMD-2076 has been reported to exhibit activity against platinum-resistant ovarian cancer [79]. ENMD-2076 clearly inhibits OSCC cell proliferation and KIF20A expression, suggesting its potential as a pharmacological tool for investigating KIF20A-related pathways. However, as ENMD-2076 is a multi-kinase inhibitor with known activity against Aurora A and other kinases, the possibility of off-target effects cannot be ruled out. To substantiate the involvement of KIF20A further, future studies should combine pharmacological and genetic approaches. For example, they could evaluate the efficacy of ENMD-2076 in KIF20A-silenced models or employ next-generation compounds with improved selectivity. Such investigations will help to determine the extent to which the therapeutic effects of ENMD-2076 are directly attributable to KIF20A inhibition versus the modulation of the broader kinase network. Cisplatin, a first-generation platinum-based compound, is a first-line, non-specific cytotoxic chemotherapeutic agent widely used in the treatment of solid tumors, including OSCC [80]. However, cisplatin resistance has been reported to significantly compromise the efficacy of chemotherapy in OSCC, highlighting the potential value of combination therapies to overcome this challenge [79, 81]. Our study further validated the synergistic effect of ENMD-2076 and cisplatin, suggesting a promising strategy to overcome cisplatin resistance. Therefore, the discovery of specific small-molecule inhibitors targeting KIF20A holds promise for the development of novel treatments for OSCC.

In conclusion, this study elucidates the role of KIF20A in regulating stemness and ferroptosis during OSCC pathogenesis and identifies, for the first time, the molecular mechanism through which the KIF20A/DHX9/SOX2 axis orchestrates OSCC progression. Furthermore, this study demonstrates the profound efficacy of ENMD-2076 with cisplatin in suppressing the proliferation of KIF20A-high-expressing OSCC cells in vitro and in vivo. These findings establish KIF20A as a promising therapeutic target for OSCC and provide a robust theoretical and experimental foundation for targeted drug development. However, this study also has several limitations. First, it did not investigate the effect of potential KIF20A inhibitors on ferroptosis in OSCC. Second, further investigation into the specific mechanisms by which ENMD-2076 affects KIF20A expression is needed. Finally, further exploration of how KIF20A influences the cytoplasmic/nuclear distribution ratio of DHX9 is necessary. These areas will be explored in future studies, and we encourage collaboration with scholars in related fields to conduct experiments that will further our understanding of this area.

## Materials and methods

### Cell culture and treatment

Human immortalized keratinocytes (HaCat) and human OSCC cell lines, including Cal27, HN30, HN6, and SCC-1, were obtained from the Type Culture Collection of the Chinese Academy of Sciences (Shanghai, China). The cells were cultured in DMEM (Gibco, Carlsbad, CA, USA), supplemented with 10% (v/v) fetal bovine serum (FBS; Gibco, Carlsbad, CA, USA) and 1% penicillin/streptomycin. Human embryonic kidney 293T cells, gifted from the Cancer Research Institute of Central South University, were cultured and incubated in DMEM containing 10% fetal bovine serum. All cells were maintained at 37 °C in a humidified atmosphere containing 5% CO₂. To suppress RNA or protein synthesis, cells were treated with actinomycin D (Act D, CST, MA, USA) or cycloheximide (CHX, Selleck, TX, USA) for the specified durations. Additionally, MG132 (Selleck, TX, USA) was employed to inhibit protein degradation through the proteasome pathway. All cell lines were authenticated by short tandem repeat (STR) profiling prior to experimentation. All cell lines were confirmed to be free of mycoplasma contamination before use.

### RNA extraction and real-time quantitative PCR (RT-qPCR)

Total RNA was extracted using Trizol reagent (R401-01, Vazyme, Nanjing, China), and complementary DNA was synthesized using HiScript® II Q RT SuperMix for qPCR (+gDNA wiper) (R223-01, Vazyme, Nanjing, China) following the manufacturer’s protocol. RT-qPCR was conducted on the Bio-Rad CFX Connect Real-Time PCR System utilizing MonAmp™ ChemoHS qPCR Mix (MQ00401S, Monad, Shanghai, China). ACTIN was employed as an internal control for normalization. All experiments were performed with at least three independent biological replicates, each repeated in triplicate. The sequences of all primers for RT-qPCR are provided in Supplementary Table 1.

### Western blot

Protein samples extracted from the cells were separated using SDS-PAGE gel and subsequently transferred to PVDF membrane for primary antibody hybridization. For Western blot analysis, the following antibodies were utilized: ACTIN (#AF7018, Affinity, 1:4000), KIF20A (#sc-374508, Santa Cruz, 1:500), DHX9 (#17721-1-AP, Proteintech, 1:5000), SOX2 (#A19118, abclonal, 1:1000), KLF4 (#A13673, abclonal, 1:2000), c-MYC (#10828-1-AP, Proteintech, 1:10000), HA (#51064-2-AP, Proteintech, 1:5000), Flag (#20543-1-AP, Proteintech, 1:20,000), TRIM21(#12108-1-AP, Proteintech, 1:1000), p-PI3K (#AF3242, Affinity, 1:1000), PI3K (#60225-1-Ig, Proteintech, 1:1000), p-AKT (#66444-1-Ig, Proteintech, 1:5000), AKT (#YM3618, ImmunoWay, 1:1000), p-4EBP1 (#R22929, Zenbio, 1:1000), 4EBP1 (#R24197, Zenbio, 1:1000), p-S6K1 (#310310, Zenbio, 1:1000), S6K1 (#380469, Zenbio, 1:1000), ubiquitin (#10201-2-AP, Proteintech, 1:4000), α-Tubulin (#AF7010, Affinity, 1:10,000), Histone H3 (#17168-1-AP, Proteintech, 1:6000), goat anti-rabbit secondary antibody (#S0001, Affinity, 1:6000), and goat anti-mouse secondary antibody (#S0002, Affinity, 1:6000). The resulting protein bands were visualized using a luminescence detection kit (Vazyme, China). All experiments were performed with at least three independent biological replicates, each repeated in triplicate. Finally, the images were captured using a chemiluminescence imaging system (Tanon-5200, Shanghai, China). Uncropped images of all Western blot membranes used in this study have been compiled and are provided as a supplementary source data file (“Uncropped western blots.pdf”).

### H&E staining

Paraffin tissue sections were deparaffinized with xylene (twice for 10 min each) and then placed in anhydrous ethanol (twice for 10 min each), 90% ethanol (5 min), 80% ethanol (5 min), 70% ethanol (3 min) and the tissues were washed with primary water. Subsequently, the tissues were sequentially stained with hematoxylin staining solution (3 min), differentiation solution (1 s), eosin solution (15 s), 95% ethanol (5 s), anhydrous ethanol (twice for 1 min each) and xylene (twice for 2 min each). Finally, the slices were sealed with neutral resin and observed by microscope (Carl Zeiss, Suzhou, China).

### Immunohistochemistry and Immunofluorescence

Immunohistochemical staining was conducted and analyzed according to described previously [82]. For immunofluorescence staining, cells were seeded on glass coverslips in 12-well platesand then fixed by 4% paraformaldehyde fixation. Cells were subsequently incubated with PBS containing 0.25% Triton X-100 and 5% BSA for 1 h. Then incubated them with KIF20A (#sc-374508, Santa Cruz, 1:500), DHX9 (#17721-1-AP, Proteintech, 1:5000) overnight at 4 °C in a humidified chamber. Slides were then incubated with fluorescein-conjugated secondary antibodies for 1 h at room temperature in the dark. Nuclei were counterstained with 4′, 6-diamidino-2-phenylindole (DAPI). We used confocal microscopy (Leica Microsystems CMS GmbH, Mannheim, Germany) for imaging. Pearson correlation coefficients were determined for molecular colocalization in confocal microscopy images using Coloc 2 of Fiji.

### Cell proliferation assay

After seeding the cells in 96-well plates for indicated days, cell proliferation was assessed using a Cell Counting Kit-8 (CCK-8, #C0005, TargetMol). The CCK-8 solution was diluted 1:10 in medium and incubated with the cells at 37 °C for 2 h. Subsequently, the OD value was measured at 450 nm using a microplate reader (Synergy 2, Bio-Tek, USA). OSCC cells were seeded in six-well plates and cultured for 14 days to perform a colony formation assay. Subsequently, colony formation analysis was conducted using ImageJ software. All experiments were performed with at least three independent biological replicates, each repeated in triplicate.

### Coimmunoprecipitation (Co-IP) assay and LC-MS/MS analysis

Cells were lysed using IP lysis buffer supplemented with a protease inhibitor cocktail. The extracted proteins were incubated overnight at 4 °C with a primary antibody, after which Protein G Plus Magnetic Beads (Beyotime P2106) were added for rotational incubation for 1 h. The resulting immunoprecipitated complexes were subsequently washed three times with cold IP buffer. The purified proteins were then utilized for Western blot analysis and sent to Shanghai OE Biotech Co., Ltd (Shanghai, China) for LC-MS/MS protein identification. For proteomic data, *P* values were adjusted using the Benjamini–Hochberg procedure to correct for multiple comparisons.

### RNA sequencing and data analysis

Total RNA of the target cells was collected and sent to Majorbio Biopharmaceutical Technology (Shanghai, China) for RNA expression profiling. DEGs between treated and control groups were screened using the “edgeR” R package (|log_2_FC| > 2, *P* < 0.05). DEGs were analyzed with the Kyoto Encyclopedia of Genes and Genomes (KEGG) to investigate KIF20A-related pathways. Differential expression analysis of RNA-seq data was performed using the DESeq2 package, with multiple testing controlled by the false discovery rate (FDR).

### Sphere formation assay

The cells were resuspended in sphere formation medium (DMEM-F12 + 2% B27 supplement + 20 ng/mL bFGF + 20 ng/mL EGF supplement) and inoculated in ultralow attachment 12-well plates. The size and number of tumor spheres formed were counted according to the Spheres with diameters ≥50 μm in the optical microscope. All experiments were performed with at least three independent biological replicates, each repeated in triplicate.

### Transwell assay

Transwell assays were performed as previously described [82]. Briefly, for the migration assay, 5 × 10^4^ cells were plated in the top chamber of each insert (200 μL/well) using serum-free medium, while the lower chamber contained 800 μL of medium supplemented with 10% FBS as a chemoattractant. For the invasion assay, the upper chambers were previously coated with extracellular matrix gel (082706, ABW, China). After incubation for 24–48 h, three random fields were selected, and the migration and invading capability of the tumor cells was analyzed using ImageJ software. All experiments were performed with at least three independent biological replicates, each repeated in triplicate.

### Flow cytometry

Cells in the logarithmic growth phase were processed into a single-cell suspension centrifuged for 5 min, and this procedure was repeated twice to discard the supernatant. Subsequently, 1 × 10^6^ cells were resuspended in 100 μL of PBS supplemented with 5% BSA and CD133 primary antibody (#A25678, ABclonal, 1:100), and incubated for 30 min at 4 °C in the dark. Following two rounds of centrifugation in PBS and removal of the supernatant, the cells were resuspended in 100 μL of BSA-diluted fluorescent marker. The cells were then incubated with 100 μL of Fluor488-conjugated Goat Anti-Mouse IgG(H + L) secondary antibody (#S0017, Affinity, 1:100) for 20 min at 4 °C in the dark, centrifuged twice with PBS, and the supernatant was discarded. The cells were finally resuspended in 100 μL of 1× PBS with 5 μL of PI stain added and incubated for 2 min at 4 °C in the dark. An additional 400 μL of 1× PBS was added before analysis via BD FACS Lyrics flow cytometer. All experiments were performed with at least three independent biological replicates, each repeated in triplicate. Data were analyzed using FlowJo software (v10.8.1, FlowJo).

### Lipid peroxidation was evaluated using Liperfluo staining

Cells were incubated with Liperfluo (Dojindo Molecular Technologies, Shanghai, China) for 30 min at 37 °C, collected by trypsinization, and subsequently analyzed immediately using flow cytometer (BD FACS Lyrics) for excitation.

### MDA assay

Cells were cultured in 6-well plates and collected individually 24 h later. After resuspending the cells in PBS supplemented with PMSF, ultrasonic disruption was carried out using an ultrasonic disruptor. The sonicated cells were then quantified using the BCA Protein Detection Kit. Levels of malondialdehyde (MDA) were measured using a cell malondialdehyde detection kit, following the manufacturer’s instructions.

### Cell ferrous iron assay

The concentration of ferrous iron in the cells was assessed using the Cell Ferrous Iron Colorimetric Assay Kit (E-BC-K881-M, Elabscience). The experiment followed the provided instructions, and luminescence values were measured with an enzyme-labeled detection instrument. The levels of ferrous iron were calculated using a standard curve and a provided formula according to the product instructions.

### mRNA stability assay

The target cells were treated with 5 μg/mL of Actinomycin D (Act-D, M4881, AbMole) for 0, 0.5, 1, 2, 4, and 8 h. Total cell RNA was collected and extracted, and the mRNA expression of the target genes was detected by RT-qPCR assay.

### Cell counting Kit-8 (CCK8) assay and combination index (CI)

Cells were seeded into 96-well plates at 2000 cells per well. After 24 h of seeding, the cells were treated with escalating concentrations of sorafenib for 48 h. Ten microliters of CCK-8 reagent (Vazyme, China) were added to each well of the cells and then incubated for 2 h at 37 °C. Absorbance at 450 nm was measured with a Synergy 2 microplate reader (BioTek, USA). Subsequently, cell viability was visualized using GraphPad Prism (v 9.1.0) (GraphPad Software, San Diego, CA, USA).

Cells were seeded at 1500 cells per well in a 96-well plate and incubated overnight. The HN30 and SCC-1 cells were treated with ENMD-2076 (E408042, Aladdin), CDDP (HY-17394, MCE), or a control vehicle for 48 h, both as single agents and in combination. Cell viability was assessed using a CCK-8 assay, and CI was generated with CompuSyn software (Cambridge, UK) to quantify the effects of drug combinations. The drugs’ synergistic, additive, and antagonistic effects were defined by CI values of less than 1, equal to 1, and greater than 1, respectively.

### Connectivity map

Differential expression analyses were performed by stratifying tumors into KIF20A-high and KIF20A-low groups in the TCGA-OSCC and GSE41613 datasets using edgeR (|log_2_FC| ≥ 1, FDR < 0.05). Subsequently, the top 150 upregulated and top 150 downregulated genes were then submitted to the Connectivity Map (CMap) platform (https://clue.io/) as query signatures, to identify compounds that inversely correlated with KIF20A. For each compound, enrichment scores were calculated by comparing its perturbational expression profile with the query signature and standardized into a connectivity score(τ). Compounds with strongly negative connectivity (*τ* < 0), indicative of reversal of the KIF20A-associated transcriptional program, were prioritized.

### In vitro limiting dilution analysis (LDA)

For the in vitro LDA, single-cell suspensions were seeded at varying densities (0, 20, 40, 60, 80, or 100 cells per well) into 96-well plates pre-coated with poly (2-hydroxyethyl methacrylate) (poly-HEMA; Sigma-Aldrich) to prevent cell adhesion. Cells were cultured in sphere-forming medium under standard conditions. Following an incubation period of 14 days, wells containing tumor spheres were scored. The data on positive wells across the dilution series were subsequently analyzed using the ELDA software (http://bioinf.wehi.edu.au/software/elda/) to determine the frequency of sphere-forming units.

### Nuclear and cytoplasmic protein fractionation

Nuclear and Cytoplasmic Protein Extraction Kit (P0027, Beyotime) was used to separate cytoplasm and nuclear protein according to the instruction. Under low osmotic pressure, the cells expand sufficiently, then the cell membrane was destroyed, and cytoplasmic proteins were released. The nucleus was then precipitated by centrifugation. Lastly, nuclear protein was extracted by high-salt nuclear protein extraction reagent.

### Animal models

Female BALB/c mice (4 weeks old) were obtained from Vital River Laboratory Animals Ltd (Beijing China). Animals were randomly divided into groups (*n* = 5 per group). KIF20A-overexpressing Cal27 cells or KIF20A-knockout HN30 cells, along with their corresponding control cell suspensions, were subcutaneously injected (100 µL, 2 × 10^6^ cells/mouse) into the subcutaneous space of nude mouse. Body weight was monitored every 3 days, and the short and long diameters of the tumors were measured starting on day 10 post-inoculation. After the tumor appeared, the mice were treated as follow: Ferrostatin-1 (2 mg/kg) was injected intraperitoneally every 4 days, ENMD-2076 (200 mg/kg) was administered orally via gavage every 4 days, Erastin (20 mg/kg) was injected intraperitoneally every 4 days, and CDDP (4 mg/kg) was injected intraperitoneally every 4 days. Monitoring continued until day 40 post-inoculation, at which point the nude mice were euthanized. Tumors were excised, photographed, and weighed. Tumor volume was calculated using the formula: tumor volume (mm³) = 0.5 × width² × length.

### Bioinformatic analysis

The specificity and sensitivity of KIF20A were assessed via receiver operating characteristic (ROC) curves, and the area under the curve (AUC) was quantified using the pROC R package. Combining survival time, survival status, and gene expression data, the R package “survival” was used to assess the prognostic significance of genes. Gene Set Enrichment Analysis is supported by the Broad Institute website (http://www.broadinstitute.org/gsea/index.jsp) and includes versions compatible with Java, R or Gene Pattern. UbiBrowser 1.0 (http://ubibrowser.ncpsb.org/) is an integrated bioinformatics platform that can be used to predict proteome-wide human E3-substrate networks based on naïve Bayesian networks. It currently contains 1295 literature-reported E3-substrate interactions and 8255 predicted E3-substrate interactions. We used it to predict the potential E3 ubiquitin ligase of DHX9.

### Statistical analysis

Experimental data were analyzed using GraphPad Prism 9.0 and are presented as mean ± SD. The normality of data distribution was confirmed using the Shapiro-Wilk test. All experiments were performed with at least three independent biological replicates, each repeated in triplicate. Statistical significance between two groups was determined using a two-tailed Student’s *t* test, while multiple group comparisons were assessed by one-way ANOVA followed by Tukey’s post hoc test. Homogeneity of variance was verified using Levene’s test, and *P* < 0.05 was considered statistically significant.

## Supplementary information

Quantitative statistics of Western blots

Supplementary information

Uncropped western blots

## Data Availability

The data presented in this study are openly available in TCGA database (https://portal.gdc.cancer.gov/), GEO database (https://www.ncbi.nlm.nih.gov/geo/), Metascape database (https://metascape.org/gp/), Ubibrowser 1.0 database (http://ubibrowser.bio-it.cn/ubibrowser/).
