# Supplementary figures and images for: KIF20A inhibits TRIM21-dependent ubiquitination of DHX9 to boost SOX2 stability, enhancing OSCC stemness and ferroptosis resistance
Source: Cell Death Dis. 2026 Feb 11;17(1):218. doi: 10.1038/s41419-026-08467-w (PMC12920667; doi:10.1038/s41419-026-08467-w)

**A**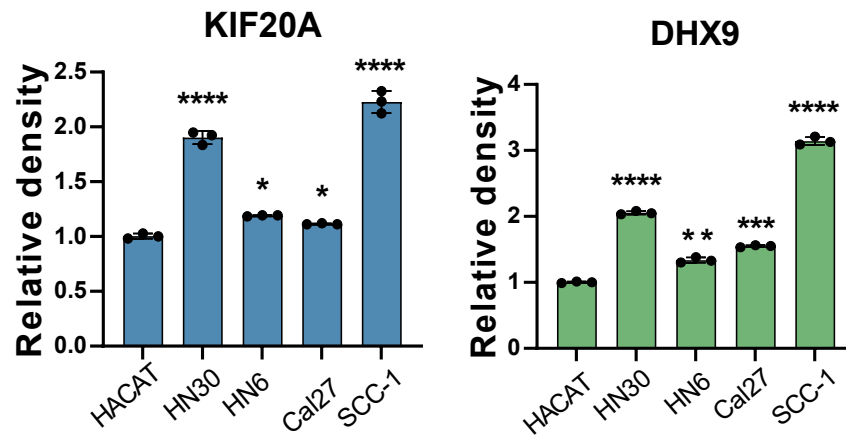**B**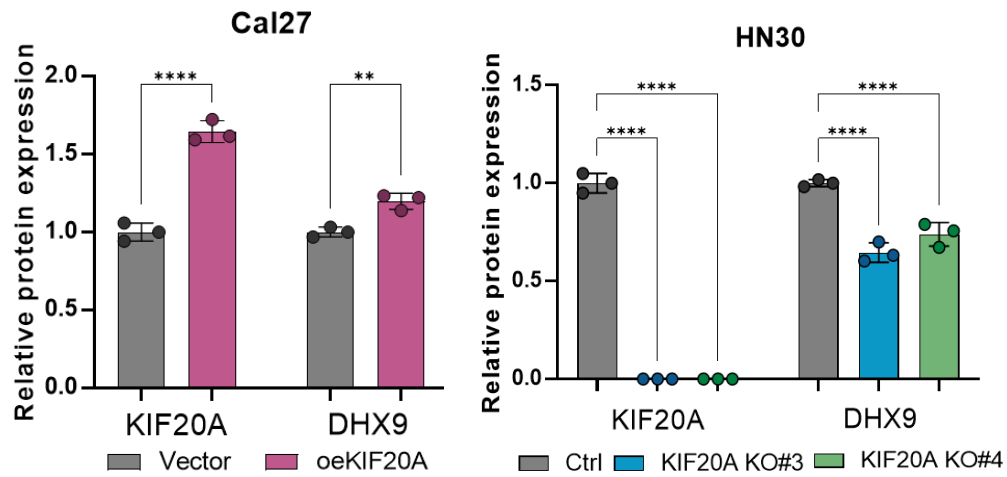**C**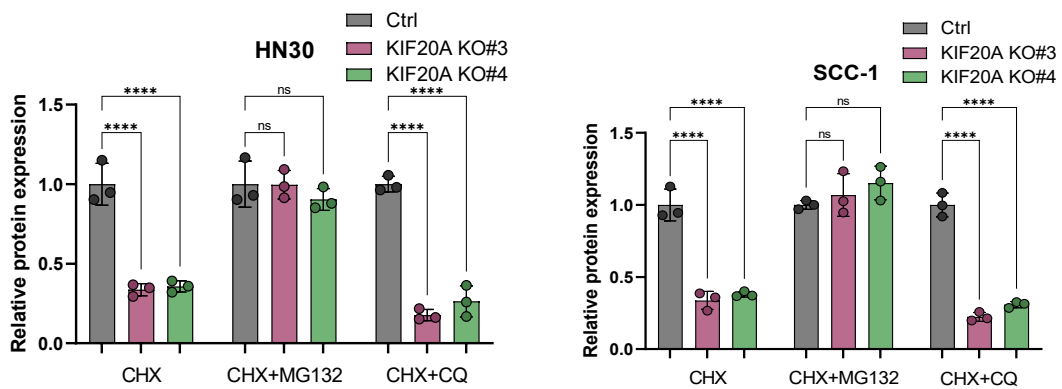

**D**

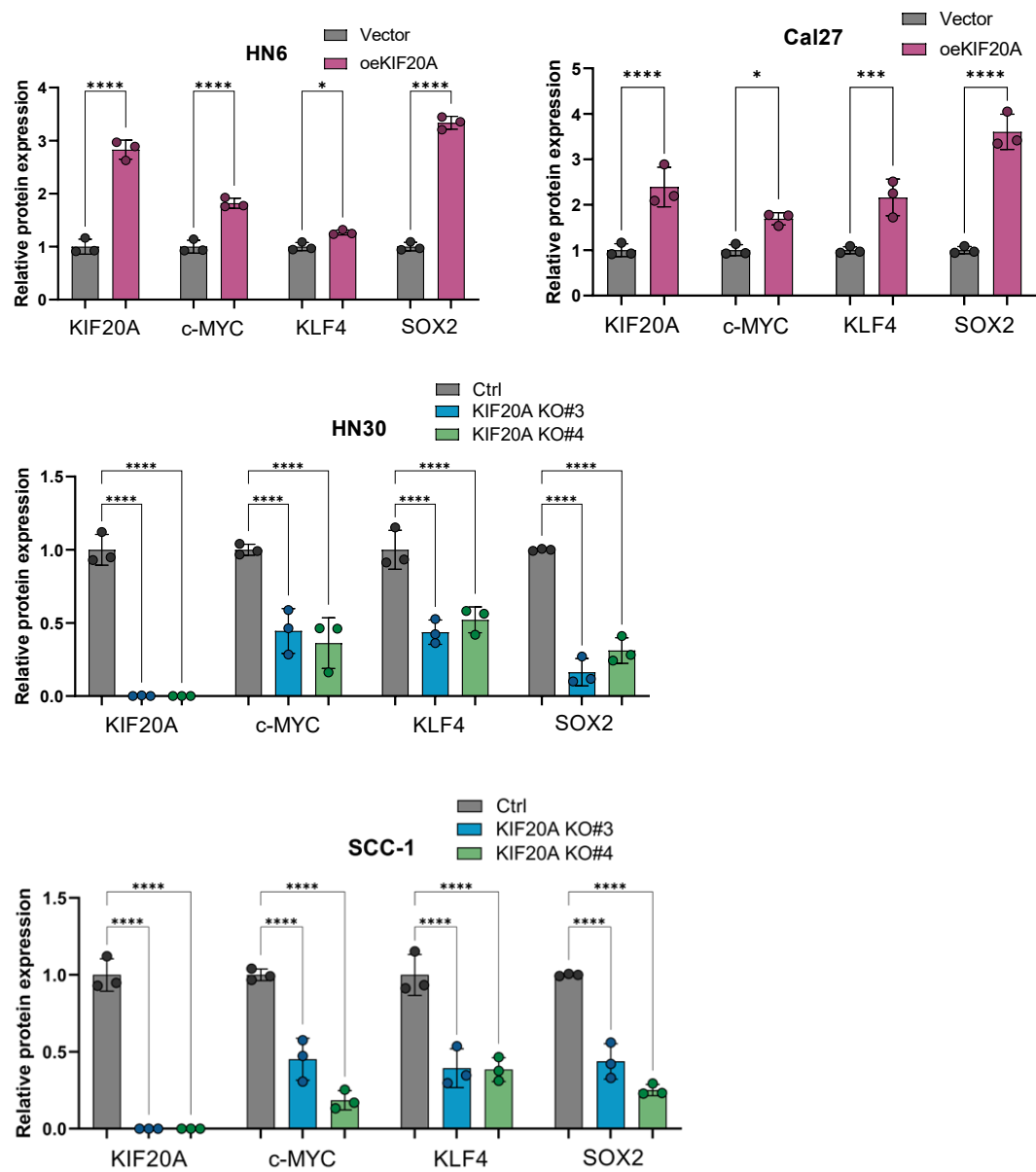

**E**

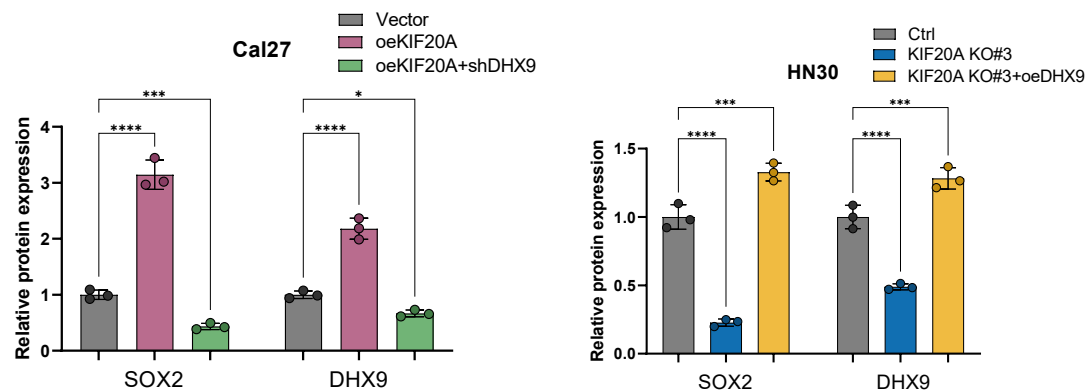

**F**

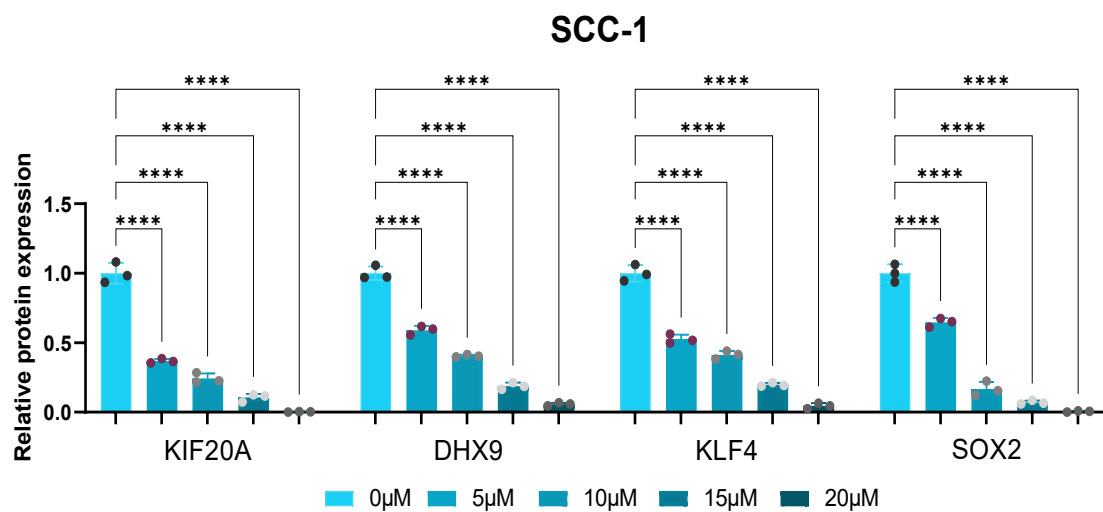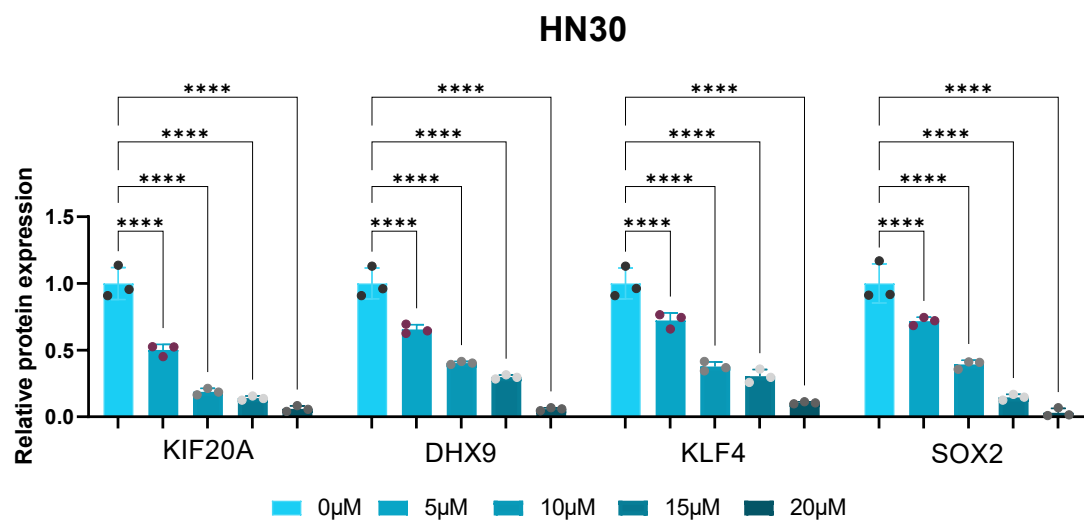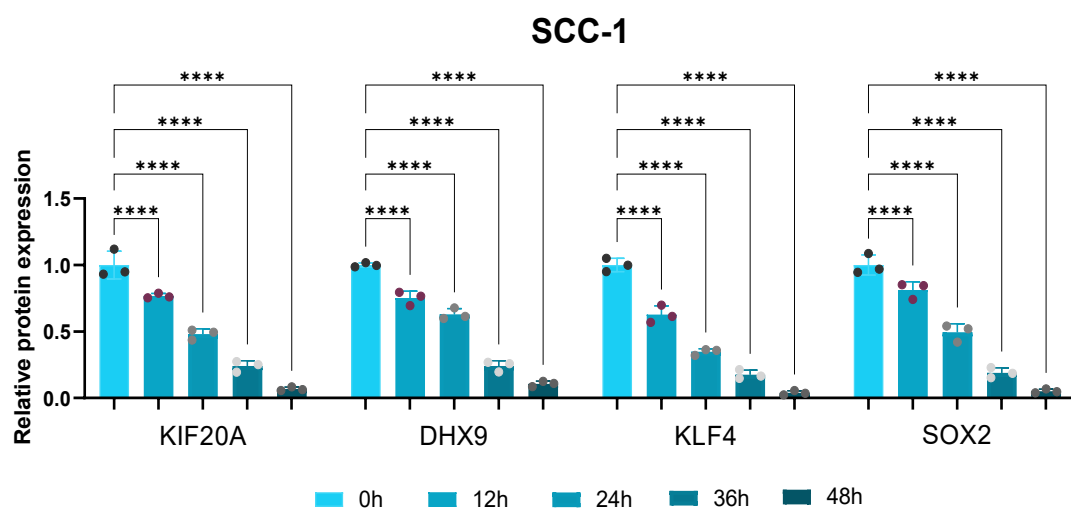



Figure7C. **(F)** Quantitative statistics of Figure8B. **(G)** Quantitative statistics of Figure8C.

Supplement: Supplementary file 1 — Quantitative statistics of Western blots [file 41419_2026_8467_MOESM1_ESM.pdf]
