# Supplementary material for: KIF20A inhibits TRIM21-dependent ubiquitination of DHX9 to boost SOX2 stability, enhancing OSCC stemness and ferroptosis resistance
Source: Cell Death Dis. 2026 Feb 11;17(1):218. doi: 10.1038/s41419-026-08467-w (PMC12920667; doi:10.1038/s41419-026-08467-w)
Supplement: Supplementary file 2 — Supplementary information [file 41419_2026_8467_MOESM2_ESM.docx]

**Supplementary information for**

**KIF20A Inhibits TRIM21-Dependent Ubiquitination of DHX9 to Boost SOX2 Stability, Enhancing OSCC Stemness and Ferroptosis Resistance**

Ziyun Zhang *et al.*

Corresponding author: Li Cong, congli@hunnu.edu.cn

Yiqun Jiang(lead contact), jiangyiqun@hunnu.edu.cn


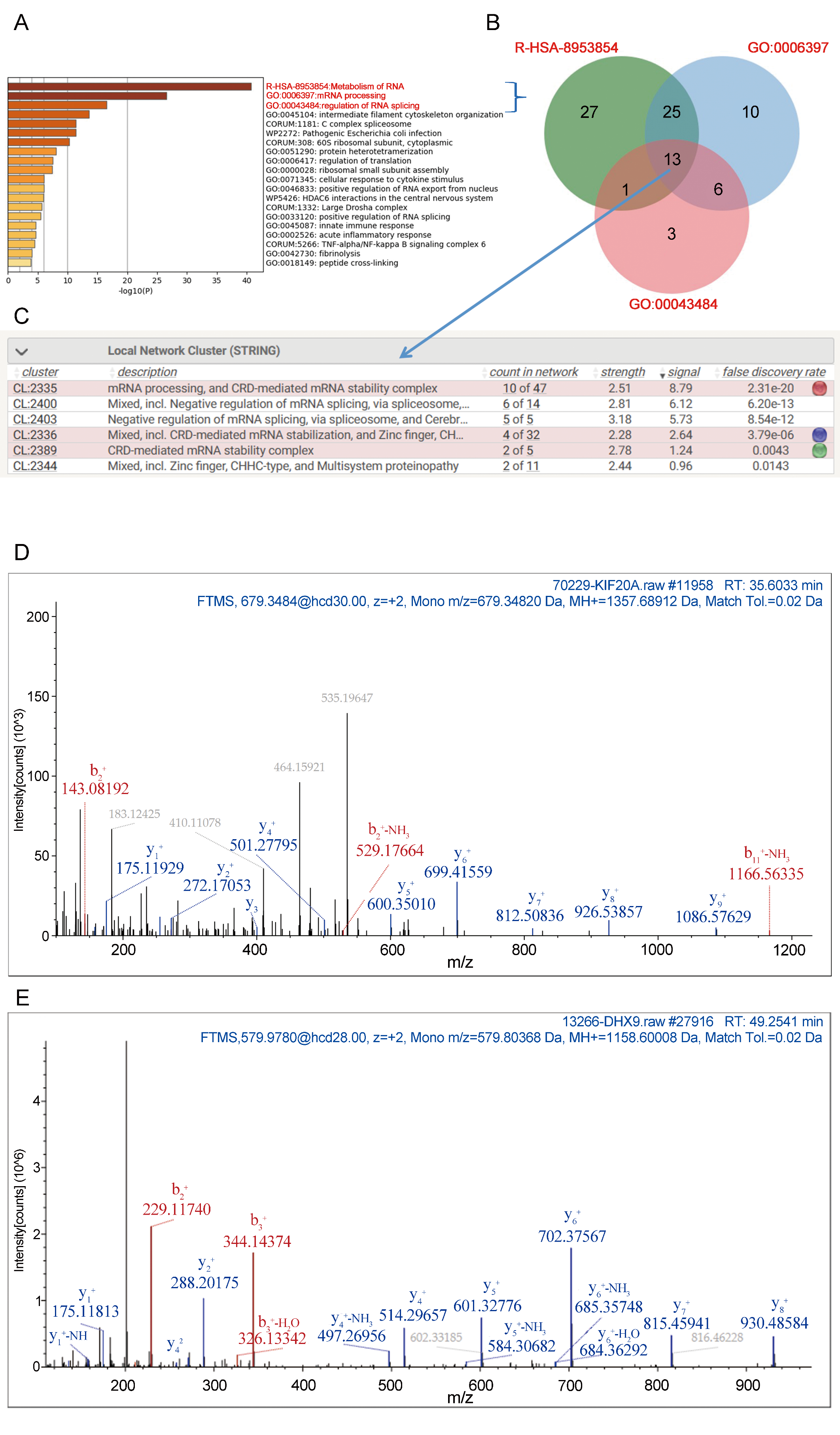


**Supplementary Figure 1 (A)** Pathway analysis of potential interacting proteins of KIF20A enriched by Metascape. **(B)** Venn diagram showing the overlapping proteins among the top 3 enriched pathways in Figure S1A. **(C)** Cluster analysis of STRING protein interaction networks. **(D)** Mass spectrometry peak analyses of the DHX9-derived peptide identified from KIF20A co-immunoprecipitation. **(E)** Mass spectrometry peak analyses of the KIF20A-derived peptide identified from DHX9 co-immunoprecipitation.

**
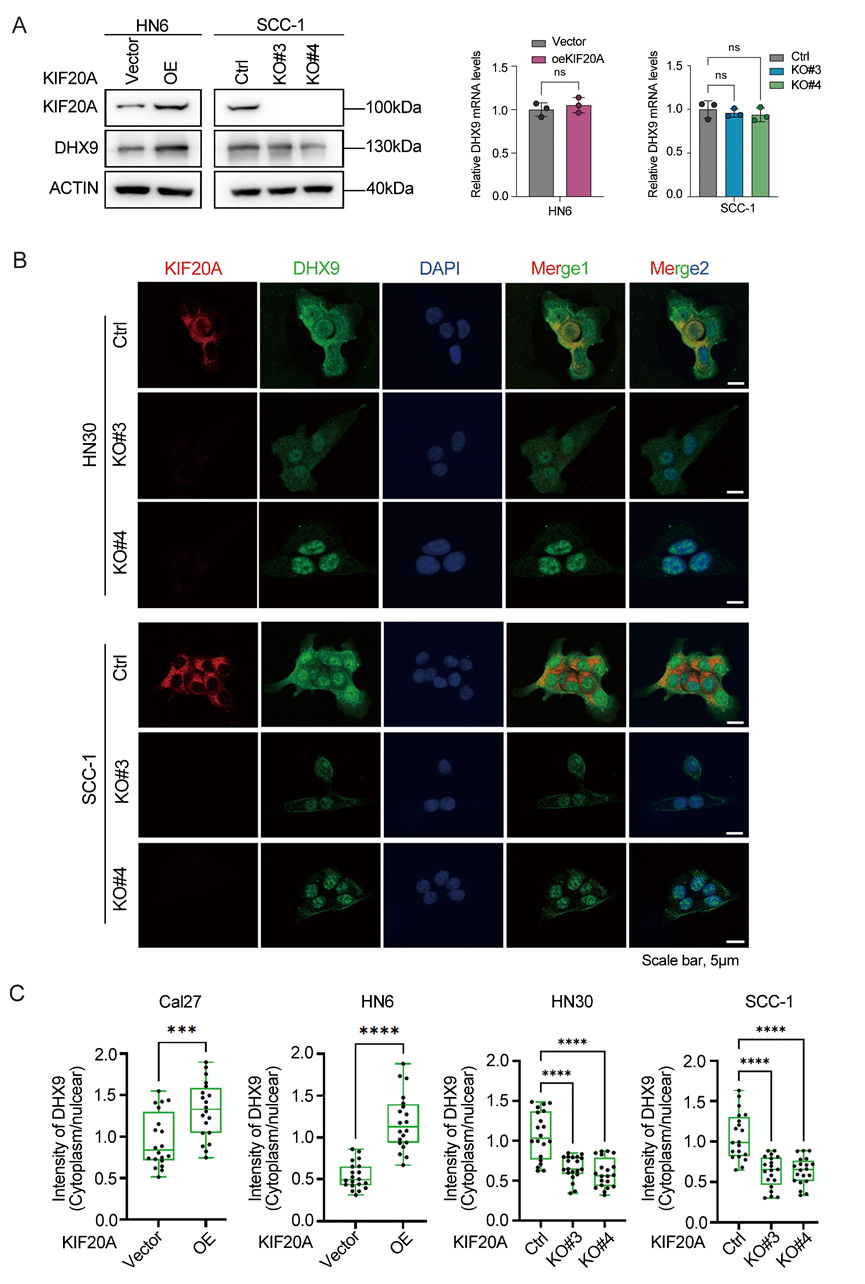
**

**Supplementary Figure 2 (A)** Western blot analysis of the expression levels of KIF20A and DHX9 in stable KIF20A high-expressing HN6 cells and stable KIF20A knockout SCC-1 cells, as well as RT-qPCR analysis of the mRNA level of DHX9. **(B)** Immunofluorescence staining of KIF20A (red), DHX9 (green), and DAPI (blue) in HN30 and SCC-1 cells with stable KIF20A knockout. Scale bar = 5μm.**(C)** Statistical analysis of DXH9 cytoplasmic/nuclear fluorescence intensity ratios in Cal27 and HN6 cells stably overexpressing KIF20A and in HN30 and SCC-1 cells stably knocking out KIF20A; n=20 cells. Statistical significance is denoted by *****P*<0.0001, ****P*<0.001, ns. not significant.

**
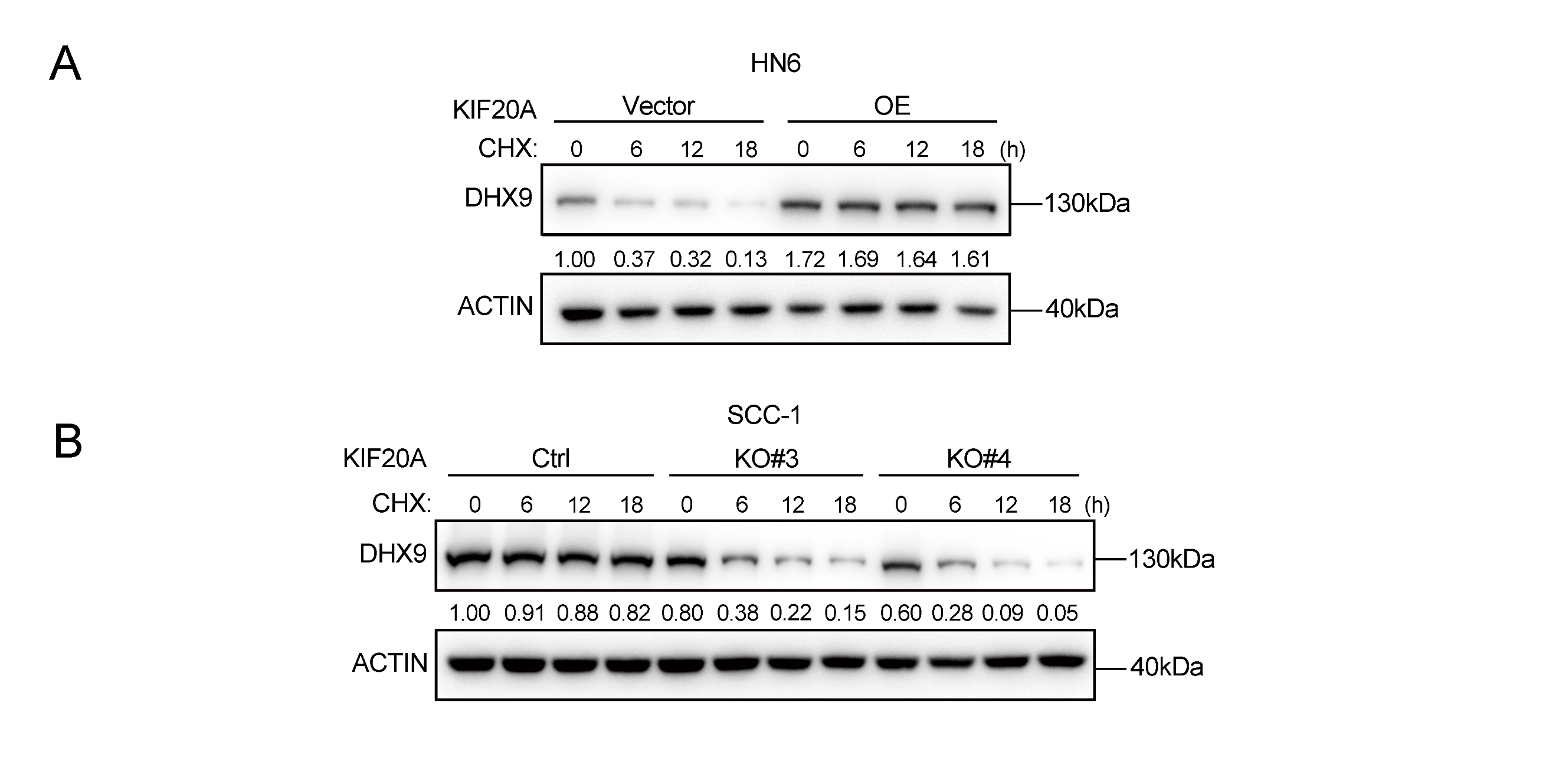
**

**Supplementary Figure 3 (A)** Western blot analysis showing the changes in DHX9 protein levels in stable KIF20A-overexpressing HN6 cells and control cells following CHX treatment at different time points. **(B)** Western blot analysis shows the changes in DHX9 protein levels in stable KIF20A-knockout SCC-1 cells and control cells following CHX treatment at different time points.

**
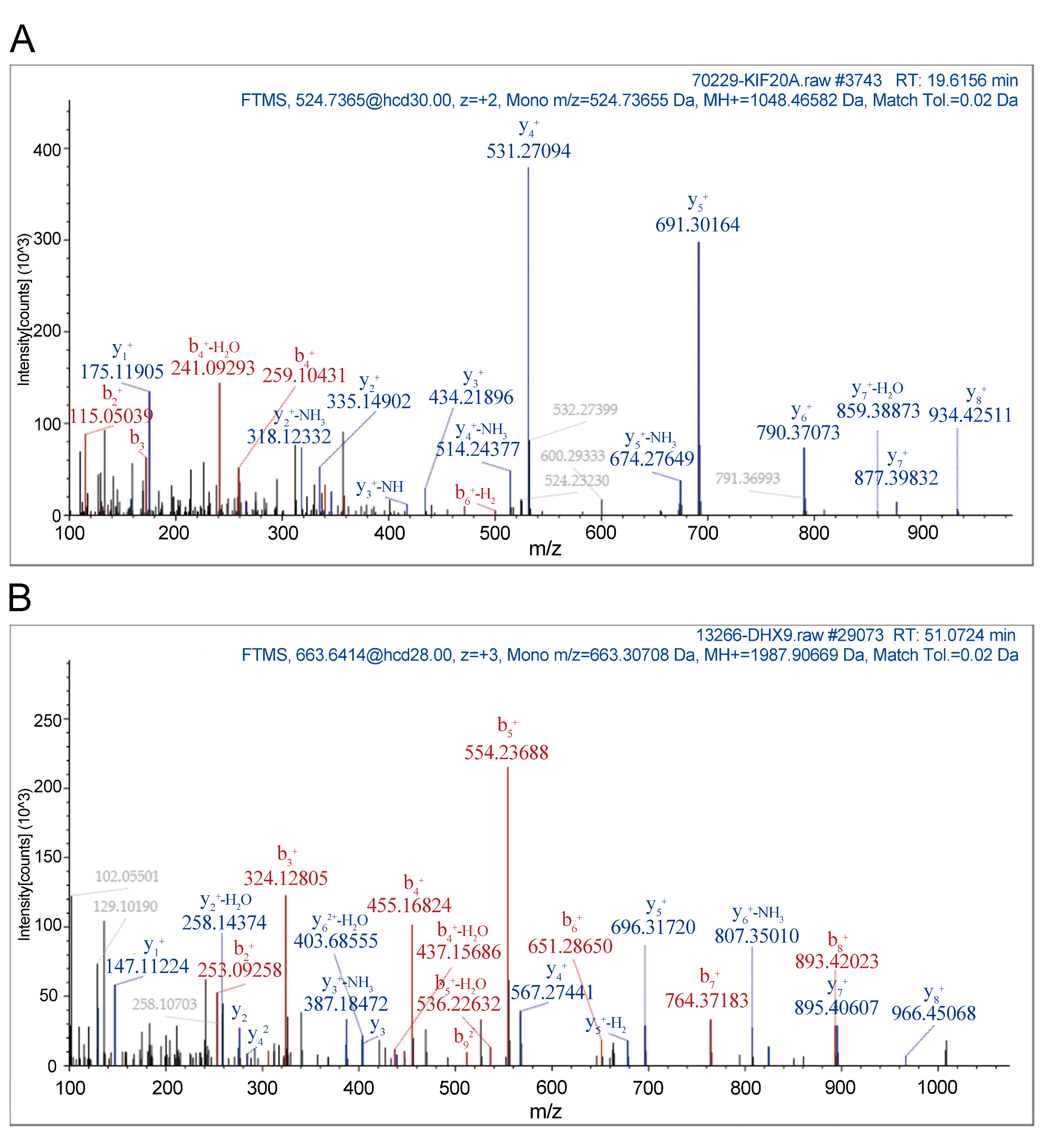
**

**Supplementary Figure 4 (A)** Mass spectrometry peak analyses of the TRIM21-derived peptide identified from KIF20A co-immunoprecipitation. **(B)** Mass spectrometry peak analyses of the TRIM21-derived peptide identified from DHX9 co-immunoprecipitation.


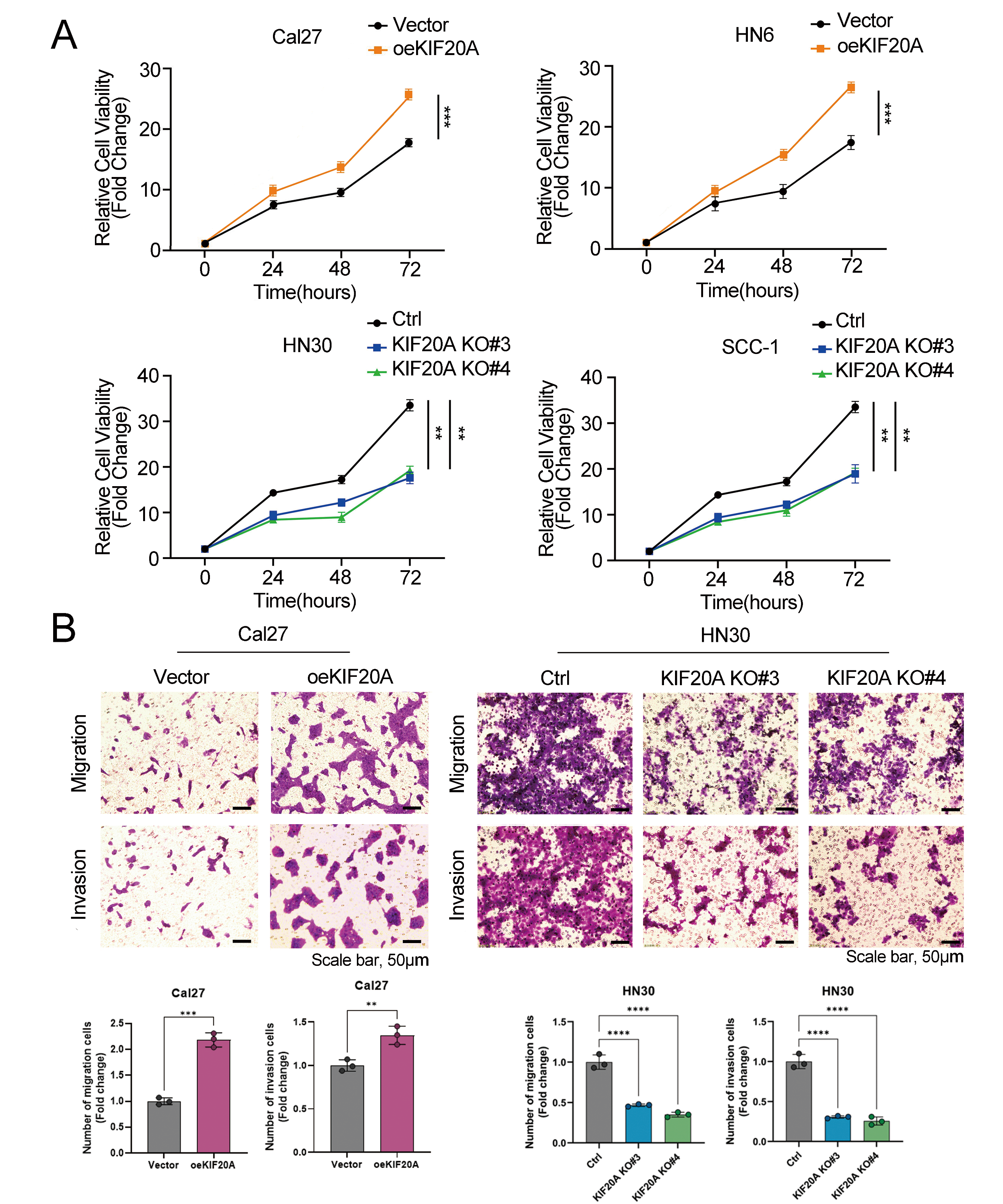
**Supplementary Figure 5 (A)** Cell proliferation of KIF20A-overexpressing stable lines Cal27 and HN6, and KIF20A-knockout stable lines HN30 and SCC-1. **(B)** Migration and invasion capacity of the KIF20A-overexpressing stable line Cal27 and the KIF20A-knockout stable line HN30. Statistical significance is denoted as: *****P* < 0.0001, ****P* < 0.001, ***P* < 0.01.


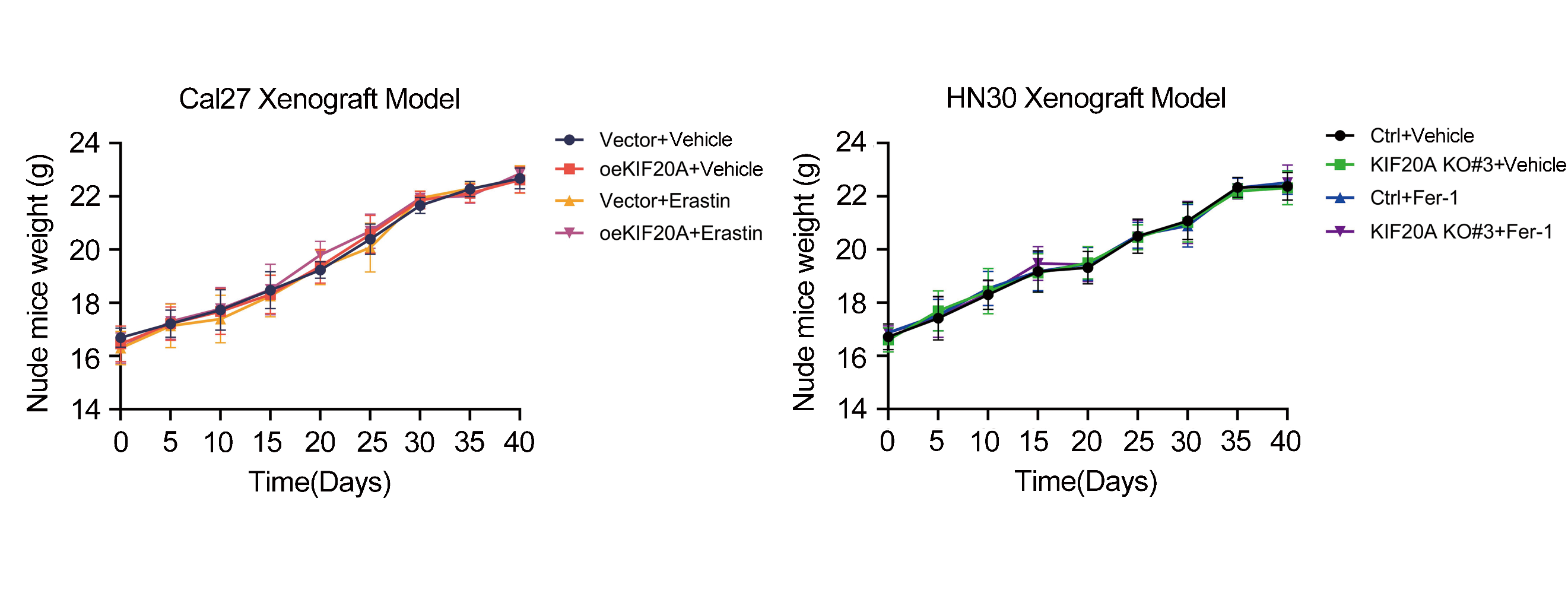


**Supplementary Figure 6** Body weight monitoring of nude mice bearing Cal27 (left) or HN30 (right) xenograft tumors.


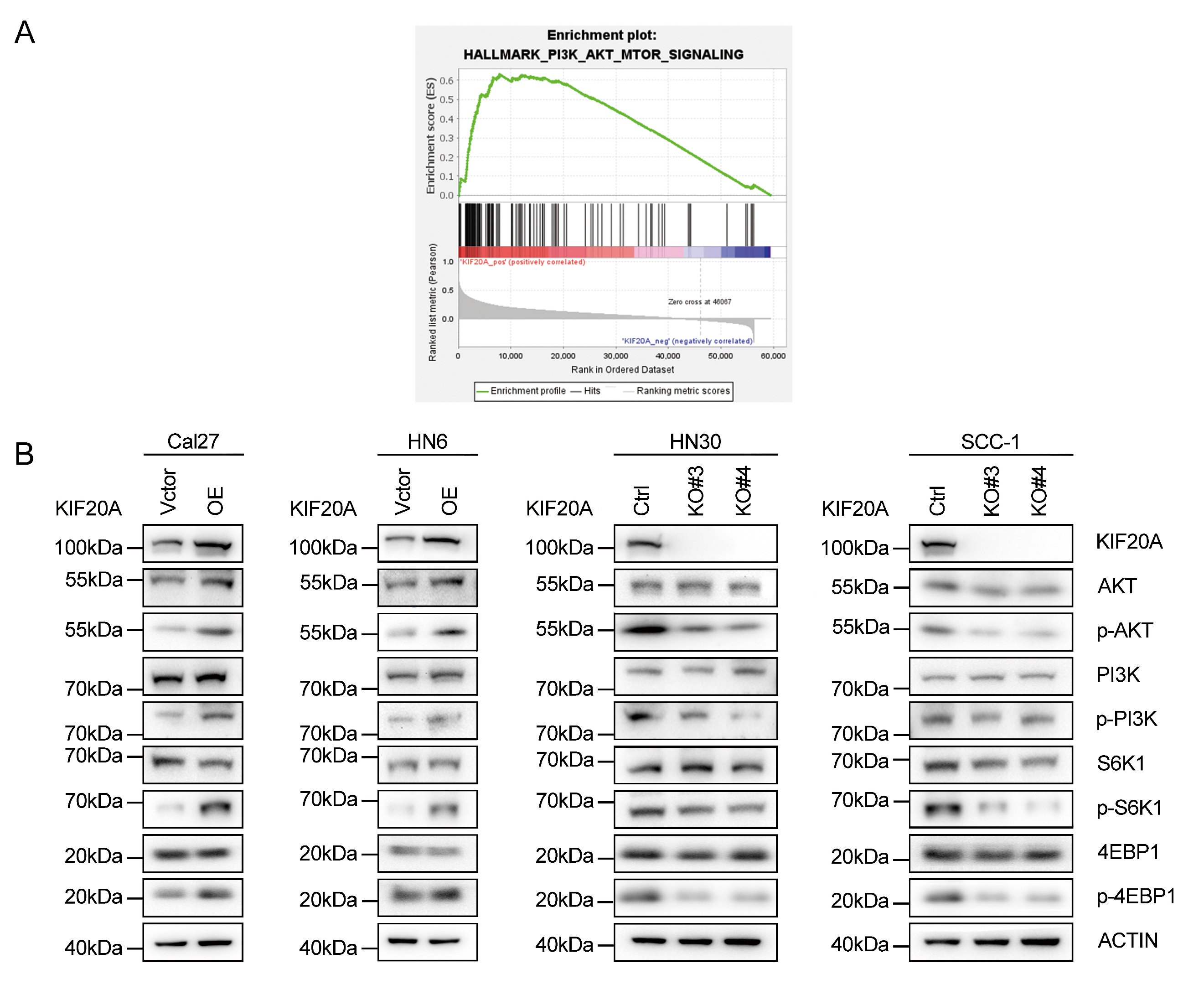


**Supplementary Figure 7 (A)** Gene set enrichment analysis (GSEA) of TCGA-OSCC transcriptomic data showing a positive association between KIF20A expression and the PI3K-AKT-mTOR signaling pathway. **(B)**Western blots showing expression levels of total and phosphorylated AKT, PI3K, S6K1 and 4EBP1 proteins in KIF20A‐overexpressed Cal27 and HN6, KIF20A‐knockout HN30 and SCC-1.

**
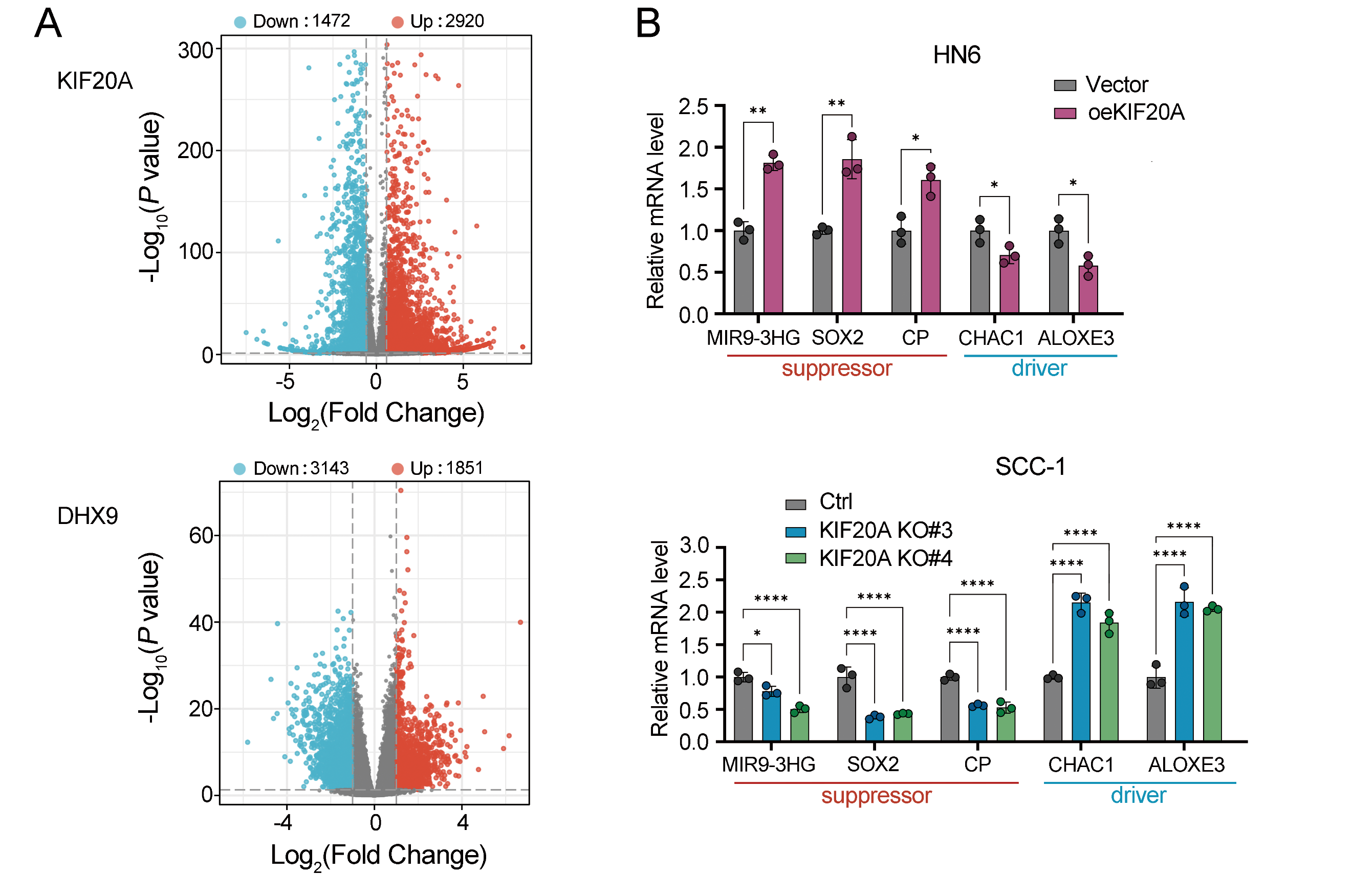
Supplementary Figure 8 (A)** Volcano plot of ifferentially expressed genes associated with |log_2_FC|≥1 and *P* <0.05 from sequencing data for Cal27 cells overexpressing either KIF20A or DHX9. **(B)** RT-qPCR analysis of mRNA levels of MIR9-3HG, SOX2, CP, CHAC1, ALOXE3 in stable KIF20A-overexpressing HN6 lines and knockout SCC-1 lines. Statistical significance is denoted by *****P*<0.0001, ***P* < 0.01, **P* < 0.05.

**
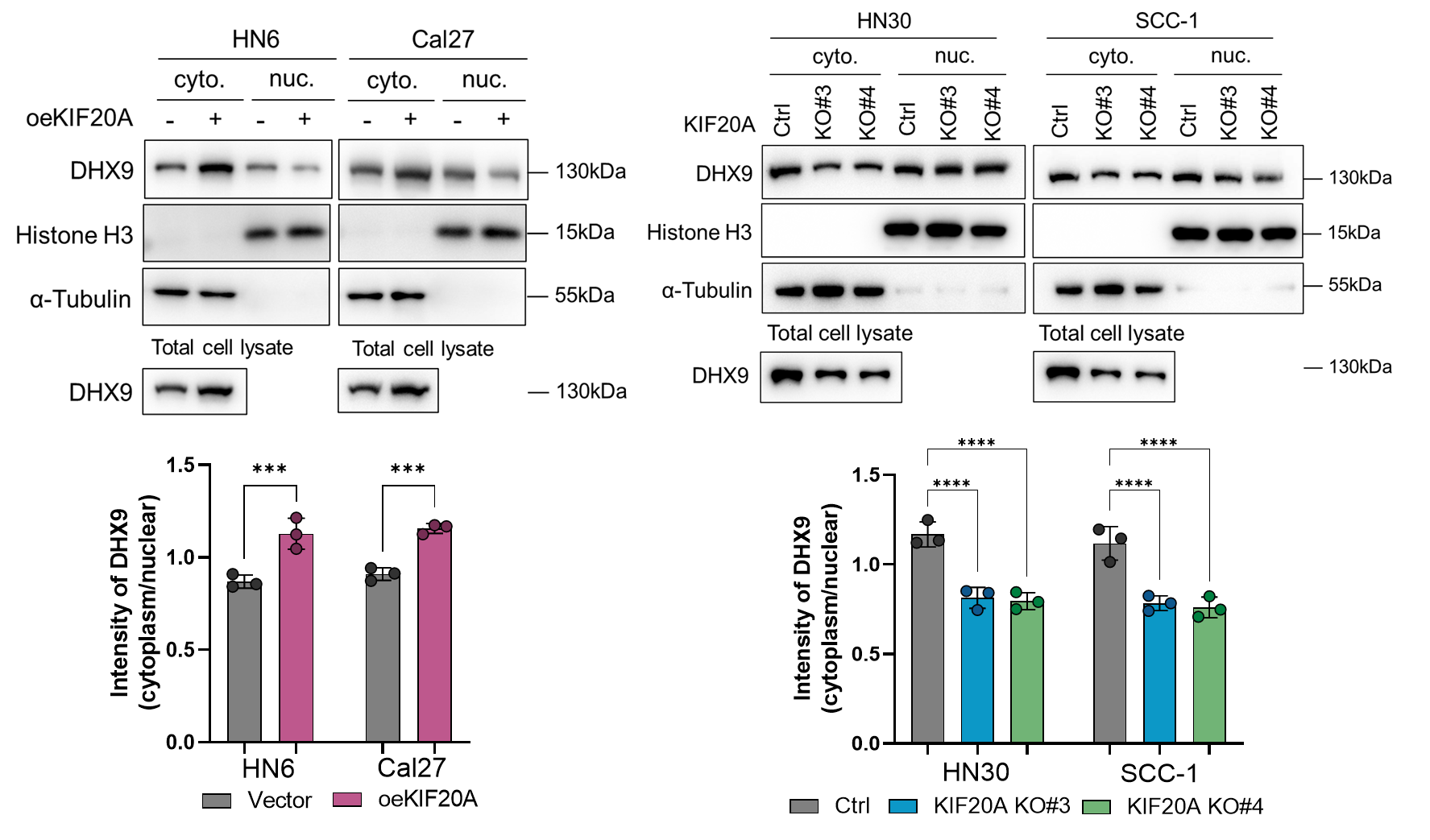
**

**Supplementary Figure 9.** Subcellular distribution of DHX9 in KIF20A-modulated cell lines.Subcellular fractionation was performed to assess DHX9 expression in the cytoplasm and nucleus of KIF20A overexpressing (HN6, Cal27) and knockout (HN30, SCC-1) cell lines. The upper panels show Western blot results, and the lower panels present statistical analysis of DHX9 distribution. The Cytoplasm/Nuclear ratio is calculated by comparing the expression levels of DHX9 in the cytosol and nucleus. Cytosolic and nuclear protein levels are normalized using α-Tubulin for cytosolic fractions (Cytosolic LC) and Histone H3 for nuclear fractions (Nuclear LC). The formula used is: Cyto./Nuc. = (X_Cyto._/Cytosolic LC) / (X_Nuc._/Nuclear LC). Statistical significance is denoted by *****P*<0.0001, ****P*<0.001.


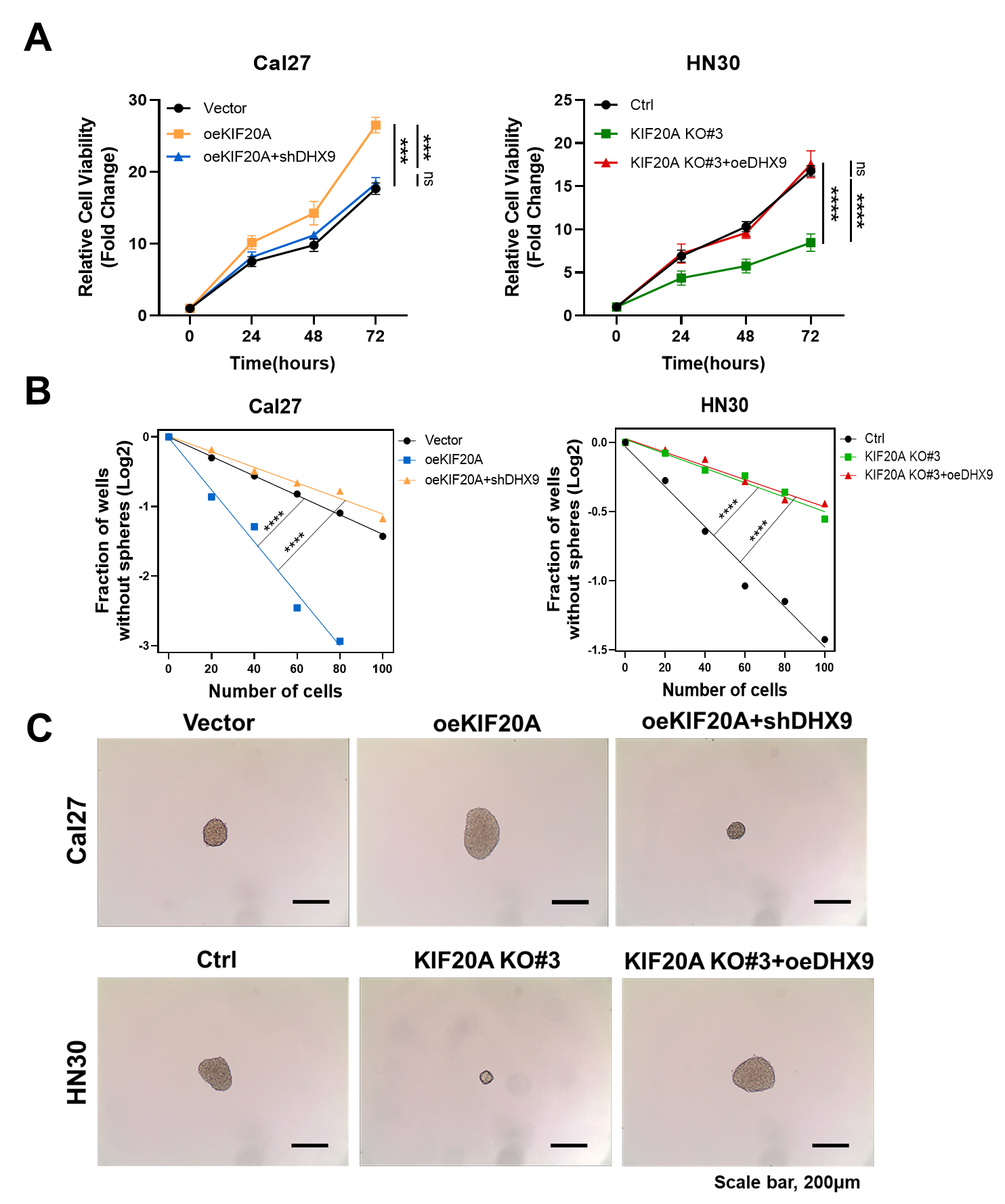


**Supplementary Figure 10. (A)** CCK-8 assays measuring cell viability in KIF20A-overexpressing Cal27 cells with or without DHX9 knockdown, and in KIF20A-knockout HN30 cells with or without DHX9 overexpression. **(B)** Extreme limiting dilution analysis (ELDA) determining stem cell frequency in the indicated groups. **(C)** Representative tumor-sphere images from the indicated groups. Scale bars, 200 μm. Statistical significance is denoted by *****P*<0.0001, ****P*<0.001, ns, not significant.


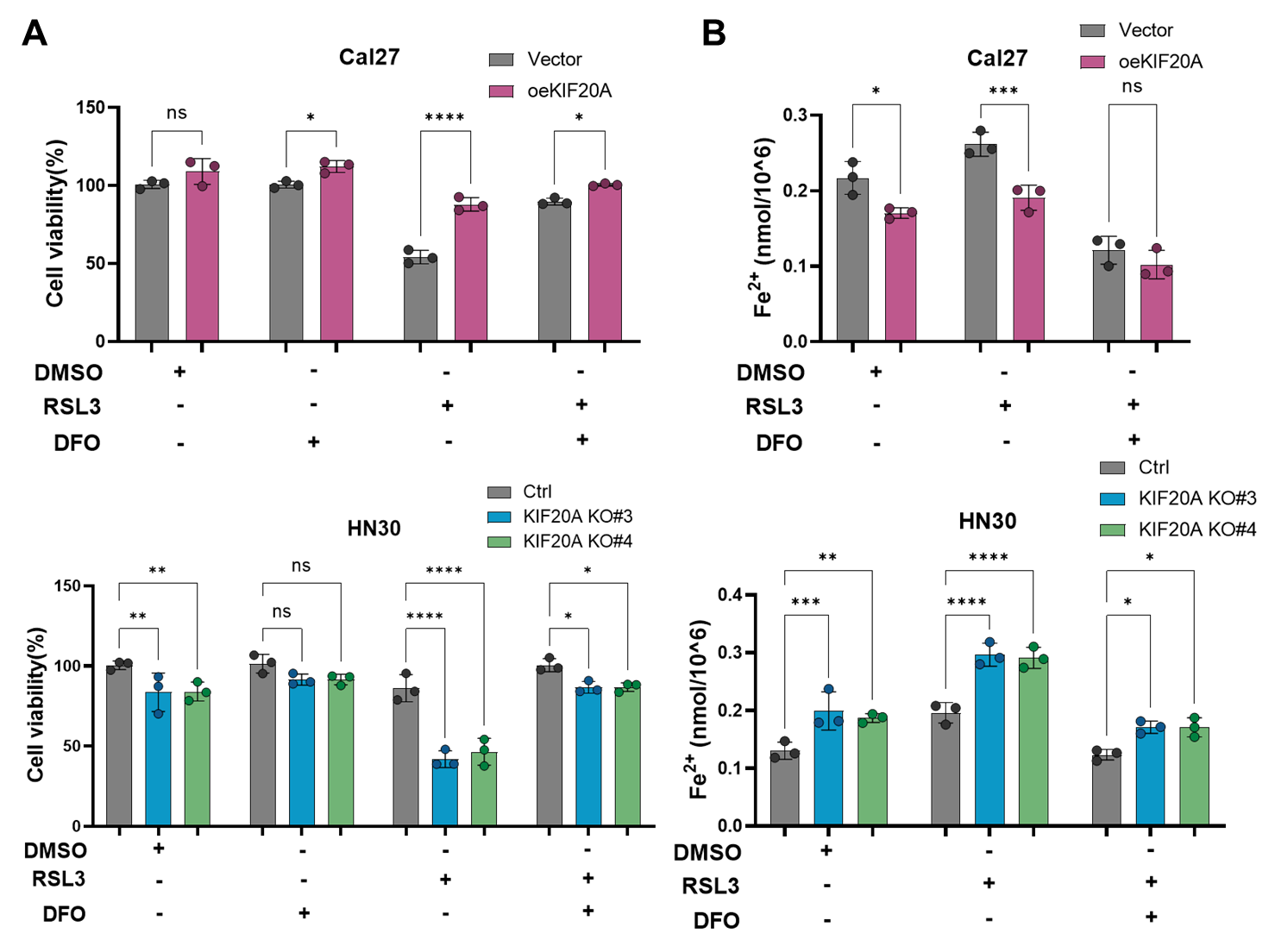
**Supplementary Figure 11**. (**A**) CCK-8 assay measuring cell viability in stable Cal27 KIF20A-overexpressing and HN30 knockout cell lines treated with RSL3, DFO, or their combination. (**B**) Ferrous iron levels were detected using a ferrous iron assay kit in stable Cal27 KIF20A-overexpressing and HN30 knockout cell lines treated with RSL3, DFO, or their combination. Statistical significance is denoted by *****P*<0.0001, ****P*<0.001, ***P* < 0.01, **P* < 0.05, ns. not significant.

**
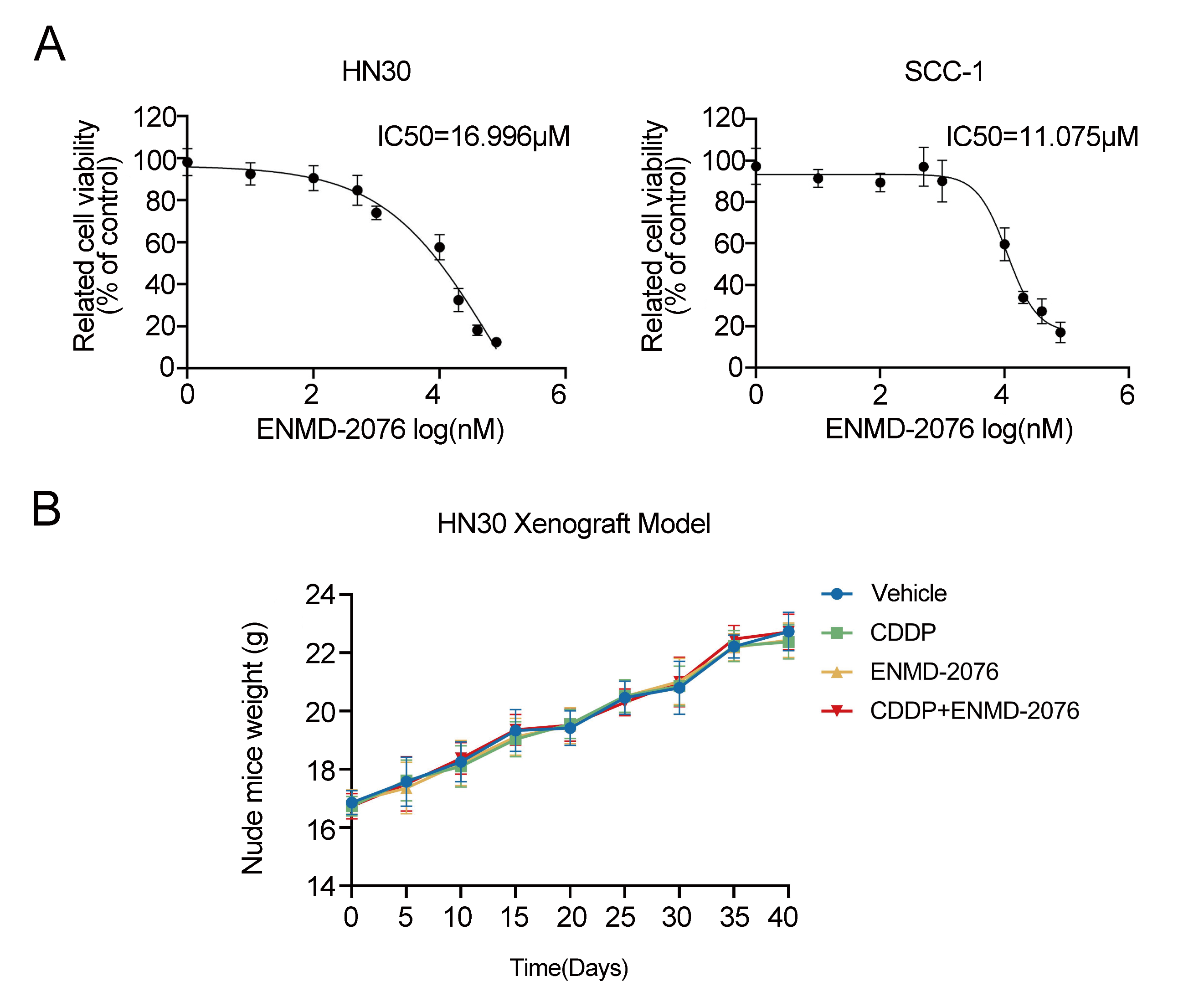
Supplementary Figure 12 (A)** CCK8 assay to detect the IC50 of ENMD-2076 in OSCC cell lines HN30 and SCC-1. **(B)** Body weight monitoring of nude mice bearing HN30 xenograft tumors.


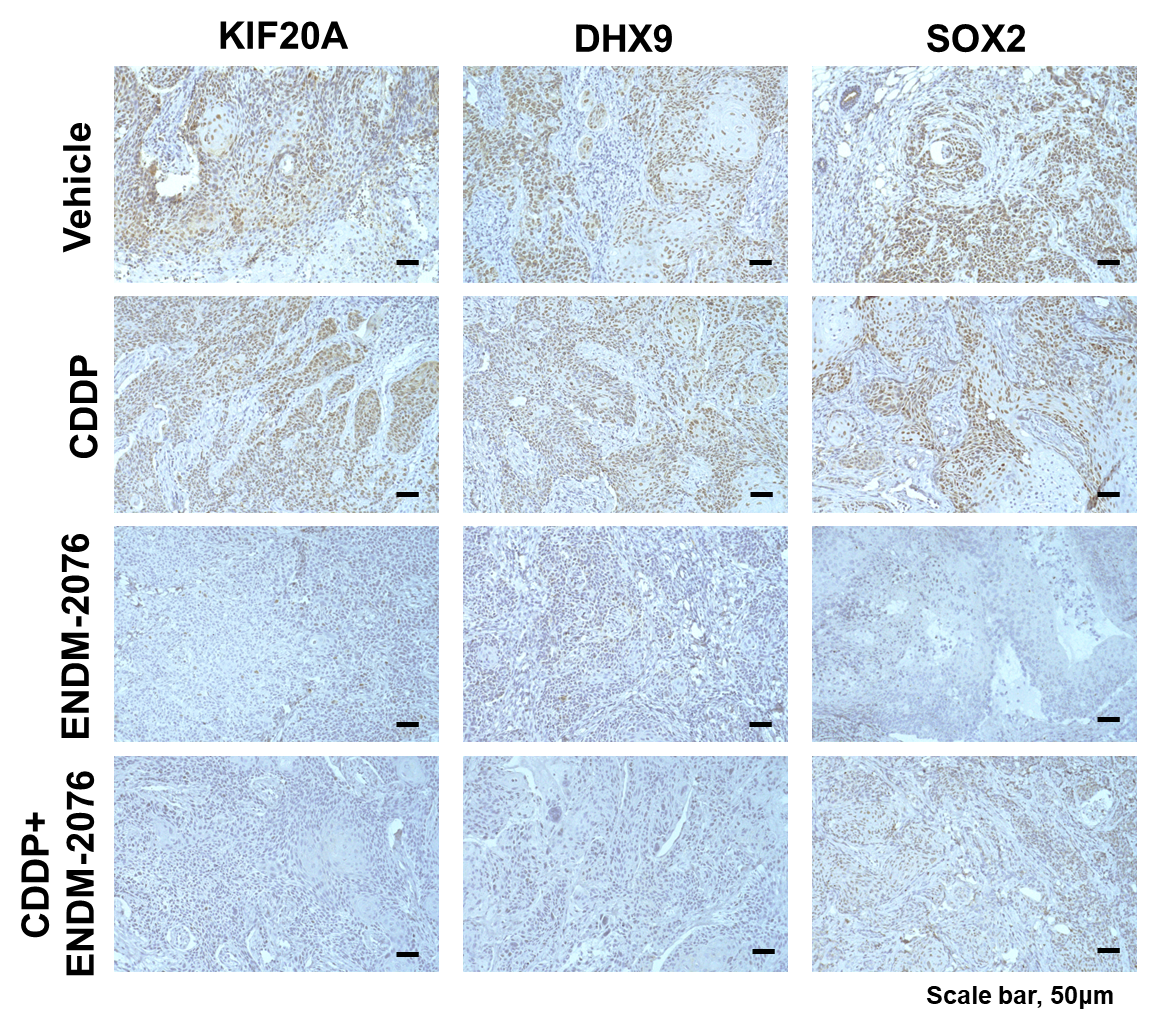


**Supplementary Figure 13.** Representative immunohistochemical (IHC) staining of KIF20A, DHX9, and SOX2 in xenograft tumors following the indicated drug treatments. Scale bars, 50 μm.

**Supplementary Table 1. Sequences of primers for real-time qPCR in this study.**

|  | | **Real-time qPCR** |
| --- | --- | --- |
| KIF20A | **F**orward primer | TCAACGACCACTTTGTCAAGCTCA |
|  | **R**everse primer | GCTGGTGGTCCAGGGGTCTTACT |
| SOX2 | **F**orward primer | GCTCGCAGACCTACATGAAC |
|  | **R**everse primer | GGACTTGACCACCGAACCCAT |
| DHX9 | **F**orward primer | AGCTGTGGCTACAGCGTTCGAT |
|  | **R**everse primer | CTGATTCCTCGAATGCCTGCTTC |
| ALOXE3 | **F**orward primer | GCTGCTCTTCAATGCCATCCCT |
|  | **R**everse primer | TGTCGTGAAGGTCTTATGGCACC |
| CHAC1 | **F**orward primer | GTGGTGACGCTCCTTGAAGATC |
|  | **R**everse primer | GAAGGTGACCTCCTTGGTATCG |
| MIR9-3HG | **F**orward primer | TGCTAGTCCAGTGCCATTCTTCG |
|  | **R**everse primer | AACCGACAGTGCTGGAGAGTGT |
| CP | **F**orward primer | CCCTCAAACAAGTCTTACGCTCC |
|  | **R**everse primer | CCAGGTAGAAGGTGGAATCCTC |
| *β*-actin | **F**orward primer | CACCATTGGCAATGAGCGGTTC |
|  | **R**everse primer | AGGTCTTTGCGGATGTCCACGT |

| ABCA13 | CDON | FGF21 | LUZP2 | PEDS1 | SCUBE3 |
| --- | --- | --- | --- | --- | --- |
| ABCA3 | CECR2 | FGFR2 | MAP7D2 | PELI2 | SEMA6A |
| ABCC5 | CEL | FOXP2 | MCCC1-AS1 | PGAP1 | SIX3 |
| ADAM22 | CES3 | FREM2 | MDGA1 | PKDCC | SLC16A14 |
| ADAMTS13 | CLDN18 | FRRS1L | MEF2C | PKDREJ | SMIM38 |
| ADARB2 | CLDN20 | FZD7 | MEX3A | PLA2G6 | SNX2P1 |
| AK7 | CMTM1 | GALNT9 | MIR9-3HG | PLAC8 | SOX13 |
| AKR1C1 | CNTNAP3C | GCLC | MNS1 | PLCH1 | SOX2 |
| AKR1C2 | CP | GLI2 | MORN3 | PML | SOX2-OT |
| AKR1C3 | CRACD | GLS2 | MRAP2 | PNCK | SPATA46 |
| ALDH1A1 | CSRNP3 | GPR161 | MUC1 | PPARA | STAG3 |
| ALDH3A2 | CXXC4 | HCAR1 | MUC16 | PPARD | STC1 |
| ALX4 | CYP26A1 | HELLS | MUC2 | PPM1H | SUSD4 |
| ANKRD18EP | CYP2S1 | HIP1 | MYB | PRIMA1 | SYCP2 |
| ANKRD20A1 | CYP4F11 | HMSD | MYRF | PROM2 | SYNGR3 |
| ANKRD36B | CYP4F3 | HOXA13 | NAALADL2 | PRSS21 | SYT10 |
| ANKRD36C | DACH1 | HOXB13 | NCAPD2 | PTCH1 | TBX4 |
| ANO4 | DLEC1 | HOXD12 | NKAIN2 | PTPRS | TCIM |
| ARHGAP24 | DLGAP1 | HSPB1 | NOS2 | PXYLP1 | TET1 |
| ARHGEF26 | DMC1 | IDH2 | NR4A1 | QRFPR | TFRC |
| ASB9 | DMRT1 | KCNH1 | NR5A2 | RAB6B | TGFBR3 |
| ATRNL1 | DNAJC22 | KIAA0319 | NTRK2 | RADX | TMEM161B-DT |
| B4GALNT2 | DOC2A | KIF20A | NUDT11 | RAET1K | TMEM178B |
| BRDT | DPY19L2P2 | KLHL13 | NUPR1 | RASSF9 | TMEM255A |
| C2CD4D-AS1 | DUSP5P1 | KRT19 | OLFM1 | RCOR2 | TOP2A |
| CA9 | ECH1 | LIFR | OR1F1 | RNF150 | TRIB2 |
| CALB1 | EYA1 | LINC01305 | OR2A7 | RORB | TRIM9 |
| CBS | FADS2 | LINC01833 | OTX1 | SALL2 | USP35 |
| CCDC187 | FAM153A | LINC02068 | PARP10 | SBK1 | VTCN1 |
| CCDC74A | FAM72A | LIPI | PCDH11X | SBSPON | WDR49 |
| CCDC74B | FAM72B | LOXL4 | PCDHGB1 | SCD | ZNF887P |
| CCSER1 | FGF19 | LRRCC1 | PCDHGB7 | SCIN |  |

**Supplementary Table 2 Ferroptosis-related Upregulated Gene Set Co-regulated by KIF20A and DHX9**

**Supplementary Table 3 Ferroptosis-related downregulated gene set co-regulated by KIF20A and DHX9**

| ANKRD1 | FLG | ZNHIT2 | ZGLP1 | SLC39A14 | DUOX1 |
| --- | --- | --- | --- | --- | --- |
| H4C11 | PRSS3 | LINC02742 | ITIH4 | BRPF1 | ZFAS1 |
| TMEM86B | SPRR2D | EEF1DP5 | KRT80 | TFRC | COX4I2 |
| NWD2 | SLAMF7 | MMP24OS | PPP1R14B-AS1 | SLC1A5 | AQP3 |
| TREX2 | OR2L2 | SFTA1P | HSPB8 | TLR4 | CYP4F8 |
| MYPN | SLC25A34 | CCL5 | RNF223 | LYRM1 | ALOX12B |
| IL1RL1 | CYSRT1 | ACP7 | TUBA4A | GOT1 | LCE2C |
| HAL | PPIF | ABHD14A | IL6 | TIMM9 | CHAC1 |
| RASL10A | UPP1 | SDSL | KLF2 | CLTRN | ALOXE3 |
| S100P | GPX3 | ACTN2 | SLC7A11 | HRAS |  |
| DIRAS1 | HRNR | SPRR1A | DDR2 | IFNG |  |
| LURAP1L | RPL13AP20 | SNHG15 | SLC38A1 | ASMTL-AS1 |  |

**Supplementary Table 4. Effect and CI of ENMD-2076 and CDDP combined treatment in HN30**

| Dose ENMD-2076 | Dose CDDP | Effect | CI |
| --- | --- | --- | --- |
| 0 | 10μM | 0.83 | 0.694 |
| 3 | 10μM | 0.76 | 0.794 |
| 6 | 10μM | 0.71 | 0.563 |
| 9 | 10μM | 0.66 | 0.748 |
| 12 | 10μM | 0.58 | 0.845 |
| 15 | 10μM | 0.53 | 0.694 |
| 18 | 10μM | 0.52 | 0.601 |
| 21 | 10μM | 0.46 | 0.647 |
| 24 | 10μM | 0.43 | 0.585 |
| 0 | 20μM | 0.68 | 0.852 |
| 3 | 20μM | 0.65 | 0.803 |
| 6 | 20μM | 0.62 | 0.910 |
| 9 | 20μM | 0.57 | 0.850 |
| 12 | 20μM | 0.54 | 0.740 |
| 15 | 20μM | 0.52 | 0.702 |
| 18 | 20μM | 0.51 | 0.604 |
| 21 | 20μM | 0.45 | 0.522 |
| 24 | 20μM | 0.41 | 0.309 |
| 0 | 30μM | 0.56 | 0.806 |
| 3 | 30μM | 0.51 | 0.743 |
| 6 | 30μM | 0.48 | 0.609 |
| 9 | 30μM | 0.45 | 0.538 |
| 12 | 30μM | 0.41 | 0.454 |
| 15 | 30μM | 0.37 | 0.462 |
| 18 | 30μM | 0.33 | 0.418 |
| 21 | 30μM | 0.29 | 0.377 |
| 24 | 30μM | 0.25 | 0.243 |

CDDP: Cisplatin, CI: Combination index.

**Supplementary Table 5. Effect and ENMD-2076 and CDDP combined treatment in SCC-1**

| Dose ENMD-2076 | Dose CDDP | Effect | CI |
| --- | --- | --- | --- |
| 0 | 10μM | 0.87 | 0.780 |
| 3 | 10μM | 0.79 | 0.847 |
| 6 | 10μM | 0.72 | 0.738 |
| 9 | 10μM | 0.69 | 0.780 |
| 12 | 10μM | 0.63 | 0.646 |
| 15 | 10μM | 0.6 | 0.663 |
| 18 | 10μM | 0.58 | 0.548 |
| 21 | 10μM | 0.53 | 0.458 |
| 24 | 10μM | 0.45 | 0.319 |
| 0 | 20μM | 0.78 | 0.895 |
| 3 | 20μM | 0.71 | 0.903 |
| 6 | 20μM | 0.67 | 0.937 |
| 9 | 20μM | 0.61 | 0.716 |
| 12 | 20μM | 0.58 | 0.839 |
| 15 | 20μM | 0.53 | 0.609 |
| 18 | 20μM | 0.42 | 0.470 |
| 21 | 20μM | 0.37 | 0.366 |
| 24 | 20μM | 0.33 | 0.198 |
| 0 | 30μM | 0.55 | 0.819 |
| 3 | 30μM | 0.53 | 0.786 |
| 6 | 30μM | 0.51 | 0.730 |
| 9 | 30μM | 0.46 | 0.674 |
| 12 | 30μM | 0.41 | 0.607 |
| 15 | 30μM | 0.36 | 0.517 |
| 18 | 30μM | 0.24 | 0.481 |
| 21 | 30μM | 0.21 | 0.358 |
| 24 | 30μM | 0.19 | 0.109 |

CDDP: Cisplatin, CI: Combination index.
