# Supplementary material for: KIF20A inhibits TRIM21-dependent ubiquitination of DHX9 to boost SOX2 stability, enhancing OSCC stemness and ferroptosis resistance
Source: Cell Death Dis. 2026 Feb 11;17(1):218. doi: 10.1038/s41419-026-08467-w (PMC12920667; doi:10.1038/s41419-026-08467-w)
Supplement: Supplementary file 3 — Uncropped western blots [file 41419_2026_8467_MOESM3_ESM.pdf]

A

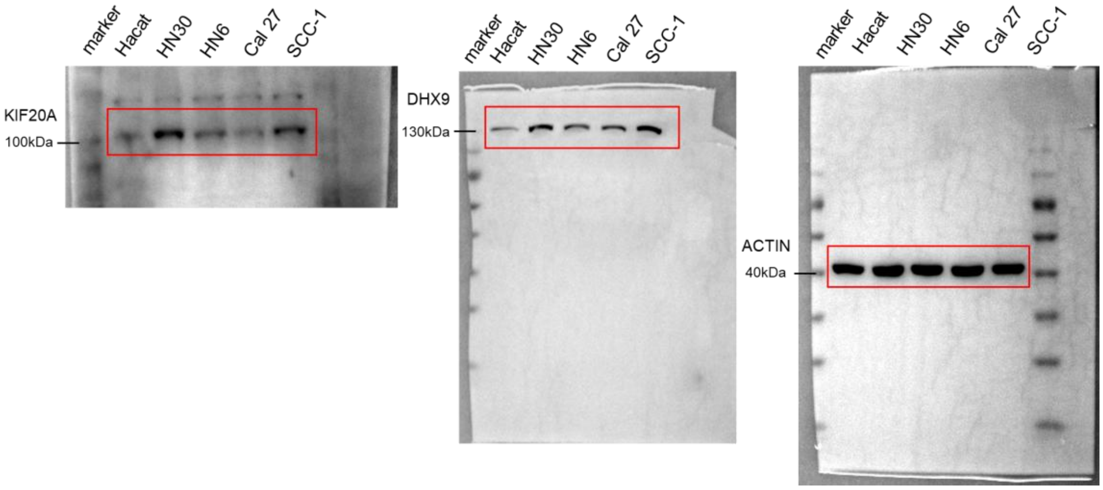
marker
Hacat
HN30
HN6
Cal 27
SCC-1
KIF20A
100kDa
DHX9
130kDa
ACTIN
40kDa

B

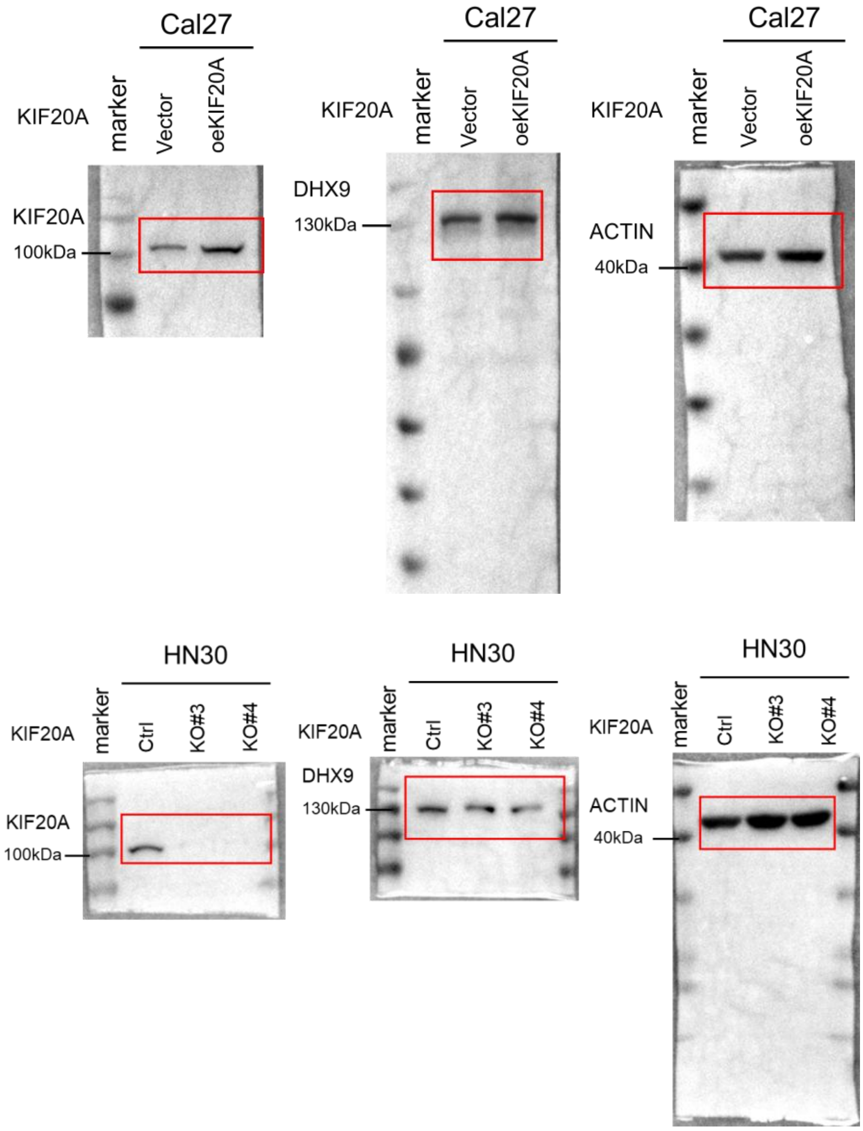
Cal27
KIF20A
marker
Vector
oeKIF20A
KIF20A
100kDa
DHX9
130kDa
ACTIN
40kDa
HN30
Ctrl
KO#3
KO#4

C

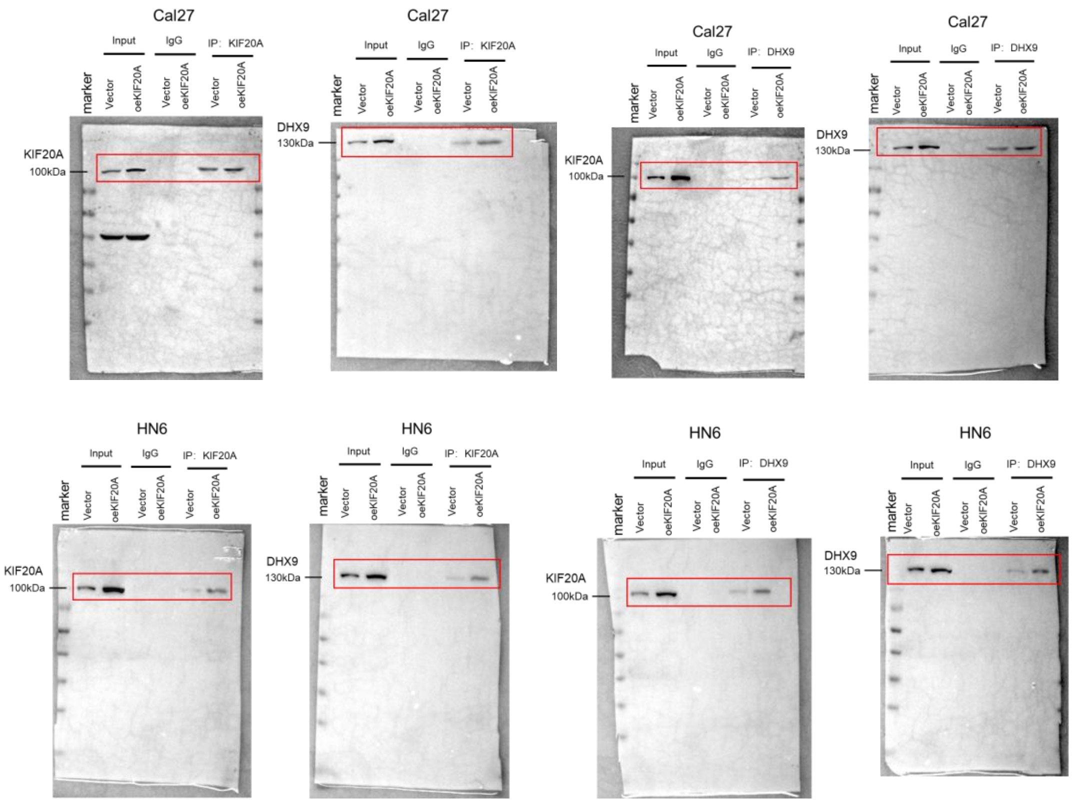
Cal27
Input
IgG
IP: KIF20A
IP: DHX9
marker
Vector
oeKIF20A
KIF20A
100kDa
DHX9
130kDa
HN6

D

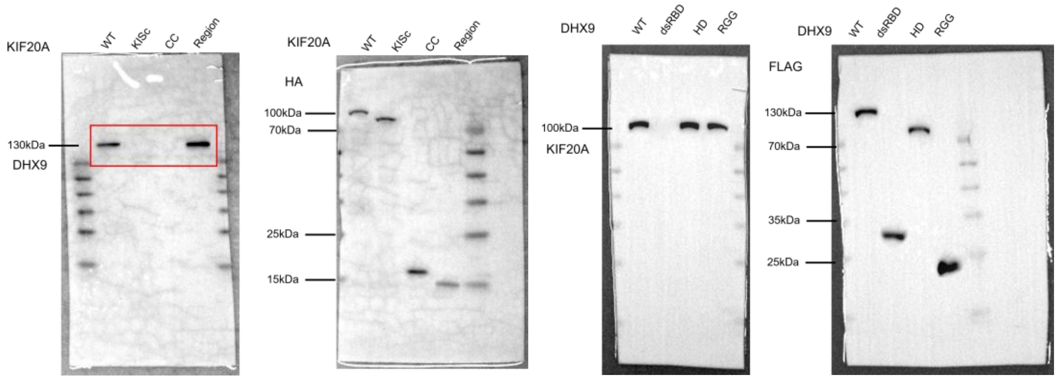
KIF20A
WT
KISc
CC
Region
130kDa
DHX9
HA
100kDa
70kDa
25kDa
15kDa
dsRBD
HD
RGG
KIF20A
FLAG
130kDa
70kDa
35kDa
25kDa

E

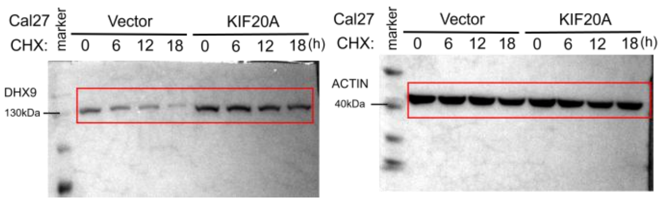
Cal27
CHX:
marker
Vector
KIF20A
0
6
12
18
(h)
DHX9
130kDa
ACTIN
40kDa

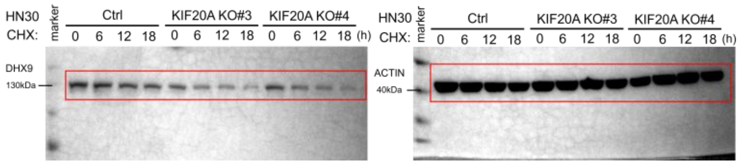
HN30
CHX:
marker
Ctrl
KIF20A KO#3
KIF20A KO#4
0 6 12 18 0 6 12 18 0 6 12 18 (h)
DHX9
130kDa
ACTIN
40kDa

F

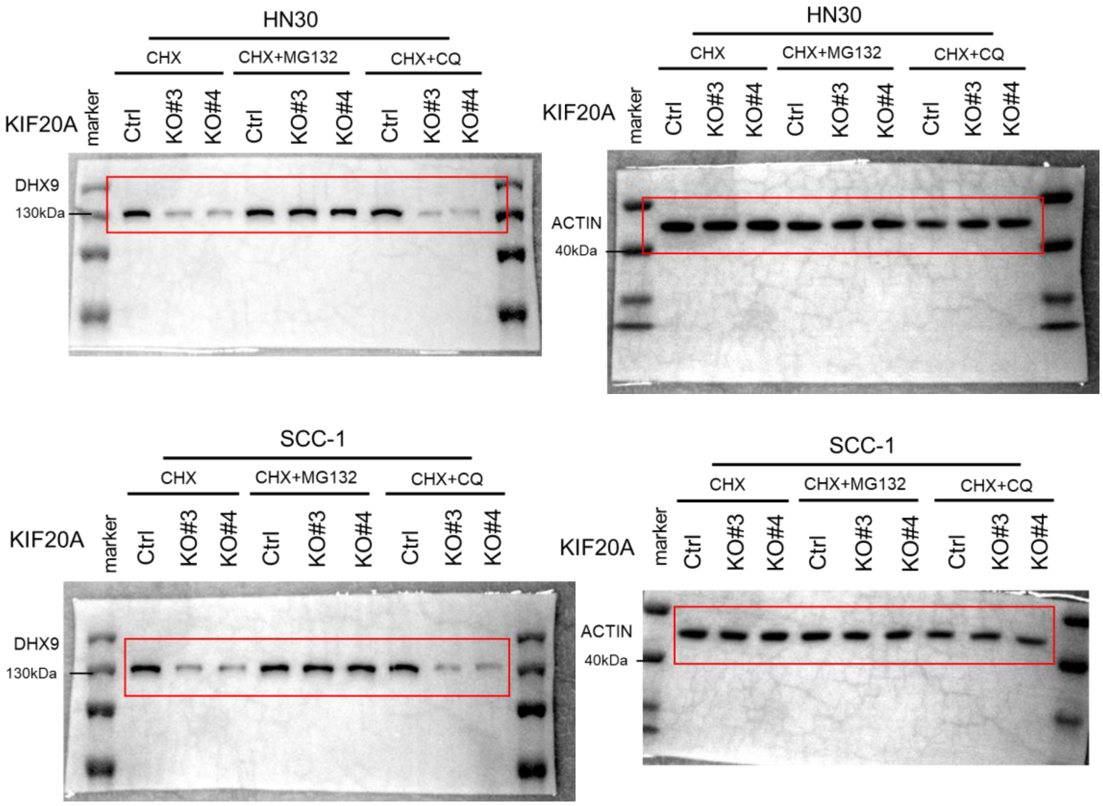
HN30
CHX
CHX+MG132
CHX+CQ
KIF20A
marker
Ctrl
KO#3
KO#4
DHX9
130kDa
ACTIN
40kDa
SCC-1

G

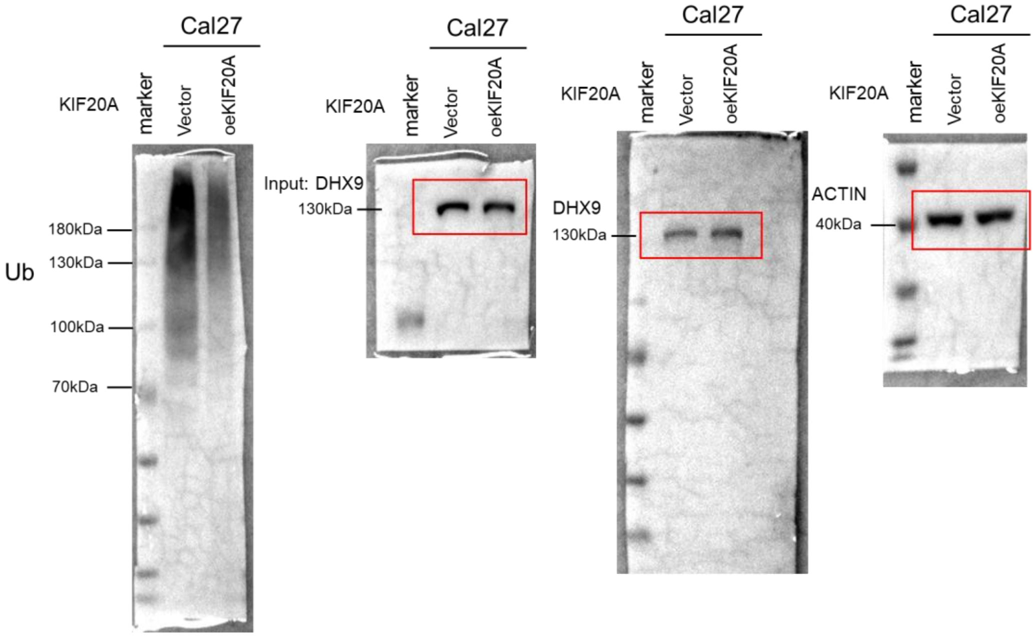
Cal27
KIF20A
marker
Vector
oeKIF20A
Ub
180kDa
130kDa
100kDa
70kDa
Input: DHX9
130kDa
DHX9
ACTIN
40kDa

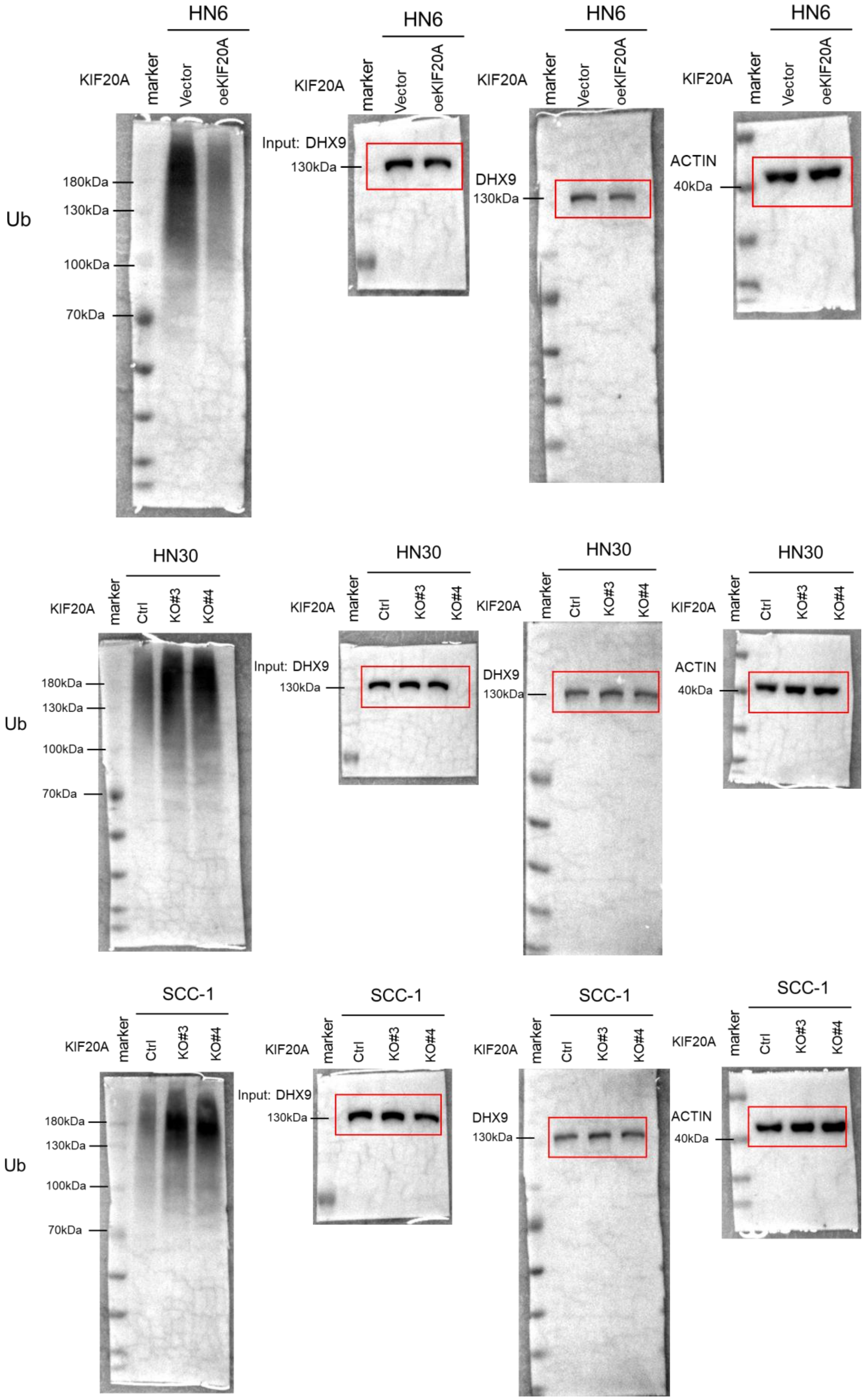
HN6
KIF20A
marker
Vector
oeKIF20A
Ub
180kDa
130kDa
100kDa
70kDa
Input: DHX9
130kDa
DHX9
130kDa
ACTIN
40kDa
HN30
Ctrl
KO#3
KO#4
SCC-1

H

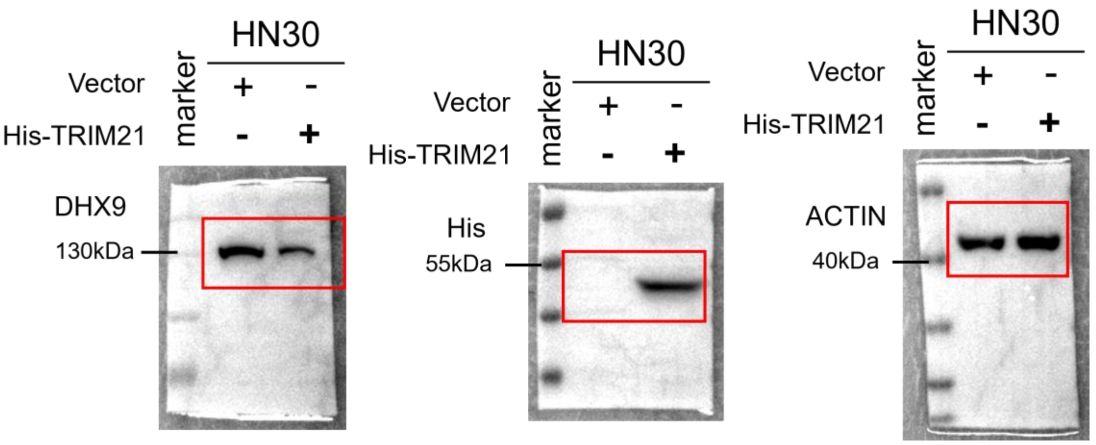
HN30
Vector
His-TRIM21
marker
+
-
DHX9
130kDa
His
55kDa
ACTIN
40kDa

I

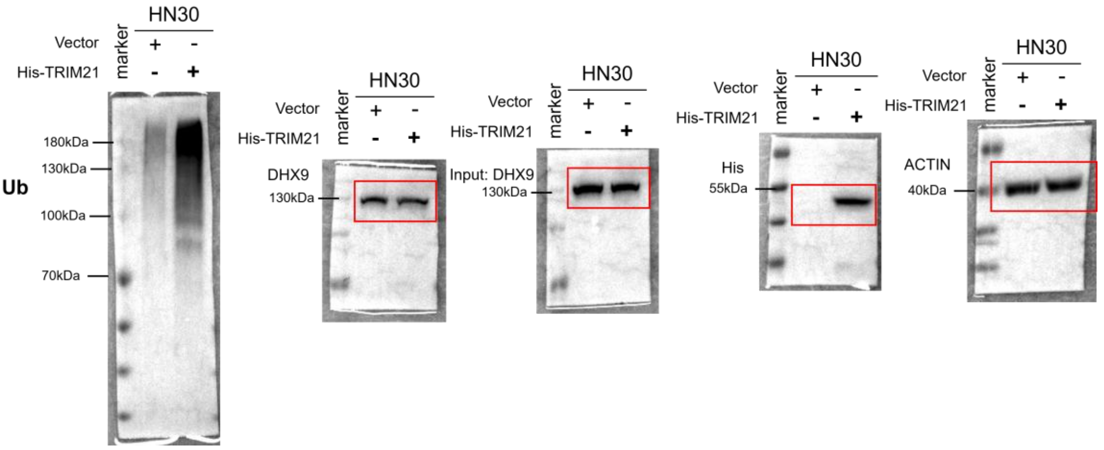
HN30
Vector
His-TRIM21
marker
+
-
Ub
180kDa
130kDa
100kDa
70kDa
DHX9
130kDa
Input: DHX9
His
55kDa
ACTIN
40kDa

J

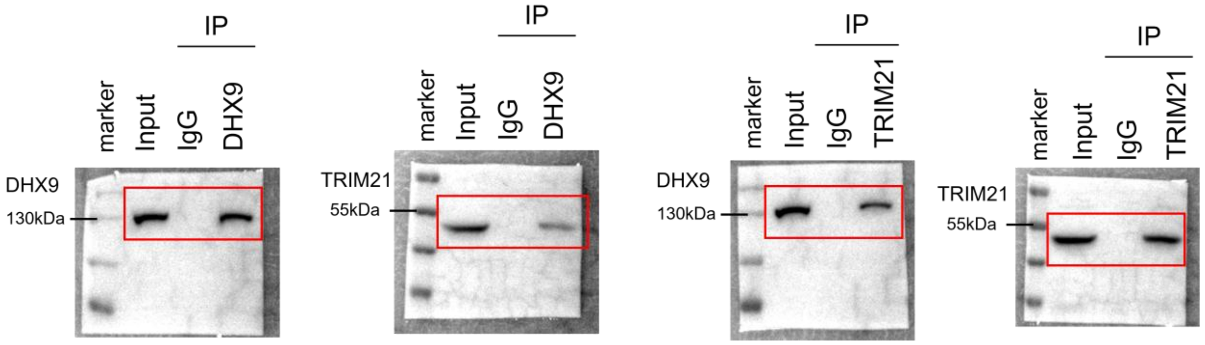
IP
marker
Input
IgG
DHX9
TRIM21
130kDa
55kDa

K

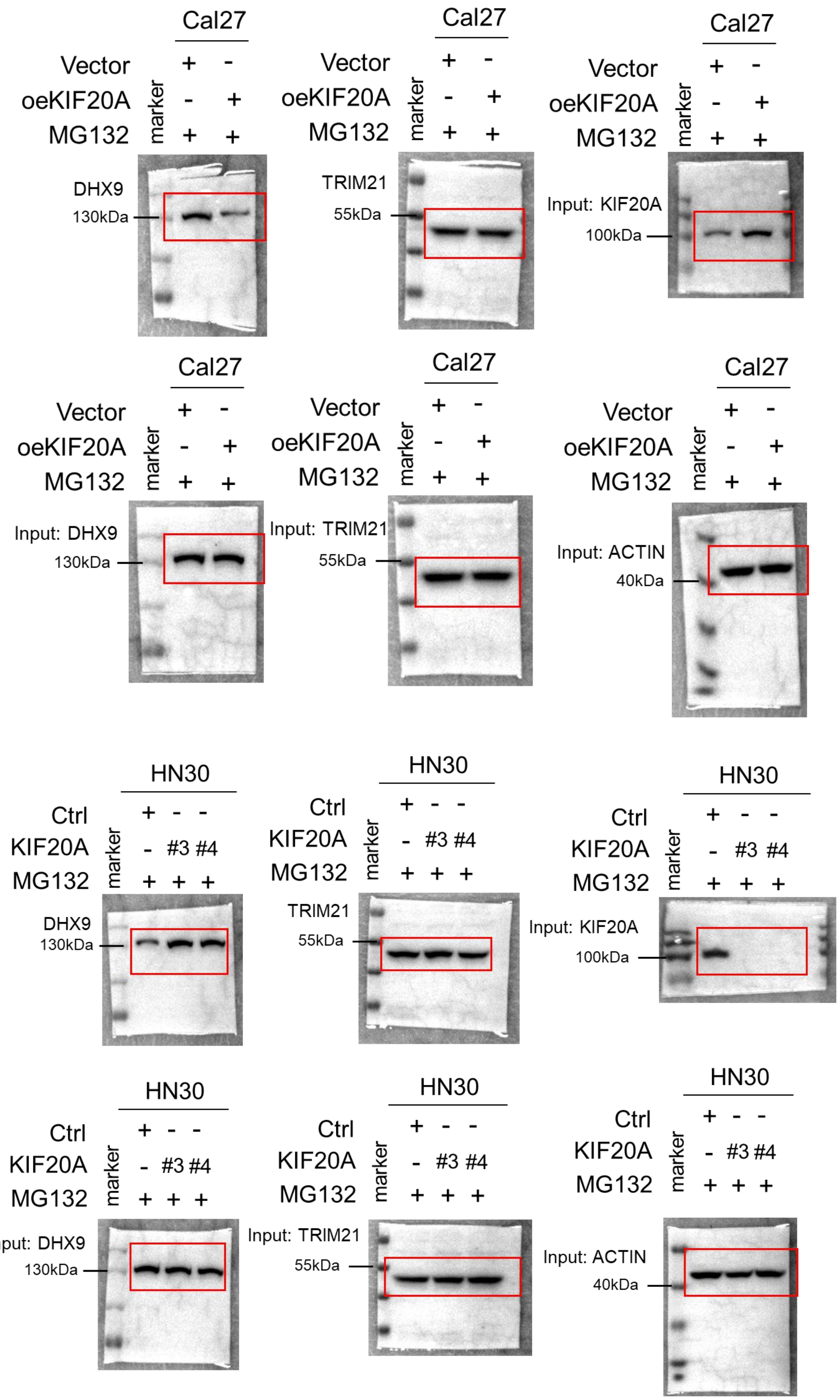
Cal27
Vector + -
oeKIF20A - +
MG132 + +
marker
DHX9
130kDa
Cal27
Vector + -
oeKIF20A - +
MG132 + +
marker
TRIM21
55kDa
Cal27
Vector + -
oeKIF20A - +
MG132 + +
marker
Input: KIF20A
100kDa
Cal27
Vector + -
oeKIF20A - +
MG132 + +
marker
Input: DHX9
130kDa
Cal27
Vector + -
oeKIF20A - +
MG132 + +
marker
Input: TRIM21
55kDa
Cal27
Vector + -
oeKIF20A - +
MG132 + +
marker
Input: ACTIN
40kDa
HN30
Ctrl + - -
KIF20A - #3 #4
MG132 + + +
marker
DHX9
130kDa
HN30
Ctrl + - -
KIF20A - #3 #4
MG132 + + +
marker
TRIM21
55kDa
HN30
Ctrl + - -
KIF20A - #3 #4
MG132 + + +
marker
Input: KIF20A
100kDa
HN30
Ctrl + - -
KIF20A - #3 #4
MG132 + + +
marker
Input: DHX9
130kDa
HN30
Ctrl + - -
KIF20A - #3 #4
MG132 + + +
marker
Input: TRIM21
55kDa
HN30
Ctrl + - -
KIF20A - #3 #4
MG132 + + +
marker
Input: ACTIN
40kDa

L

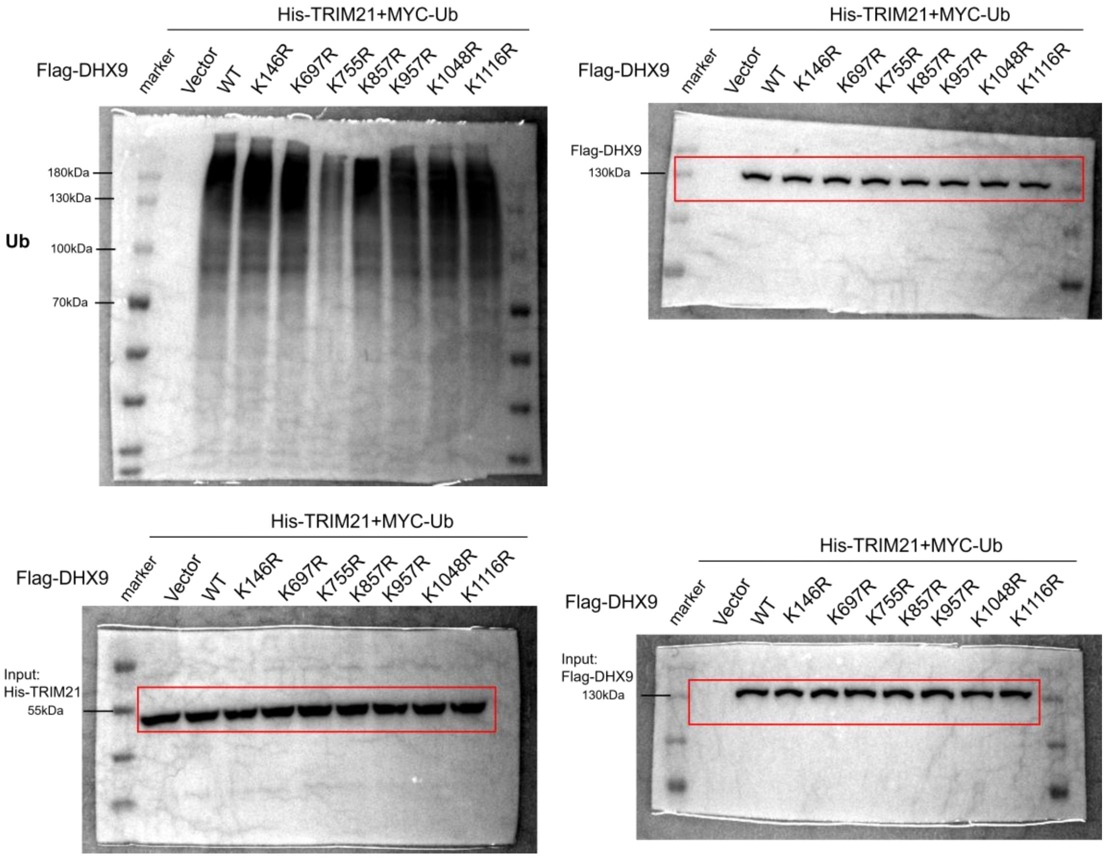
His-TRIM21+MYC-Ub
Flag-DHX9
marker
Vector
WT
K146R
K697R
K755R
K857R
K957R
K1048R
K1116R
Ub
180kDa
130kDa
100kDa
70kDa
Flag-DHX9
130kDa
Input:
His-TRIM21
55kDa
Input:
Flag-DHX9
130kDa

M

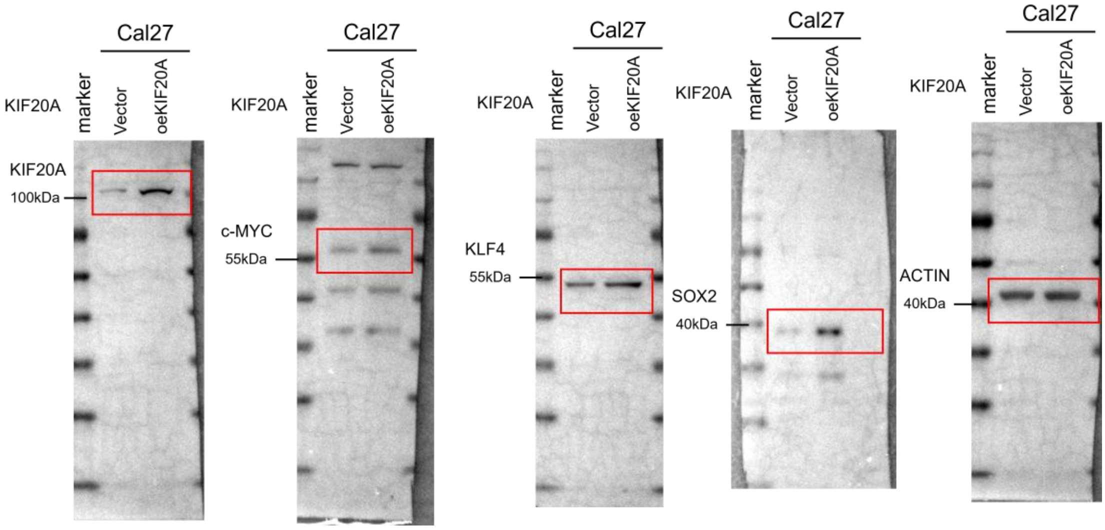
Cal27
KIF20A
marker
Vector
oeKIF20A
KIF20A
100kDa
c-MYC
55kDa
KLF4
55kDa
SOX2
40kDa
ACTIN
40kDa

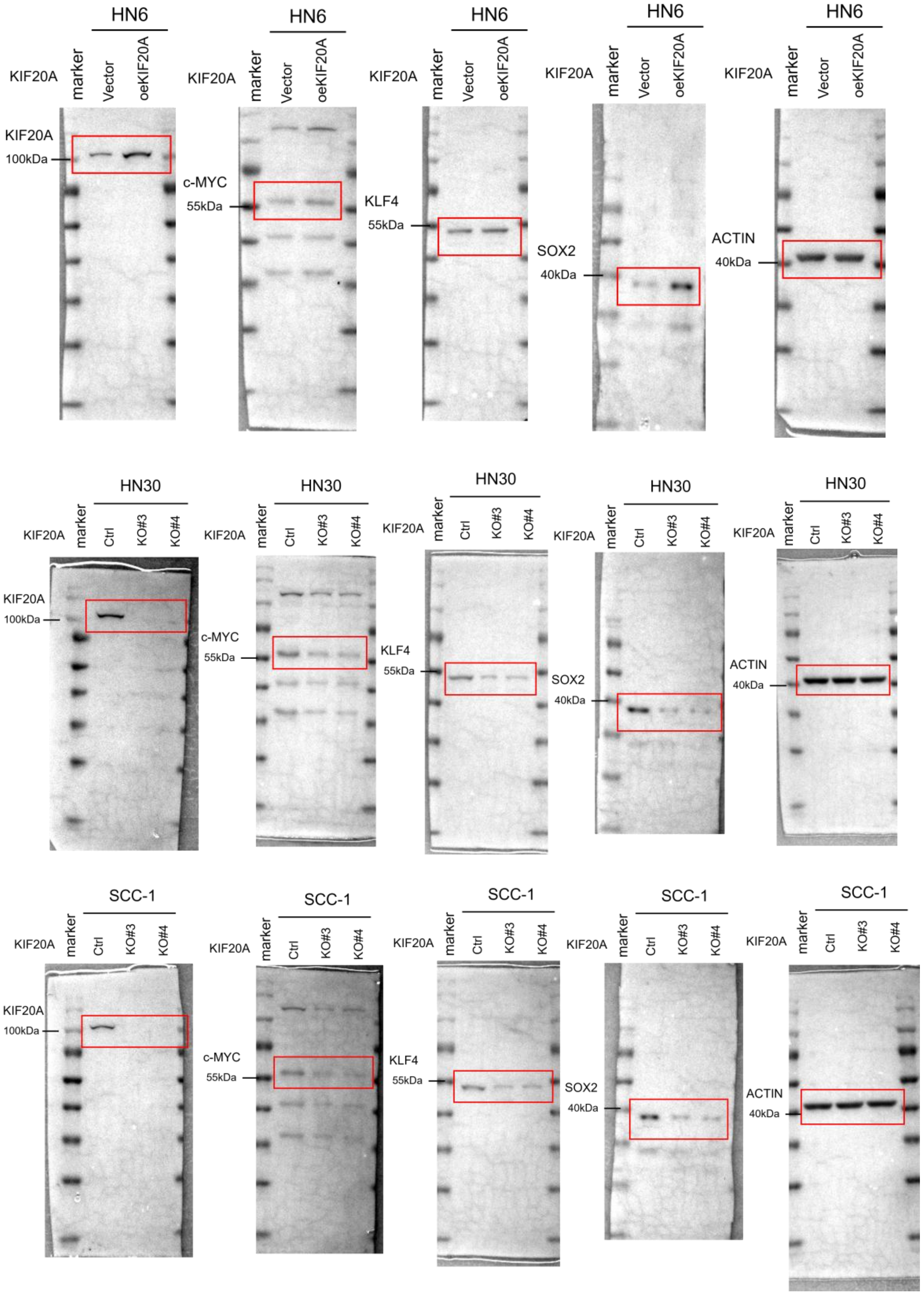
HN6
KIF20A
marker
Vector
oeKIF20A
KIF20A
100kDa
c-MYC
55kDa
KLF4
55kDa
SOX2
40kDa
ACTIN
40kDa
HN30
KIF20A
marker
Ctrl
KO#3
KO#4
KIF20A
100kDa
c-MYC
55kDa
KLF4
55kDa
SOX2
40kDa
ACTIN
40kDa
SCC-1
KIF20A
marker
Ctrl
KO#3
KO#4
KIF20A
100kDa
c-MYC
55kDa
KLF4
55kDa
SOX2
40kDa
ACTIN
40kDa

N

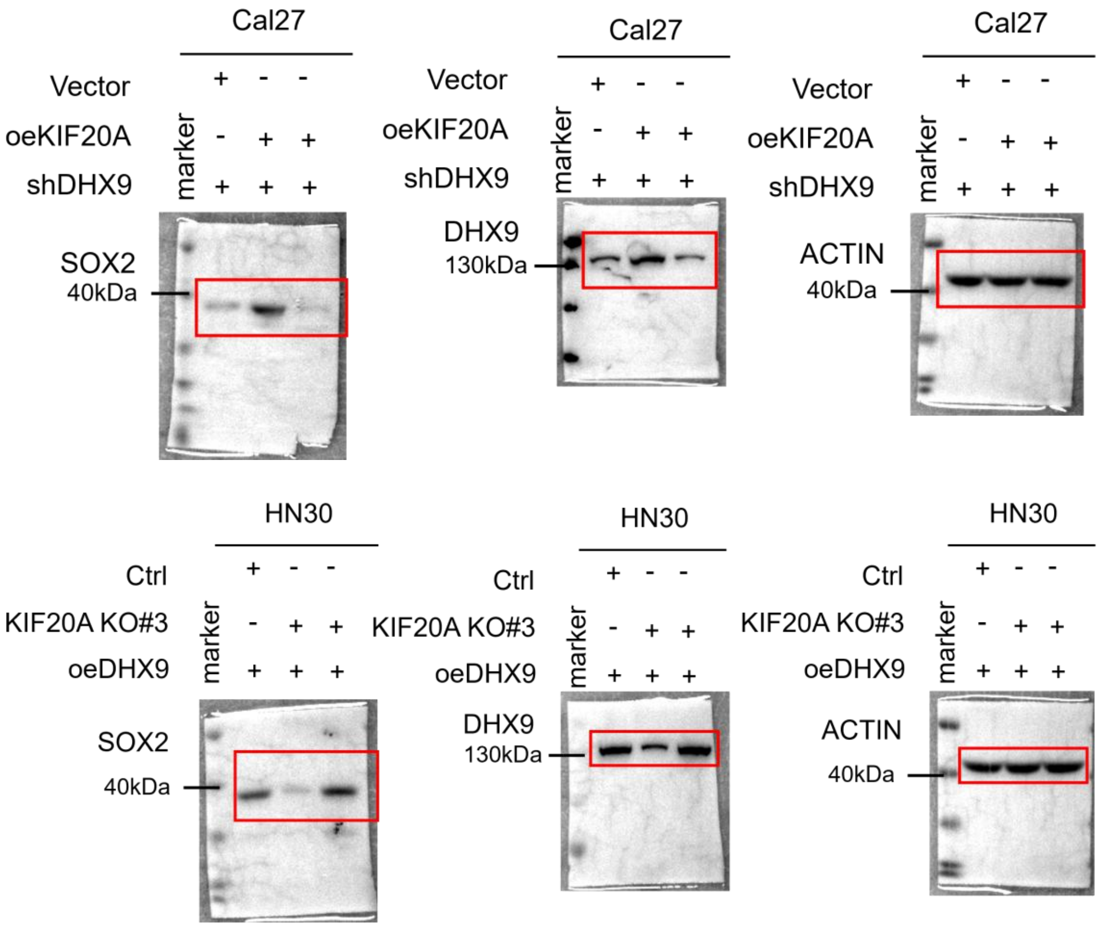
Cal27
Vector
oeKIF20A
shDHX9
marker
SOX2
40kDa
DHX9
130kDa
ACTIN
40kDa
HN30
Ctrl
KIF20A KO#3
oeDHX9
SOX2
40kDa
DHX9
130kDa
ACTIN
40kDa

O

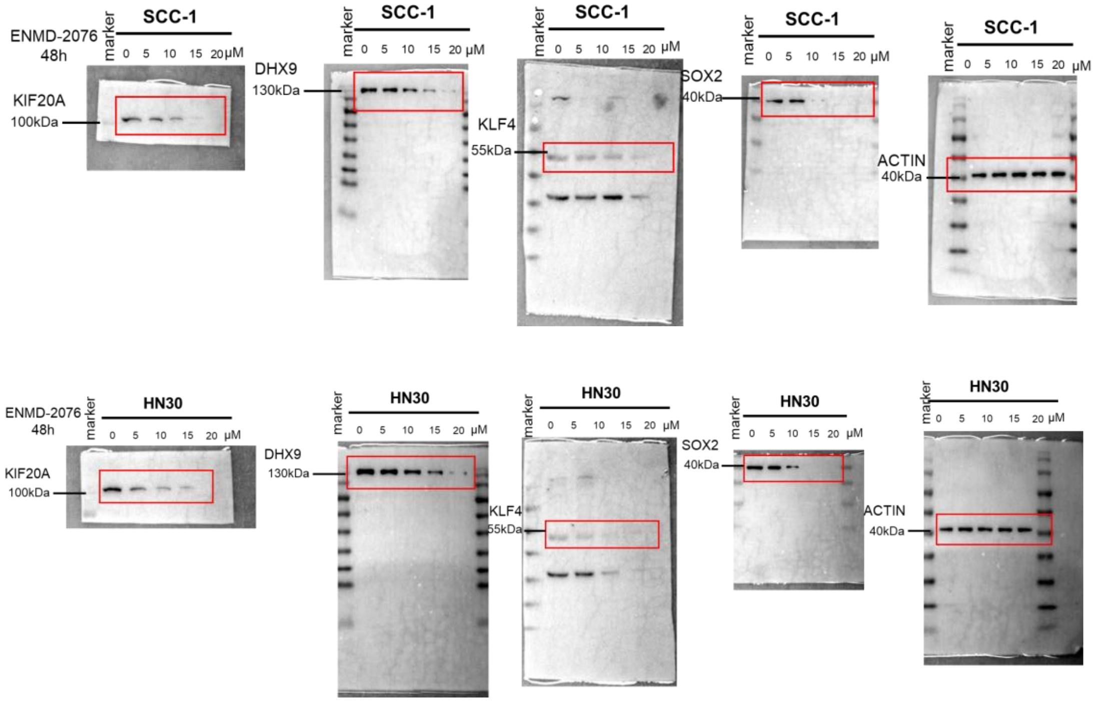
ENMD-2076
48h
marker
SCC-1
0 5 10 15 20 μM
KIF20A
100kDa
DHX9
130kDa
KLF4
55kDa
SOX2
40kDa
ACTIN
40kDa
HN30

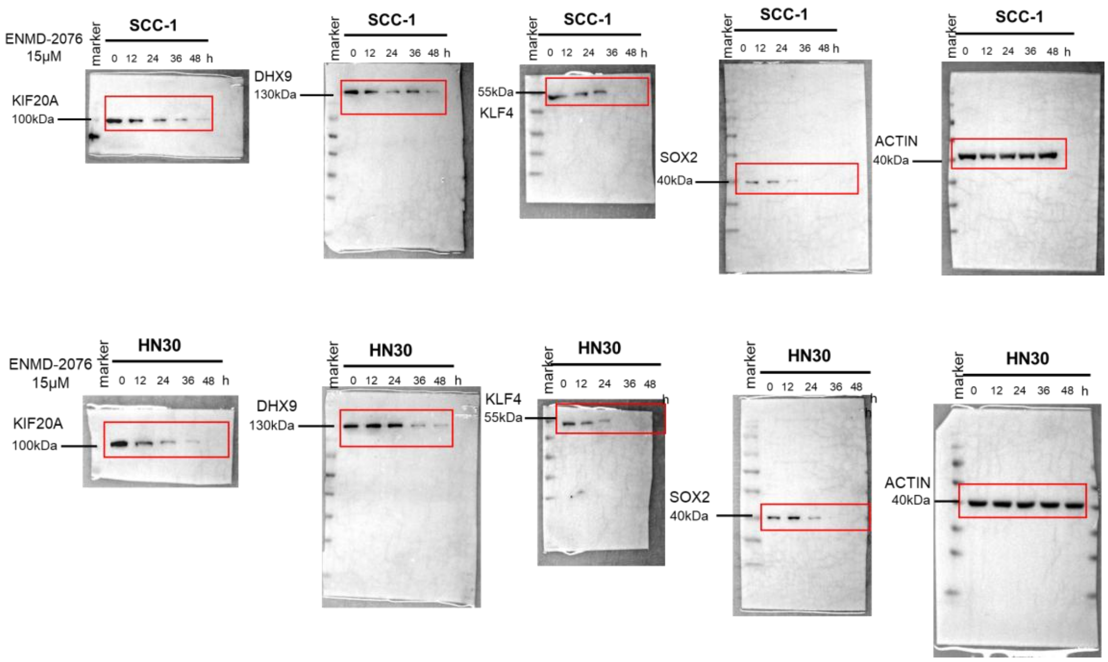
ENMD-2076
15μM
marker
SCC-1
0 12 24 36 48 h
KIF20A
100kDa
DHX9
130kDa
55kDa
KLF4
SOX2
40kDa
ACTIN
40kDa
HN30

P

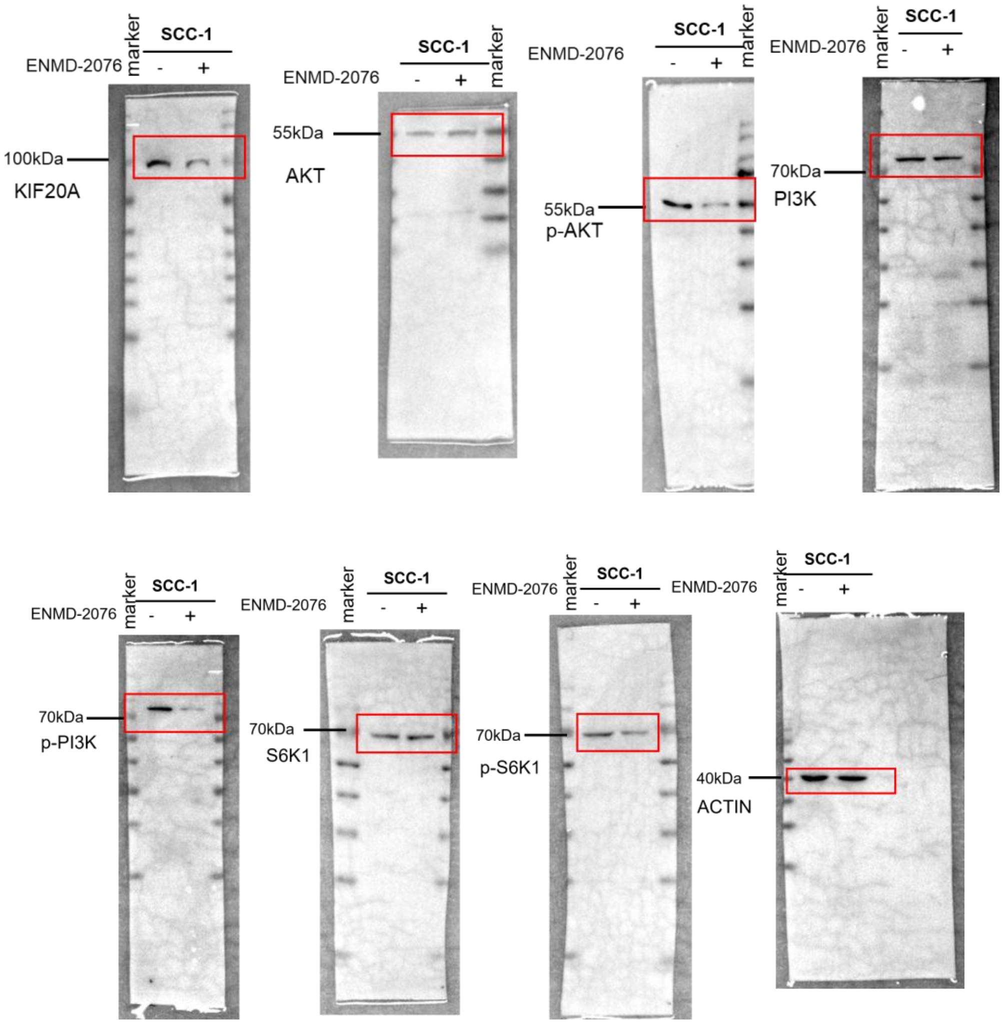
SCC-1
ENMD-2076
marker
- +
100kDa
KIF20A
55kDa
AKT
55kDa
p-AKT
70kDa
PI3K
70kDa
p-PI3K
70kDa
S6K1
70kDa
p-S6K1
40kDa
ACTIN

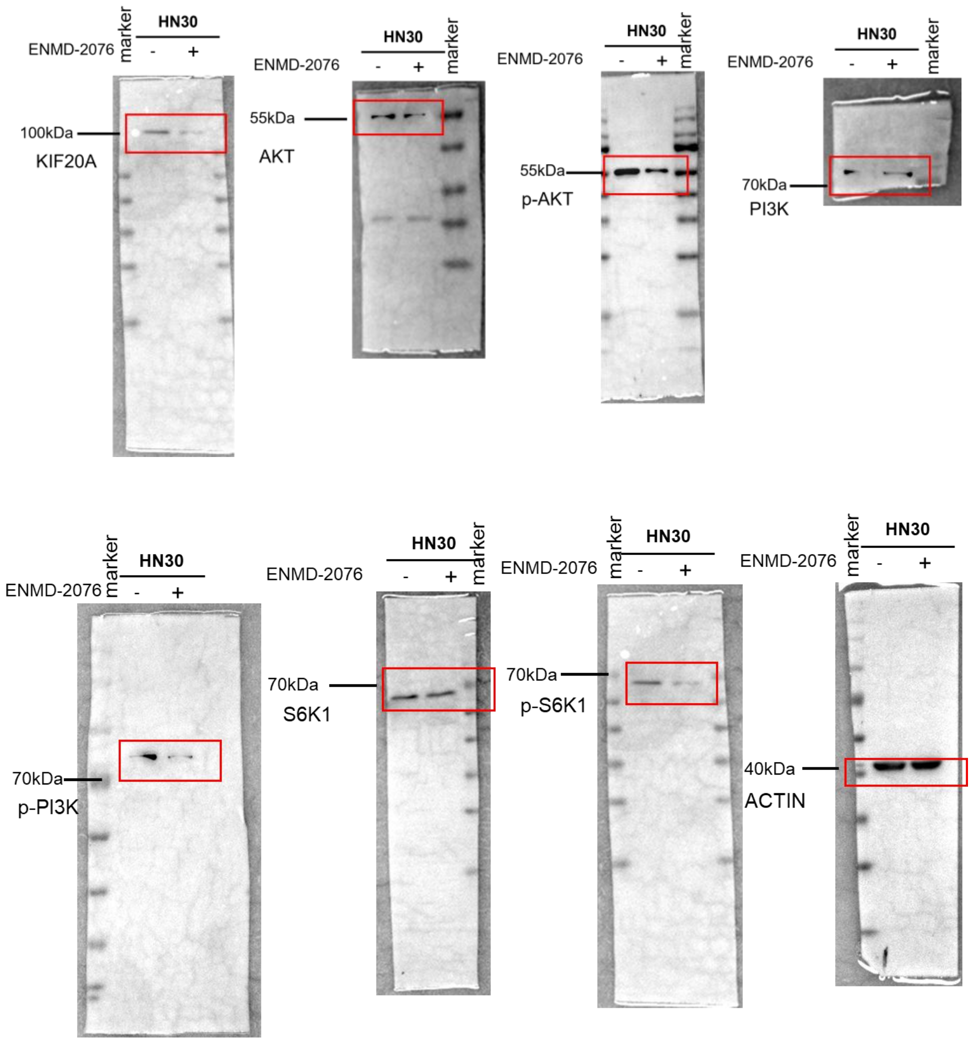
marker
HN30
ENMD-2076
-
+
100kDa
KIF20A
55kDa
AKT
p-AKT
70kDa
PI3K
p-PI3K
S6K1
p-S6K1
40kDa
ACTIN

Q

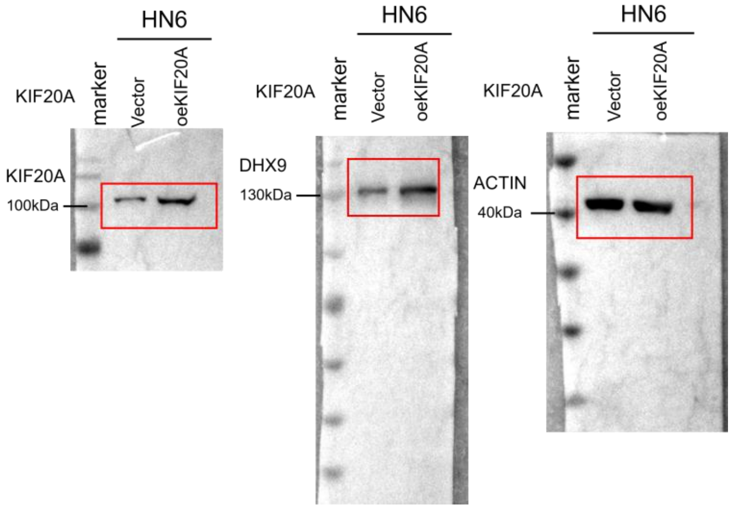
HN6
KIF20A
marker
Vector
oeKIF20A
100kDa
DHX9
130kDa
ACTIN
40kDa

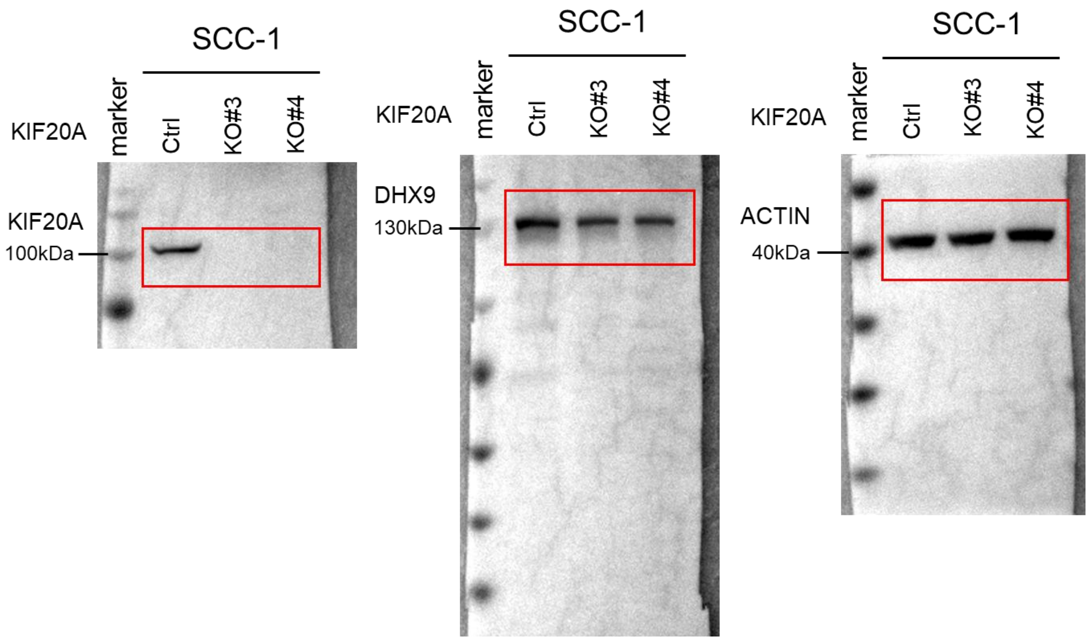
SCC-1
KIF20A
marker
Ctrl
KO#3
KO#4
KIF20A
100kDa
SCC-1
KIF20A
marker
Ctrl
KO#3
KO#4
DHX9
130kDa
SCC-1
KIF20A
marker
Ctrl
KO#3
KO#4
ACTIN
40kDa

R

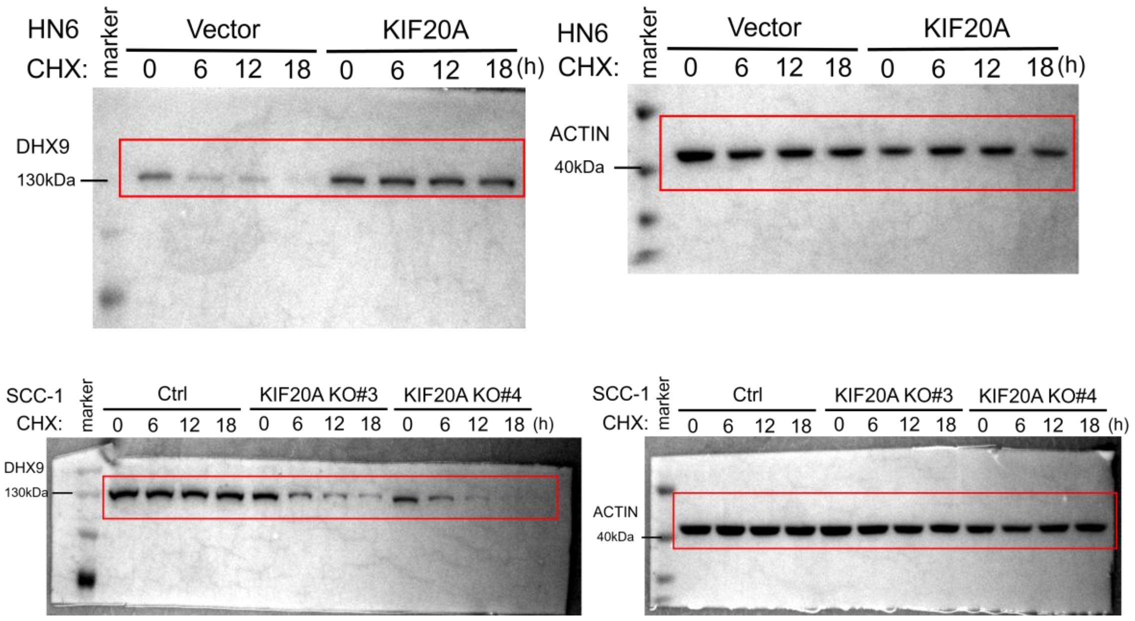
HN6
CHX:
marker
Vector
KIF20A
0 6 12 18 0 6 12 18 (h)
DHX9
130kDa
HN6
CHX:
marker
Vector
KIF20A
0 6 12 18 0 6 12 18 (h)
ACTIN
40kDa
SCC-1
CHX:
marker
Ctrl
KIF20A KO#3
KIF20A KO#4
0 6 12 18 0 6 12 18 0 6 12 18 (h)
DHX9
130kDa
SCC-1
CHX:
marker
Ctrl
KIF20A KO#3
KIF20A KO#4
0 6 12 18 0 6 12 18 0 6 12 18 (h)
ACTIN
40kDa

S

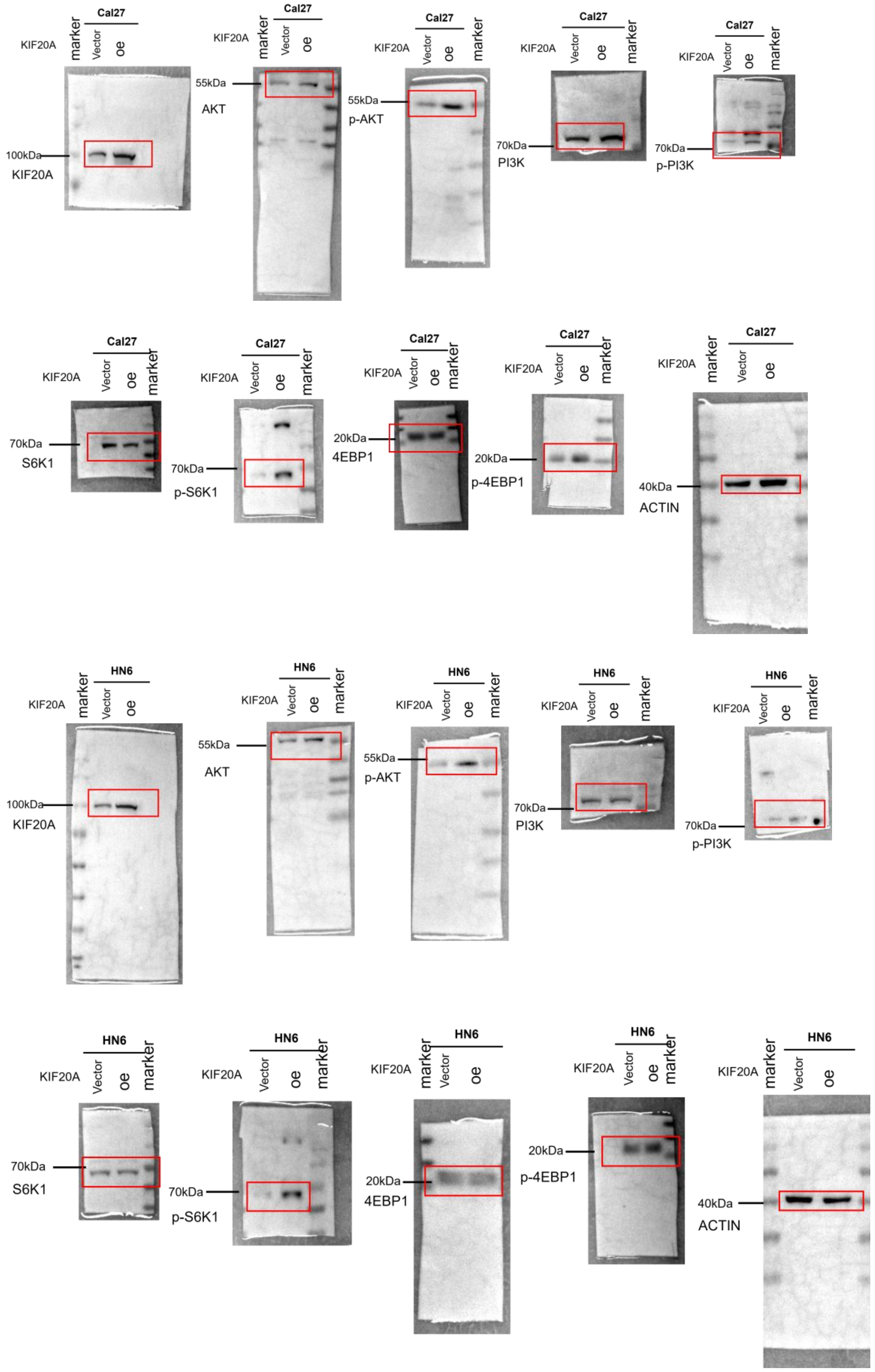
Cal27
KIF20A
marker
Vector
oe
100kDa
KIF20A
55kDa
AKT
55kDa
p-AKT
70kDa
PI3K
70kDa
p-PI3K
70kDa
S6K1
70kDa
p-S6K1
20kDa
4EBP1
20kDa
p-4EBP1
40kDa
ACTIN
HN6
100kDa
KIF20A
55kDa
AKT
55kDa
p-AKT
70kDa
PI3K
70kDa
p-PI3K
70kDa
S6K1
70kDa
p-S6K1
20kDa
4EBP1
20kDa
p-4EBP1
40kDa
ACTIN

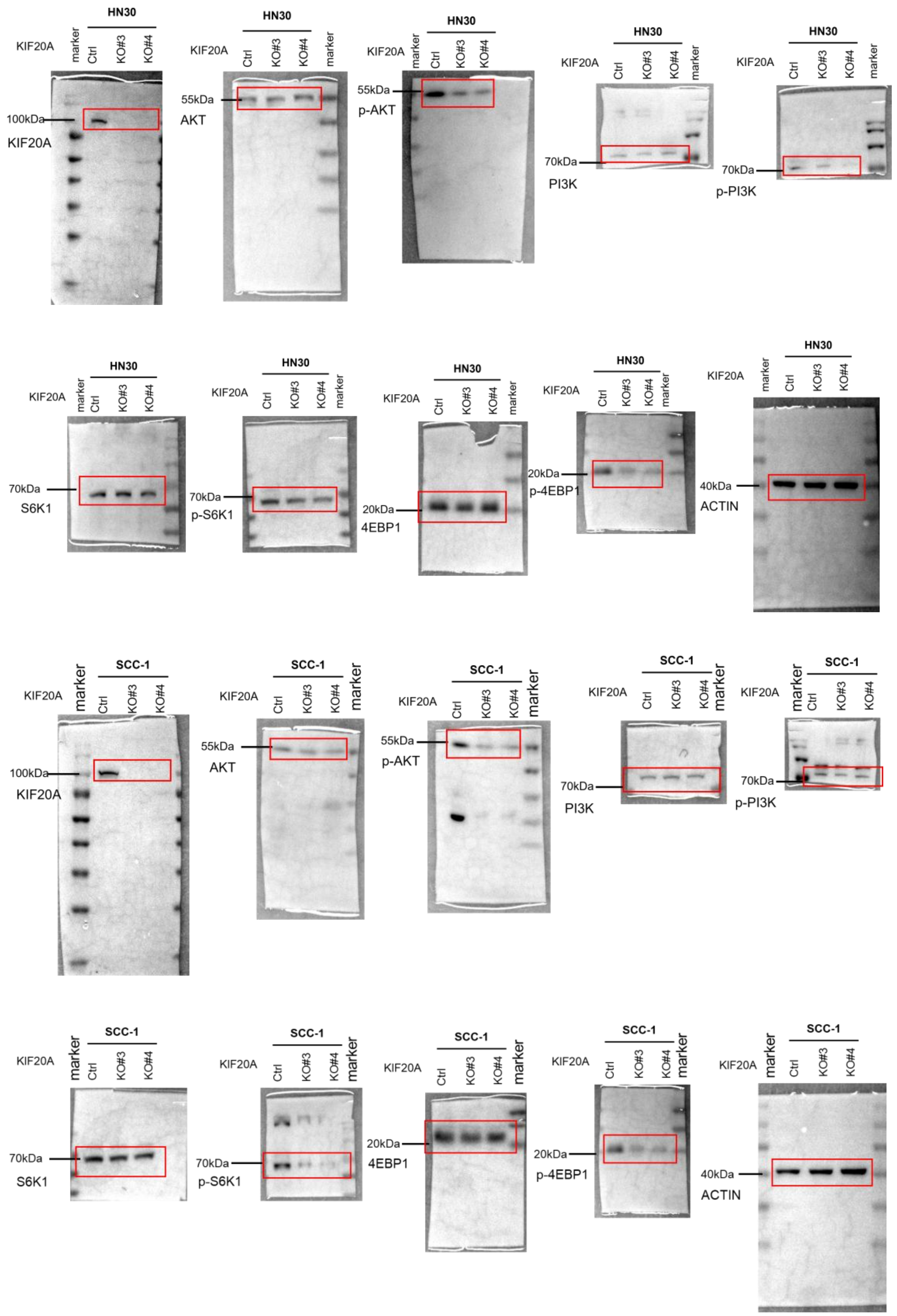
HN30
KIF20A
marker
Ctrl
KO#3
KO#4
100kDa
KIF20A
55kDa
AKT
55kDa
p-AKT
70kDa
PI3K
70kDa
p-PI3K
70kDa
S6K1
70kDa
p-S6K1
20kDa
4EBP1
20kDa
p-4EBP1
40kDa
ACTIN
SCC-1
KIF20A
marker
Ctrl
KO#3
KO#4
100kDa
KIF20A
55kDa
AKT
55kDa
p-AKT
70kDa
PI3K
70kDa
p-PI3K
70kDa
S6K1
70kDa
p-S6K1
20kDa
4EBP1
20kDa
p-4EBP1
40kDa
ACTIN

T

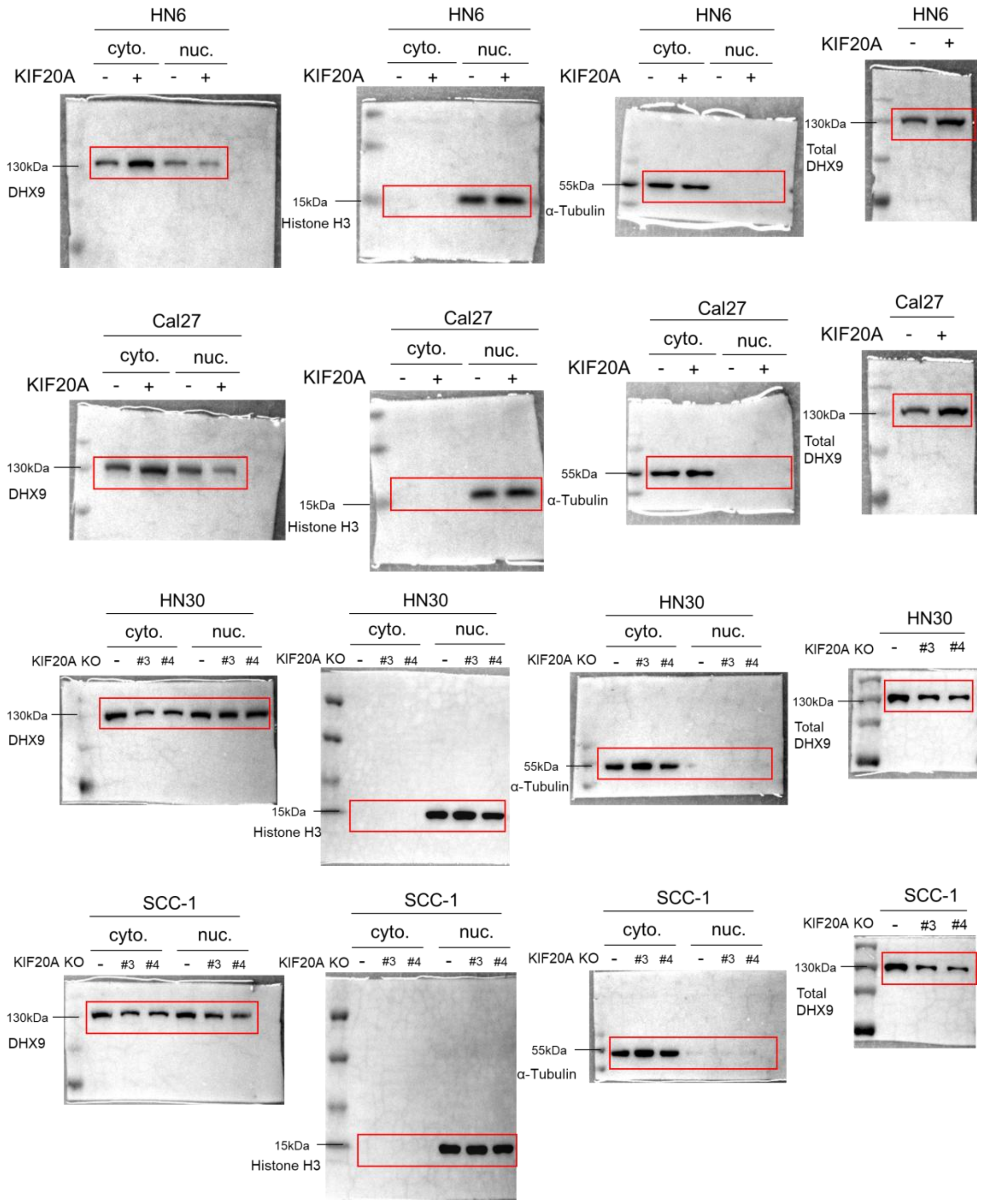

Uncropped western blots. **(A)** Uncropped western blots for main Figure 2B. **(B)** Uncropped western blots for main Figure 2D. **(C)** Uncropped western blots for main Figure 2E. **(D)** Uncropped western blots for main Figure 2G,2H. **(E)** Uncropped western blots for main Figure 3A,3B. **(F)** U Uncropped western blots for main Figure 3C. **(G)** Uncropped western blots for main Figure 3D. **(H)** Uncropped western blots for main Figure 4B. **(I)** Uncropped western blots for main Figure 4C. **(J)** Uncropped

western blots for main Figure 4D. **(K)** Uncropped western blots for main Figure 4E. **(L)** Uncropped western blots for main Figure 4F. **(M)** Uncropped western blots for main Figure 5E. **(N)** Uncropped western blots for main Figure 7C. **(O)** Uncropped western blots for main Figure 8B. **(P)** Uncropped western blots for main Figure 8C. **(Q)** Uncropped western blots for main Figure S2A. **(R)** Uncropped western blots for main Figure S3A,S3B. **(S)** Uncropped western blots for main Figure S7B. **(T)** Uncropped western blots for main Figure S9.
